# NCF2, MYO1F, S1PR4, and FCN1 as potential noninvasive diagnostic biomarkers in patients with obstructive coronary artery: A weighted gene co‐expression network analysis

**DOI:** 10.1002/jcb.29128

**Published:** 2019-06-27

**Authors:** Xian‐Gang Mo, Wei Liu, Yao Yang, Saber Imani, Shan Lu, Guorong Dan, Xuqiang Nie, Jun Yan, Rixing Zhan, Xiaohui Li, Youcai Deng, Bingbo Chen, Yue Cai

**Affiliations:** ^1^ Department of Geriatrics The Affiliated Hospital of Guizhou Medical University Guiyang Guizhou China; ^2^ Health Physical Examination Center The Affiliated Hospital of Qingdao University Qingdao Shandong China; ^3^ Institute of Materia Medica, College of Pharmacy Army Medical University (Third Military Medical University) Chongqing China; ^4^ Department of Oncology The Affiliated Hospital of Southwest Medical University Luzhou Sichuan China; ^5^ Center of Hepatobiliary Pancreatic Disease Beijing Tsinghua Changgung Hospital Beijing China; ^6^ Institute of Burn Research, Southwest Hospital Army Medical University (Third Military Medical University) Chongqing China; ^7^ Laboratory Animal Center Army Medical University (Third Military Medical University) Chongqing China; ^8^ Department of Cardiology, Xijing Hospital Fourth Military Medical University Xi'an Shaanxi China

**Keywords:** FCN1, MYO1F, NCF2, noninvasive diagnostic biomarkers, obstructive coronary artery disease, S1PR4

## Abstract

This study aims to explore the predictive noninvasive biomarker for obstructive coronary artery disease (CAD). By using the data set GSE90074, weighted gene co‐expression network analysis (WGCNA), and protein–protein interactive network, construction of differentially expressed genes in peripheral blood mononuclear cells was conducted to identify the most significant gene clusters associated with obstructive CAD. Univariate and multivariate stepwise logistic regression analyses and receiver operating characteristic analysis were used to predicate the diagnostic accuracy of biomarker candidates in the detection of obstructive CAD. Furthermore, functional prediction of candidate gene biomarkers was further confirmed in ST‐segment elevation myocardial infarction (STEMI) patients or stable CAD patients by using the datasets of GSE62646 and GSE59867. We found that the blue module discriminated by WGCNA contained 13 hub‐genes that could be independent risk factors for obstructive CAD (*P* < .05). Among these 13 hub‐genes, a four‐gene signature including neutrophil cytosol factor 2 (NCF2, *P* = .025), myosin‐If (MYO1F, *P* = .001), sphingosine‐1‐phosphate receptor 4 (S1PR4, *P* = .015), and ficolin‐1 (FCN1, *P* = .012) alone or combined with two risk factors (male sex and hyperlipidemia) may represent potential diagnostic biomarkers in obstructive CAD. Furthermore, the messenger RNA levels of NCF2, MYO1F, S1PR4, and FCN1 were higher in STEMI patients than that in stable CAD patients, although S1PR4 showed no statistical difference (*P* > .05). This four‐gene signature could also act as a prognostic biomarker to discriminate STEMI patients from stable CAD patients. These findings suggest a four‐gene signature (NCF2, MYO1F, S1PR4, and FCN1) alone or combined with two risk factors (male sex and hyperlipidemia) as a promising prognostic biomarker in the diagnosis of STEMI. Well‐designed cohort studies should be implemented to warrant the diagnostic value of these genes in clinical purpose.

## Graphical abstract

A four‐gene signature (NCF2, MYO1F, S1PR4, and FCN1) alone or combined with two risk factors (male sex and hyperlipidemia) could act as a noninvasive diagnostic biomarker for obstructive coronary artery disease (CAD) and ST‐segment elevation myocardial infarction (STEMI).

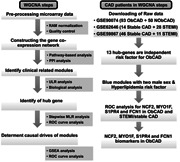

## 1 INTRODUCTION

Cardiovascular diseases (CVDs) continue to be the leading cause of morbidity and mortality worldwide.^1^ Epidemiological investigations have indicated that age is an independent risk factor for CVDs.^2^ Moreover, recent studies have revealed that patients with age over 65 years presenting with obstructive coronary artery disease (CAD) usually portend poorer outcomes compared with younger individuals, including a higher rate of 5‐year mortality, all‐cause mortality, recurrent myocardial infarction, and stroke.^3^, ^4^ Early‐stage detection and diagnosis of obstructive CAD can reduce the mortality ratio, especially in younger individuals.^5^ Certainly, a better understanding of the pathogenesis of obstructive CAD could help the development of effective therapeutic interventions, resulting warranted to decrease mortality and improve the patient's quality of life.

CAD is a chronic inflammatory disease and inflammation is the response of the immune system to the presence of exogenous and endogenous antigens. Previous studies have suggested that the proinflammatory response plays critical roles in the pathogenesis of obstructive CAD, including both the innate and adaptive immune responses,^6^, ^7^ which contribute to the plaque instability.^8^ The proinflammatory response is mainly mediated by the activation of peripheral blood mononuclear cells (PBMCs), followed by their migration to local vascular tissues.^9^ Therefore, screening diagnostic or prognostic biomarkers based on high‐throughput expression profiles of PBMC is a powerful weapon for obstructive CAD diagnosis and prognosis.

Recently, Ravi et al^10^ have reported that the proinflammatory chemokine, CXCL5, in PBMCs, played a protective role in obstructive CAD and was associated with the severity of CAD in geriatric patients. In this study, they contributed a data set of the whole genome messenger RNA (mRNA) expression profiles of PBMCs and clinical characteristics of 143 samples (93 subjects with obstructive CAD and 50 subjects free of obstructive CAD; data set: GSE90074).^10^ This data set provides us an opportunity to search novel potential biomarkers for obstructive CAD. Despite the numerous studies concerning the obstructive CAD pathogenesis, noninvasive diagnostic biomarkers with high sensitivity and specificity for early‐stage obstructive CAD detection, are still needed.

Therefore, in this current study, differentially expressed genes (DEGs) in PBMCs between patients with obstructive CAD and free of obstructive CAD were identified, following by Gene Ontology (GO) and Kyoto Encyclopedia of Genes and Genomes (KEGG) pathway enrichment analysis. Subsequently, through unsupervised hierarchical clustering and weighted gene co‐expression network analysis (WGCNA), the relationship between gene sets and phenotypes of CAD was ascertained. By using the bioinformatics methods and receiver operating characteristic (ROC) analysis, a potential four‐gene signature including ficolin‐1 (FCN1), myosin‐If (MYO1F), neutrophil cytosol factor 2 (NCF2), and sphingosine‐1‐phosphate receptor 4 (S1PR4), were further identified as biomarkers to discriminate obstructive CADs from nonobstructive CADs. Furthermore, the accuracy of this four‐gene signature was further explored for its accuracy to discriminate ST‐segment elevation myocardial infarction (STEMI) patients from stable CAD patients as well as may offer the potential novel therapeutic strategies for stable CADs.

## 2 MATERIALS AND METHODS

### 2.1 Microarray data set information

In this study, the data set GSE90074 (deposited by Saranya Ravi et al)^10^ was retrieved and downloaded from the Gene Expression Omnibus (GEO) database in the National Center of Biotechnology Information (NCBI), based on the platform of Affymetrix Human Genome U133 Plus 2.0 Array (Santa Clara, CA).^10^ This data set includes the mRNA expression data of PBMCs and clinical characteristics of 143 samples (93 subjects with obstructive CAD and 50 subjects without obstructive CAD). Most of those diagnosed with obstructive CAD were men (61%) and had a previous diagnosis of hyperlipidemia (80%) or myocardial infarction (MI; 44%).

For the validation of the WGCNA findings in obstructive CAD, the gene expression data of PBMCs from STEMI patients were also retrieved from the NCBI GEO database. The data set, GSE62646, includes 14 PBMCs samples from stable CAD patients as control and 28 PBMCs samples from STEMI patients.^11^ The data set, GSE59867, includes 46 PBMCs samples from stable CAD patients as control and 11 PBMCs samples from STEMI patients.^12^

### 2.2 Identification of differentially expressed genes

After normalizing the data, analysis of DEGs was performed using the package limma of R (version 3.3.3), and the gene with *P‐*values less than .05 was considered as a statistically significant DEG. The heatmap was visualized by using the heatmap package for “R” statistical software (version 3.3.3), as described previously.^13^, ^14^, ^15^

### 2.3 Enrichment analysis

KEGG pathway and GO, including cellular component, molecular function, and biological process, were analyzed using the package clusterProfiler (version 3.2.14) of R (version 3.3.3), as described previously.^13^, ^14^

### 2.4 Weighted gene co‐expression network analysis

WGCNA package (version 1.60) in R was used to identify key modules based on the expression levels of DEGs in the data set GSE90074. The module is a cluster of closely interconnected genes, based on the dendrogram height. Modules were detected by using unsupervised clustering and dynamic branch cut methods (WGCNA: an R package for weighted correlation network analysis).^15^ The gene modules were signified by different colors and the gray module showed the genes that cannot be merged. Weight =0.7 and power of β =5 (scale free R^2^ = 0.8) were used to construct modules, and a threshold ≥0.7 was used to export network to Cytoscape (Figure S1).

### 2.5 Protein–protein interactive network construction and hub‐gene identification

Search Tool for the Retrieval of Interacting Genes/Proteins (STRING; https://string‐db.org/) was used to evaluate the protein–protein (PPI) network among genes in the enriched modules. PPI networks were constructed using Cytoscape (version 3.6.0), as described previously.^15^ The gene with PPI network nodes and co‐expression network nodes ≥5 was identified as the hub‐gene.

### 2.6 Gene set enrichment analysis

Gene set enrichment analysis (GSEA), including KEGG and GO enrichment analysis, is an effective method to compare the significant different priori defined sets of two groups. GSEA was performed with the gene expression of NCF2, MYO1F, S1PR4, and FCN1 in obstructive CAD patients using phenotype labels “high‐expression” vs “low‐expression” group by the GSEA software (http://software.broadinstitute.org/gsea/index.jsp), as described previously.^15^ Gene sets used in this study were c2.cp.kegg.v5.2.symbols.gmt downloaded from the Molecular Signatures Database (MSigDB; http://software.broadinstitute.org/gsea/msigdb/index.jsp). *P* < .05 was used as the cut‐off criterion.

### 2.7 Statistical analysis

All statistical analyses were carried out using “R” software (version 3.3.3). Univariate and stepwise multivariate logistic regression (MLR) analyses were carried out to determine independent factors for the diagnosis of CAD by using the hub‐genes in the blue module and clinical risk factors. Receiver operating characteristic (ROC) curve analysis was used to calculate the value of area under the curve (AUC) for the selected genes and clinical risk factors to evaluate their predictive abilities for the diagnosis of CAD by using the package pROC, as described previously.^5^ Here, *P* < .05 was considered statistically significant.

## 3 RESULTS

### 3.1 Identification and enrichment analysis of DEGs

**Figure 1:**
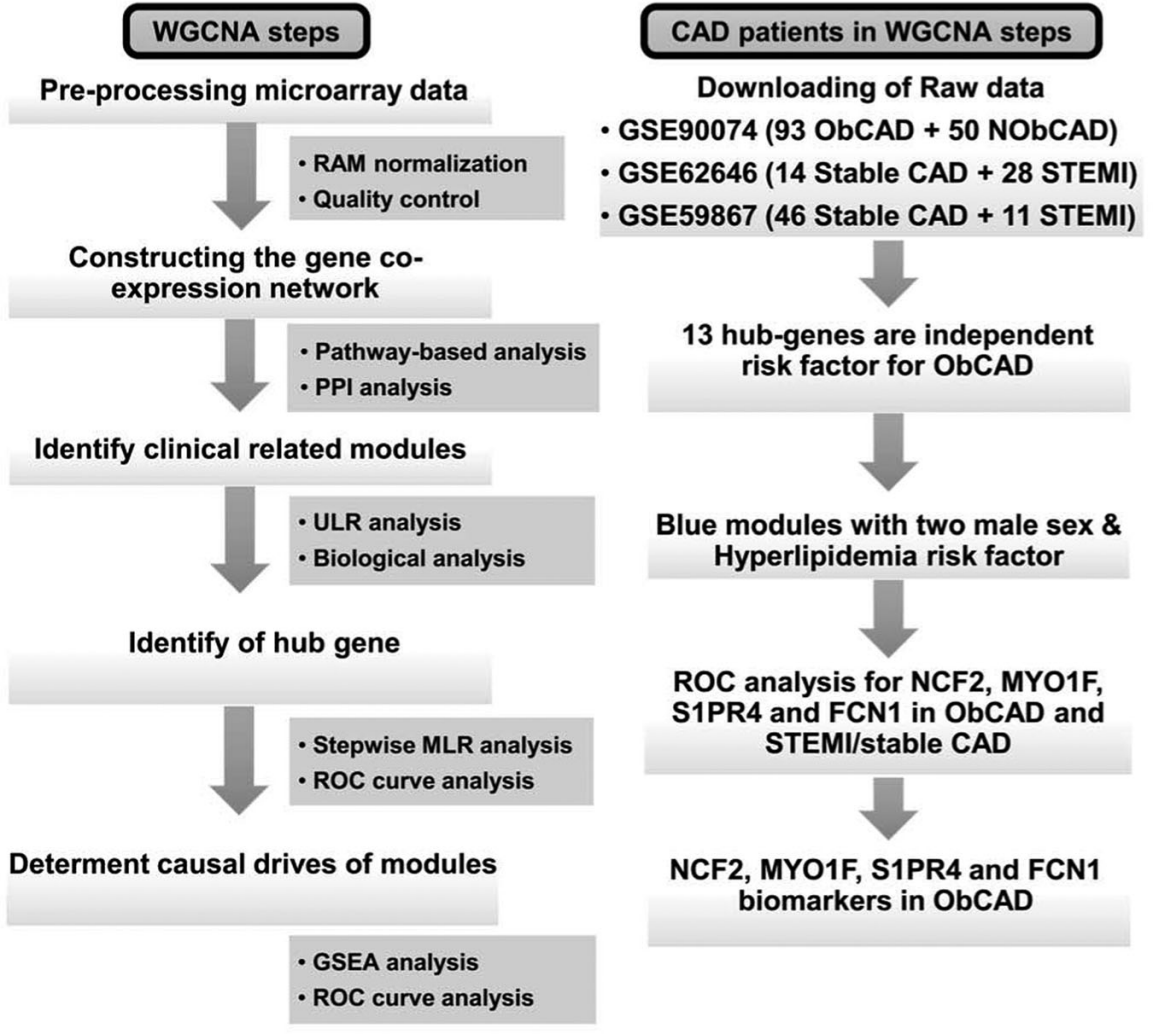
Flowchart describing the schematic overview of the current study design. After enrichment analysis and weighted gene co‐expression network analysis (WGCNA) of differentially expressed genes (DEGs), we identified the blue module as the key module. Then, through construction of co‐expression and Protein–protein (PPI) network for the blue module, we identified 13 hub‐genes in the blue module. By using univariate logistic regression (ULR) analysis, all the 13 hub genes can be independent risk factors for obstructive coronary artery disease (CAD). Stepwise multivariate logistic regression (MLR) analysis was used to identify the preferred model among the hub‐genes, and the receiver operating characteristic (ROC) curve analysis was used to evaluate the accuracy of genes in the identified preferred model. The expression levels and ROC curve analysis of genes in the preferred model were further analyzed in stable CAD and ST‐segment elevation myocardial infarction (STEMI) patients. At last, gene set enrichment analysis (GSEA) was used to predict the potential mechanisms of identified biomarkers in the development of obstructive CAD. In all, this four gene‐signature could be a good biomarker for both obstructive CAD and STEMI

**Figure 2:**
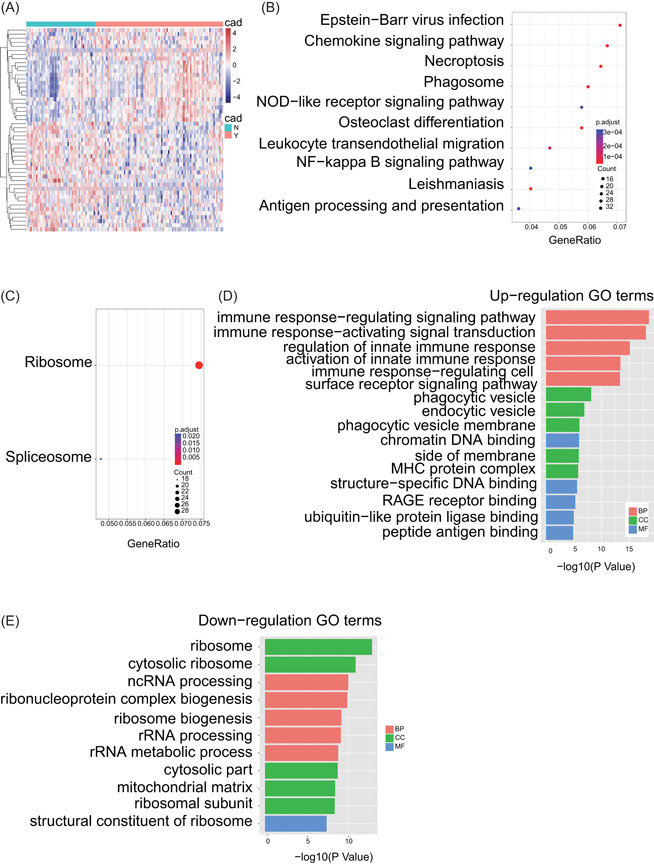
Identification and enrichment analysis of DEGs in peripheral blood mononuclear cells (PBMCs) between patients with or without obstructive CAD. A, Heatmap of the DEGs (top 25 upregulated genes and 25 downregulated genes). Each row represents the messenger RNA (mRNA) and each column represents one sample which annotated by a different color, respectively. The expression level of each mRNA in one sample is represented in the shade of red or blue, which represents upregulated or downregulated genes, respectively. B,C, Kyoto Encyclopedia of Genes and Genomes (KEGG) enrichment analysis of the upregulated (B) and downregulated genes (C). The size and the color intensity of a circle represent the numbers of enriched genes and −log 10 (*P*‐value), respectively. D,E, Gene Ontology (GO) enrichment analysis of upregulated (D) and downregulated genes (E). The vertical and horizontal axes represent GO term and −log 10 (*P*‐value) of the corresponding GO term, respectively. Different colors reflect main categories of GO terms: BP, biological process; CC, cellular component; MF, molecular function. CAD, coronary artery disease; DEG, differentially expressed gene

To find diagnostic biomarkers for obstructive CAD, we set up a workflow shown in Figure 1. Based on the WGCNA pipeline we modified, 1193 downregulation genes and 1041 upregulation genes were identified in PMBCs from patients between obstructive CAD and free of obstructive CAD (Table S1). The top 50 significantly upregulated and downregulated genes are listed in Figure 2A. KEGG pathway enrichment analysis showed that the most significant upregulated genes were mainly involved in inflammatory response, such as Epstein‐Barr virus infection, chemokine signaling pathway, necroptosis, phagosome, NOD‐like receptor signaling pathway, nuclear factor‐κB signaling pathway,^16^ leukocyte transendothelial migration,^17^ and antigen processing and presentation^18^ (Figure 2B). All of these pathways were reported with important roles in CAD. However, only ribosome and spliceosome pathways were enriched in the most significant downregulated genes (Figure 2C). GO analysis showed that the top five upregulated biology processes were mainly involved in immune response, such as immune response‐regulating signaling pathway, immune response‐activating signaling transduction, regulation of innate immune response, activation of innate immune response, and immune response‐regulating cell surface signaling pathway (Figure 2D). Likewise, the top five downregulated biology processes were mainly involved in RNA processing and biogenesis, such as noncoding RNA processing, ribonucleoprotein complex biogenesis, ribosome biogenesis, rRNA processing, and rRNA metabolic process (Figure 2E). Together, both above KEGG and GO pathway enrichment analysis indicated that these significant upregulation genes were mainly involved in the immune responses, which would contribute to atherogenesis.^19^, ^20^

### 3.2 Module constructing and screening

**Figure 3:**
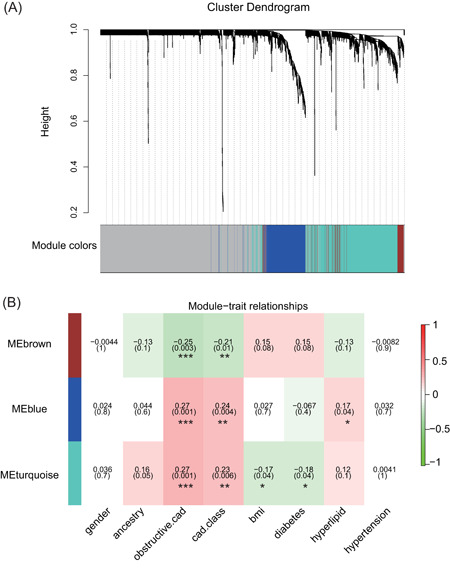
Modules enriched by WGCNA analysis and their correlation with clinical traits. A, Gene clustering and module identification by WGCNA analysis based on the data set GSE90074. Top: the result of hierarchical clustering was shown in clustering dendrogram. Each line represents one gene. Bottom: Different colors below the dendrogram represent different co‐expression module for the significant genes. B, Heatmap shows the correlation between each module and each clinical trait. Each cell contained the corresponding correlation index and *P*‐value for each pair of module and clinical trait. *P < .05; **P < .01; ***P < .005 (Student *t* test). WGCNA, weighted gene co‐expression network analysis

To search potentially key genes associated with obstructive CAD, we performed WGCNA with the most significant 2234 genes mentioned above to identify key modules of highly correlated genes. First, the hierarchical clustering tree (dendrogram) resulted in three significant modules with various colors, including blue, brown, and turquoise modules (Figure 3A, Table S2). Furthermore, the analysis of the correlation between modules and clinical characteristics showed that all three significant modules were significantly correlated with CAD obstruction and class (Figure 3B). In addition, the turquoise module is correlated with diabetes and body mass index (BMI), and the blue module showed a significant correlation with hyperlipid (Figure 3B). This suggested that the tree highly preserved modules may be closely associated with BMI and hyperlipid of obstructive CAD.

**Figure 4:**
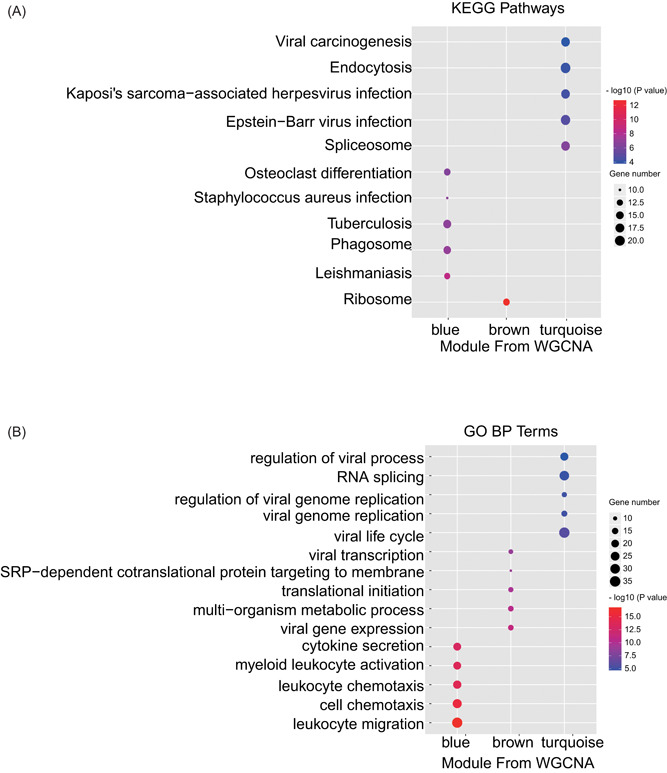
Enrichment analysis of different modules identified by WGCNA in Figure 2A. A, KEGG enrichment analysis of the enriched modules. B, GO BP enrichment analysis of the enriched modules. The size and color intensity of a circle represent the numbers of genes and −log 10 (*P*‐value) for each module, respectively. BP, biological process; GO, Gene Ontology; KEGG, Kyoto Encyclopedia of Genes and Genomes; WGCNA, weighted gene co‐expression network analysis

Further, KEGG pathway enrichment analysis showed that the blue module was mainly involved in osteoclast differentiation, tuberculosis, and phagosome; while the turquoise module, in, viral carcinogenesis, endocytosis, and Epstein‐Barr virus infection (Figure 4A). Furthermore, GO analysis showed that the top five biology processes in the blue module were mainly involved in leukocyte migration, cell chemotaxis, leukocyte chemotaxis, myeloid leukocyte activation, and cytokine secretion, indicative of leukocyte stimulation and migration in patients of obstructive CAD. KEGG and GO pathway analyses of the genes in the blue module showed similar findings as those by the total significant genes, which indicated that genes in the blue module played critical roles in the development of obstructive CAD by involving enhanced leukocyte activation and migration (Figure 4B).^21^

### 3.3 Detection of potential key messenger RNAs associated with obstructive coronary artery disease

**Figure 5:**
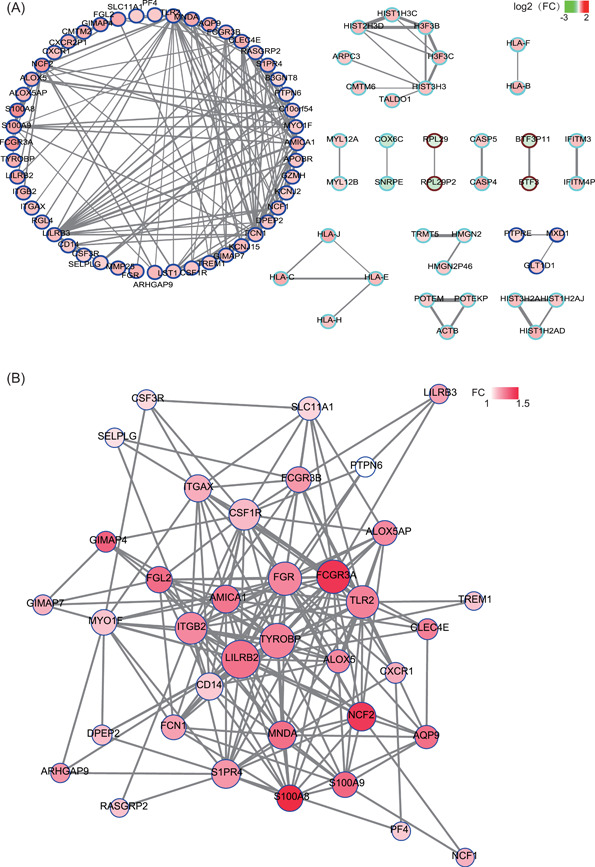
Identified hub genes from the blue module by the construction of co‐expression and protein–protein interactive (PPI) network. A, Co‐expression network by the enriched modules via WGCNA. Filled color represents the log 2 (fold change) of each gene, and border color represents the module that each gene belongs. B, PPI network of genes in the blue module. Filled color represents the fold change of each gene. WGCNA, weighted gene co‐expression network analysis

Based on the co‐expression network construction by WGCNA, genes in the blue module showed closer contact with each other than that in other modules (Figure 5A). Therefore, we used the blue module for further deep analysis. For each mRNA in a blue module, we evaluated the intramodular connectivity by co‐expression network and PPI network construction. Then, 13 hub genes with co‐expression network nodes ≥5 were screened (Figure 5B, Table 1). The GO analysis revealed that these genes were mainly involved in the biological process of immune‐related processes, such as the immune system process, immune response, and regulation of the immune system (Table S3). Totally, these results suggested that these genes could play pivotal roles in the pathogenesis of obstructive CAD.^21^

**Table 1:** List of hub‐genes in the blue module

Substance BXH	log 2 (FC)	*P*‐value	Module color	Co‐expression node	Nodes of PPI‐network notes
FGL2	0.2943	.0229	Blue	5	12
FCGR3B	0.2290	.0296	Blue	7	11
MYO1F	0.1736	.0224	Blue	8	11
MNDA	0.2896	.0158	Blue	8	14
CSF1R	0.1847	.0324	Blue	8	18
ALOX5	0.2524	.0078	Blue	9	8
S100A9	0.3014	.0051	Blue	10	11
NCF2	0.3801	.0013	Blue	11	15
S1PR4	0.2308	.00416	Blue	11	15
CLEC4E	0.2640	.0109	Blue	15	5
AMICA1	0.2795	.0060	Blue	17	14
FCN1	0.2165	.0192	Blue	20	10
TLR2	0.2602	.0052	Blue	22	20

Abbreviation: PPI, protein–protein interaction

### 3.4 Combination with risk factors and a cluster of four hub genes increases the diagnostic prediction for obstructive CAD

Due to the above‐mentioned observations, we further explored whether these hub genes in the blue module were associated with obstructive CAD by univariate logistic regression (ULR) analysis. We found that all the hub genes could be independent risk factors for obstructive CAD (Table 2).

**Table 2:** Univariate and stepwise multivariate logistic regression of hub genes and clinical traits

Hub genes/clinical traits	Univariate logistic regression			Stepwise multivariate logistic regression		
	OR	95% CI	*P*‐value	OR	95% CI	*P*‐value
Gender	2.81	1.38‐5.74	.004***	2.81	1.38‐5.74	.003***
BMI	0.97	0.93‐1.02	.278			
Diabetes	1.34	0.66‐2.75	.422			
Hyperlipid	3.06	1.44‐6.49	.004***	3.06	1.44‐6.49	.009**
Hypertension	1.02	0.35‐2.93	.976			
ALOX5	2.35	1.21‐4.55	.011*			
AMICA1	2.31	1.24‐4.29	.008**			
CLEC4E	2.15	1.16‐3.96	.014*			
CSF1R	2.12	1.05‐4.31	.037*			
FCGR3B	1.89	1.05‐3.41	.035*			
FGL2	1.71	1.06‐2.75	.028*			
MNDA	1.84	1.1‐3.07	.02*			
S100A9	2.22	1.23‐4	.008**			
TLR2	2.52	1.28‐4.95	.007**			
MYO1F	2.53	1.12‐5.75	.026*	2.53	1.12‐5.75	.103
NCF2	2.28	1.33‐3.9	.003***	2.28	1.33‐3.9	<.001***
S1PR4	3	1.37‐6.53	.006***	3	1.37‐6.53	.036*
FCN1	2.17	1.11‐4.21	.023*	2.17	1.11‐4.21	.01**

Abbreviations: CI, confidence interval; HR, hazard ratio; OR, odds ratio

*** *P* < .005.

** *P* < .01.

* *P* < .05.

**Figure 6:**
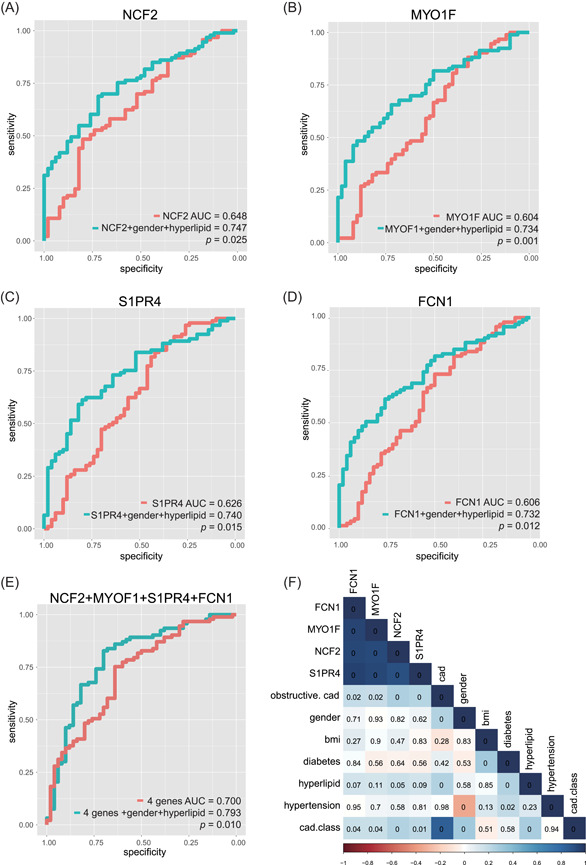
Receiver operating characteristic (ROC) curve analysis for the multivariate logistic regression (MLR) analyses and correlogram of genes and clinical traits. A‐D, Four hub genes (FCN1, MYO1F, NCF2, and S1PR4) with the lowest Akaike information criterion (AIC) value and two risk factors (sex and hyperlipid) were identified by MLR analysis in the data set GSE90074 (see also Table 2). E, ROC curve analysis of the four hub genes alone and the four hub genes combined with the two risk factors for the diagnosis of obstructive CAD. Area under the curve (AUC) indicates area and *P*‐value is shown under the ROC curve, respectively for A‐E. F, Correlogram of the correlation between the four hub genes and clinical traits by Pearson correlation coefficient procedure. The number in each box represents the *P*‐value of the Pearson correlation coefficients. The blue and red color gradient from dark to light in each box shows the degree of positive or negative correlations respectively in genes and clinical traits. CAD, coronary artery disease

Then, stepwise MLR analysis showed that four genes showed the lowest Akaike information criterion (AIC) value, which could remove other confounding factors and got the best result of data fitting. Four genes, including NCF2 (*P* = .025), MYO1F *(P* = .001), S1PR4 (*P* = .015), and FCN1 (*P* = .012), were outstanding in the current stepwise MLR analysis, which had a significant association with prognosis of obstructive CAD. Next, we explored whether these four genes (FCN1, MYO1F, NCF2, and S1PR4) were associated with obstructive CAD by performing ROC curve analysis in the same population. We found that the AUC was 0.606 for FCN1, 0.604 for MYO1F, 0.648 for NCF2, 0.626 for S1PR4, and 0.700 for the combination of these four genes (Figure 6A‐E). The mRNA levels of FCN1, MYO1F, NCF2, and S1PR4 were significantly higher in PBMCs from obstructive CAD patients than those from nonobstructive patients (*P* < .05, Figure S2). Taken together, these results demonstrate a high diagnostic accuracy of four‐gene signature as a novel biomarker for obstructive CAD.

As we know, CAD has many clinical risk factors, including age, sex, BMI, hypertension, hyperlipidemia, diabetes mellitus, and the degree of coronary artery obstruction. Then, further study was explored to analyze the relationship between the four genes and these CAD risk factors. Then, the results of ULR analyses showed that sex and hyperlipidemia were significantly associated with the diagnosis of CAD, but not with the other risk factors (Table 2). The AUC values for sex and hyperlipidemia are 0.626 and 0.618, respectively, with *P*‐values less than .05 (Figure S3).

As indicated above, the result of stepwise MLR analysis showed that the combination of clinical risk factors with the four genes with a lower AIC value indicated an association between NCF2, MYO1F, S1PR4, and FCN1 expression levels in PBMCs and male sex, and hyperlipidemia (Table 3). To determine whether these factors have an additive effect on the prediction values, these genes were analyzed by combining these two risk factors in the same group. The result showed that the diagnostic prediction was obviously increased, that is, AUC was 0.747 for NCF2, 0.734 for MYO1F, 0.740 for S1PR4, 0.737 for FCN1, and 0.793 for the combination of these four genes (Figure 6A‐E). Finally, to compare the diagnostic accuracy between NCF2, MYO1F, S1PR4, and FCN1 alone and the genes in combination with the risk factors, ROC curve analysis was performed again. The results indicated that a significant difference in prediction between the genes alone and the combined model (the genes plus risk factor groups) were found (*P *= .025, .001, .015, .012, and .010, respectively; Figure 6A‐E), which suggested that the diagnostic accuracy of the combined model enhanced the obstructive CAD discrimination.

**Table 3:** Stepwise multivariate logistic regression analyses for the 13 hub genes

Step	AIC
obstructivecad ~ gender+bmi+diabetes+hyperlipid+hypertension+ALOX5+AMICA1+CLEC4E+CSF1R+FCGR3B+FCN1+FGL2+MNDA+MYO1F+NCF2+S100A9+S1PR4+TLR2	180.58
obstructivecad ~ gender+bmi+diabetes+hyperlipid+hypertension+ALOX5+AMICA1+CLEC4E+CSF1R+FCGR3B+FCN1+FGL2+MYO1F+NCF2+S100A9+S1PR4+TLR2	178.6
obstructivecad ~ gender+bmi+diabetes+hyperlipid+ALOX5+AMICA1+CLEC4E+CSF1R+FCGR3B+FCN1+FGL2+MYO1F+NCF2+S100A9+S1PR4+TLR2	176.65
obstructivecad ~ gender+bmi+diabetes+hyperlipid+ALOX5+AMICA1+CLEC4E+CSF1R+FCN1+FGL2+MYO1F+NCF2+S100A9+S1PR4+TLR2	174.8
obstructivecad ~ gender+bmi+diabetes+hyperlipid+ALOX5+AMICA1+CLEC4E+FCN1+FGL2+MYO1F+NCF2+S100A9+S1PR4+TLR2	173.09
obstructivecad ~ gender+bmi+diabetes+hyperlipid+ALOX5+CLEC4E+FCN1+FGL2+MYO1F+NCF2+S100A9+S1PR4+TLR2	171.53
obstructivecad ~ gender+bmi+diabetes+hyperlipid+ALOX5+CLEC4E+FCN1+FGL2+MYO1F+NCF2+S100A9+S1PR4	169.81
obstructivecad ~ gender+bmi+diabetes+hyperlipid+ALOX5+CLEC4E+FCN1+FGL2+MYO1F+NCF2+S1PR4	168.03
obstructivecad ~ gender+bmi+hyperlipid+ALOX5+CLEC4E+FCN1+FGL2+MYO1F+NCF2+S1PR4	166.65
obstructivecad ~ gender+hyperlipid+ALOX5+CLEC4E+FCN1+FGL2+MYO1F+NCF2+S1PR4	165.46
obstructivecad ~ gender+hyperlipid+CLEC4E+FCN1+FGL2+MYO1F+NCF2+S1PR4	164.15
obstructivecad ~ gender+hyperlipid+FCN1+FGL2+MYO1F+NCF2+S1PR4	163.17
obstructivecad ~ gender+hyperlipid+FCN1+MYO1F+NCF2+S1PR4	162.36

Abbreviation: AIC, Akaike information criterion

Given the similar diagnostic values for the genes NCF2, MYO1F, S1PR4, and FCN1 in obstructive CAD, Spearman‐rank correlation was used to analyze the genes that correlated with the obstructive CAD and CAD severity. The results indicated that these four genes were correlated with one another significantly, but not correlated with the other risk factors (Figure 6F). All these data demonstrate that NCF2, MYO1F, S1PR4, and FCN1 in PBMC combination with sex and hyperlipidemia could be diagnostic biomarkers for obstructive CAD.

### 3.5 Four hub genes could also be good prediction biomarkers for ST‐segment elevation myocardial infarction

**Figure 7:**
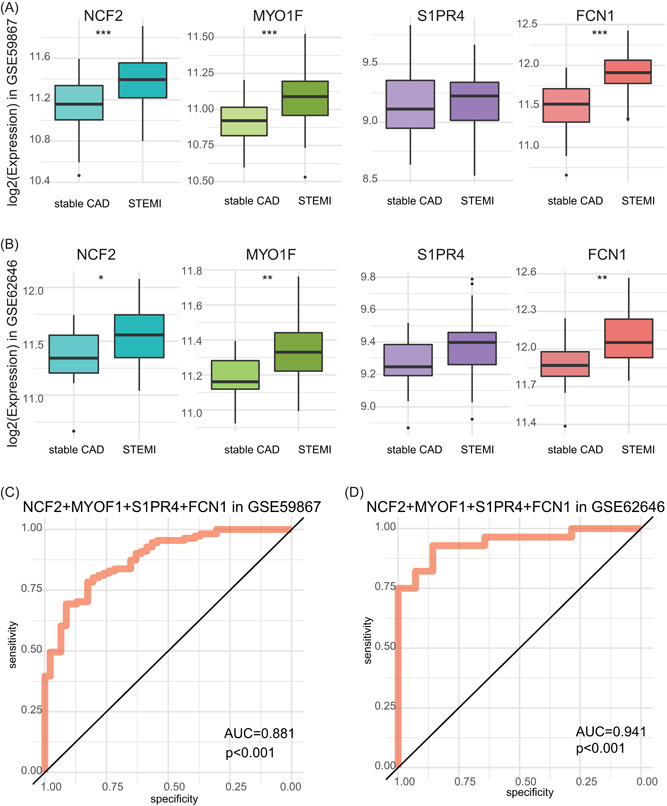
The mRNA expression levels and ROC curve analysis of four‐gene signature in PBMCs of stable CAD and STEMI patients in the data set GSE59867 or GSE62646. A,B, Relative expression levels of FCN1, MYO1F, NCF2, and S1PR4 in stable CAD and STEMI patients for the datasets of GSE59867 (A) and GSE62646 (B), respectively. Boxplots showing median, 25%–75% percentiles and range of log 2 (gene expression value). *P < .05; **P < .01; ***P < .001 (Student *t* test). C,D, ROC curve analysis of the four‐gene signature for the discrimination of stable CAD and STEMI patients for the datasets of GSE59867 (C) and GSE62646 (D), respectively. AUC indicates area and *P*‐value is shown under the ROC curve, respectively. AUC, area under the curve; CAD, coronary artery disease; mRNA, messenger RNA; PBMC, peripheral blood mononuclear cell; ROC, receiver operating characteristic; STEMI, ST‐segment elevation myocardial infarction

STEMI is the significant risk factor for obstructive CAD.^22^ Previous studies have reported that nearly 65% of patients presenting with STEMI had multivessel CAD, including obstructive CAD.^23^, ^24^ Therefore, we further validated whether these four genes could be also be used as a signature to predict the STEMI patients by using two more datasets (GSE62646 and GSE59867).^11^, ^12^ According to the annotation of both the datasets, the mRNA expression levels of FCN1, MYO1F, S1PR4, and NCF2 were increased in STEMI patients as compared to that in stable CAD patients without a history of myocardial infarction; although S1PR4 showed no statistical difference between the two groups (Figure 7A,B). ROC curve analysis also showed that the AUC of four hub genes in the datasets of GSE59867 and GSE62646 were 0.881 and 0.941, respectively (*P *< .001; Figure 7C,D). Interestingly, the AUC value was significantly reduced in S1PR4‐excluded GSE62646 (Figure S4), although the expression level of S1PR4 showed no significant changes between STEMI and stable CAD patients. Notably, these data demonstrate that this four‐gene signature could also act as an accurate biomarker for STEMI patients.

### 3.6 Functional enrichment analysis of genes correlated with obstructive CAD

**Figure 8:**
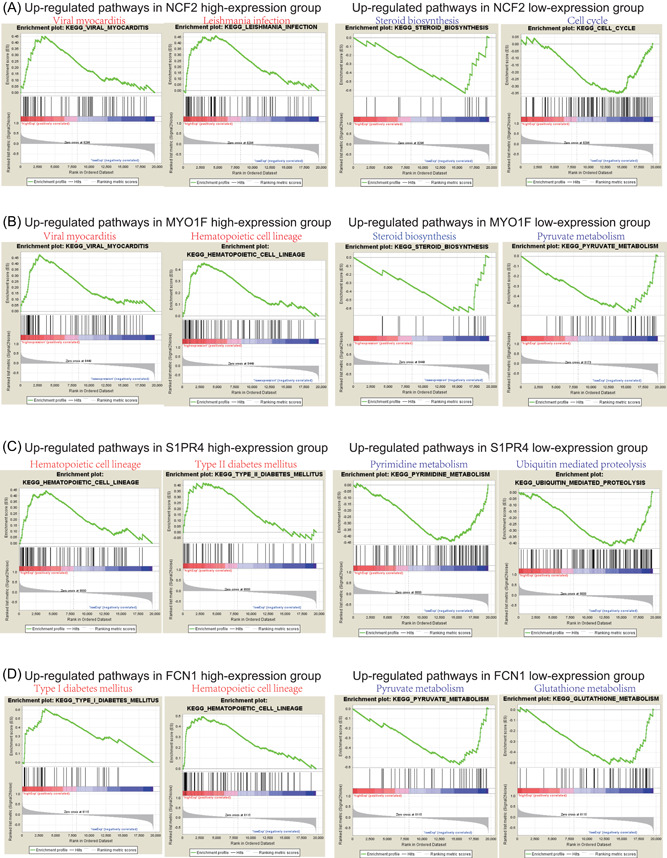
Gene set enrichment analysis of NCF2, MYO1F, FCN1, and S1PR4 in the PBMCs of obstructive CAD patients in the data set GSE90074. Top two enriched KEGG pathways in high‐expression (red) and low‐expression (blue) group of NCF2 (A), MYO1F (B), S1PR4 (C), and FCN1 (D), respectively. CAD, coronary artery disease; KEGG, Kyoto Encyclopedia of Genes and Genomes; PBMC, peripheral blood mononuclear cell

In the functional enrichment analysis of four‐finding genes, we divided the 93 obstructive CAD patients into two groups due to the expression levels of NCF2, MYO1F, S1PR4, or FCN1 (high‐expression group vs low‐expression group) and applied GSEA analysis to compare the different pathways between the two groups. Our finding showed that viral myocarditis,^25^ Leishmania infection,^26^ hematopoietic cell lineage,^27^ type I diabetes mellitus pathways, and type II diabetes mellitus pathways^28^ were enriched in patients with a higher expression of NCF2, MYO1F, S1PR4, and FCN1 in PBMCs. Also, these enriched pathways are previously reported as critical roles of these genes in the development of atherosclerosis and CADs. Whereas steroid biosynthesis, cell‐cycle pyruvate metabolism, glutathione metabolism, pyrimidine metabolism, and ubiquitin‐mediated proteolysis^29^, ^30^ were enriched in patients with a lower expression of NCF2, MYO1F, S1PR4, and FCN1 (Figure 8A‐D). All these data indicated that all these four hub genes might play similar and critical roles in the development and progression of obstructive CAD.

## 4 DISCUSSION

Decades of research have provided a deep understanding of the etiology of obstructive CAD, however, the predictive biomarkers, especially noninvasive biomarkers, for obstructive CAD diagnosis, are still limited.^5^, ^31^, ^32^ A comprehensive understanding of molecular mechanisms is primarily important for the diagnosis and treatment of obstructive CAD in clinical event. Great progress has been made in the diagnosis technique; but in many cases, especially, for obstructive CAD, it is still difficult to discriminate it from nonobstructive CAD.^33^, ^34^ In the current study, the data set GSE90074 was utilized for screening new potential noninvasive biomarkers for obstructive CAD. The datasets (GSE62646 and GSE59867), including stable CAD and STEMI patients, were used for further validations, an important risk factor for obstructive CAD.^22^, ^23^, ^24^ By doing this, the current study demonstrates that either gene, including NCF2, MYO1F, S1PR4, and FCN1, combined with the risk factors (including gender and hyperlipidemia), in PBMCs, was identified as a novel biomarker for obstructive CAD.

In this study, by deeply and systemically reanalyzing the GSE90074 data set, KEGG and GO analyses of total DEG demonstrate that during the development of obstructive CAD, immune system cells activated in the plaque, which is consistent with previous findings.^35^, ^36^ WGCNA of gene modules associated with clinical phenotypes identified three independent modules that are significantly associated with obstructive CAD and CAD classing. Furthermore, GO and KEGG enrichment analysis of DEG in these three modules revealed that the blue module was closely related to leukocyte activation and migration, which showed the similar phenotype with KEGG and GO analysis of total DEG. These findings indicated that DEG in the blue module could well present the phenotypic changes of PBMC in patients with obstructive CAD. Consistent with this notion, further, PPI network construction and ULR analysis unraveled 13 hub‐genes with co‐expression network nodes ≥5 in the blue module. Interestingly, ROC curve analysis and stepwise MLR analysis of blue module genes revealed that the combination of NCF2, MYO1F, S1PR4, and FCN1 could be used as a noninvasive biomarker for obstructive CAD. Subsequent analysis of the diagnostic value of these genes in obstructive CAD further confirmed that NCF2, MYO1F, S1PR4, and FCN1 together with risk factors, gender, and hyperlipidemia, could improve the diagnostic accuracy of distinguishing obstructive CAD from free of obstructive CAD.

Further validation of the diagnostic accuracy of NCF2, MYO1F, S1PR4, and FCN1 in STEMI patients showed that these four hub genes could also act as accurate biomarkers to discriminate STEMI patients from stable CAD patients. The expression levels of NCF2, MYO1F, S1PR4, and FCN1 were relatively higher in STEMI patients than in stable CAD patients. NCF2 is a component of the leukocyte NADPH oxidase complex that produces superoxide. Accumulating evidence has indicated that NCF2 plays critical roles in the development of autoimmune diseases, such as inflammatory bowel diseases, systemic lupus erythematosus,^37^, ^38^, ^39^ duodenitis, and Crohn's colitis.^40^, ^41^, ^42^ Moreover, a recent study indicated that NCF2 may play an important role in BP changes.^43^ MYO1F, a member of the myosin I family, is mainly expressed in bone marrow, spleen, appendix, and lymph nodes. MYO1F generally uses actin filaments as tracks by the energy from ATP hydrolysis.^44^ Interestingly, the specific function of MYO1F is still unclear so far. Recent studies indicate that it has a potential role in the pathogenesis of hearing loss,^45^, ^46^ and it is also critical for neutrophil migration in vivo or in 3‐D environments.^47^, ^48^ What is more, it plays an important role in the modulation of cell adhesion and motility in the immune system.^49^ FCN1, which encodes ficolin‐1, is involved in complement lectin pathway and elevated in patients with Takayasu arteritis^50^ or microscopic polyangiitis.^51^ So, the abnormal expression of FCN1 was a pathogenic factor and potential target of CADs.^52^ S1PR4 is mainly expressed in hematopoietic and lymphoid cells and plays a vital role in terminal megakaryocyte differentiation to platelets.^53^ Although the expression of S1PR4 showed no difference between STEMI and stable CAD patients, the lack of S1PR4 significantly reduced the accuracy of inspection. GSEA analysis also revealed that patients with obstructive CAD with higher expression levels of NCF2, MYO1F, S1PR4, and FCN1 in PBMC showed enriched pathways in viral myocarditis, Leishmania infection, type I diabetes mellitus, and hematopoietic cell lineage. These pathways are critical for the development of atherosclerosis and CADs.^25^, ^26^, ^27^ Since more than 65% of STEMI patients suffer from obstructive CAD,^23^, ^24^ it is presumable that this four‐gene signature could also be developed as a potential prognostic biomarker of obstructive CAD occurrence in STEMI patients. However, this warrants another separate study in the future.

In conclusion, a four‐gene signature (NCF2, MYO1F, S1PR4, and FCN1) could act as a noninvasive diagnostic biomarker for obstructive CAD. In combination with the risk factor, sex, and hyperlipidemia, it could improve the diagnostic accuracy of distinguishing obstructive CAD from free of obstructive CAD. Therefore, our study contributed a new potential noninvasive biomarker for obstructive CAD. Undoubtedly, future well‐accepted clinical studies with larger samples size, standardized protocols, and more homogenized populations would be needed to fully research the prognostics potential of this four‐gene signature in patients with obstructive CAD.

## CONFLICT OF INTERESTS

The authors declare that there are no conflict of interests.

## AUTHOR CONTRIBUTIONS

Xian‐Gang Mo designed experiments, analyzed data, and wrote the manuscript; Wei Liu, Yao Yang, Saber Imani, Shan Lu, Guorong Dan, Xuqiang Nie, Jun Yan, and Rixing Zhan designed experiments and analyzed data; Xiaohui Li revised the manuscript; Bingbo Chen and Yue Cai designed experiments, analyzed data, and edited the manuscript; Youcai Deng devised the concept, designed the research, supervised the study, and wrote the paper.

## Supporting information

Supporting information

Supporting information

Supporting information

Supporting information
