# Supplementary material for: NCF2, MYO1F, S1PR4, and FCN1 as potential noninvasive diagnostic biomarkers in patients with obstructive coronary artery: A weighted gene co‐expression network analysis
Source: J Cell Biochem. 2019 Jun 27;120(10):18219–35. doi: 10.1002/jcb.29128 (PMC6771964; doi:10.1002/jcb.29128)
Supplement: Supplementary file 1 — Supporting information [file JCB-120--s002.doc]

**NCF2, MYO1F, S1PR4 and FCN1 as a potential non-invasive diagnostic biomarker in patients with obstructive coronary artery: A weighted gene co-expression network analysis**

Xian-gang Mo, Wei Liu, Yao Yang, Saber Imani, Shan Lu, Guorong Dan, Xuqiang Nie, Jun Yan, Rixing Zhan, Xiaohui Li, Youcai Deng, Bingbo Chen, Yue Cai

**Supplemental Materials**

**
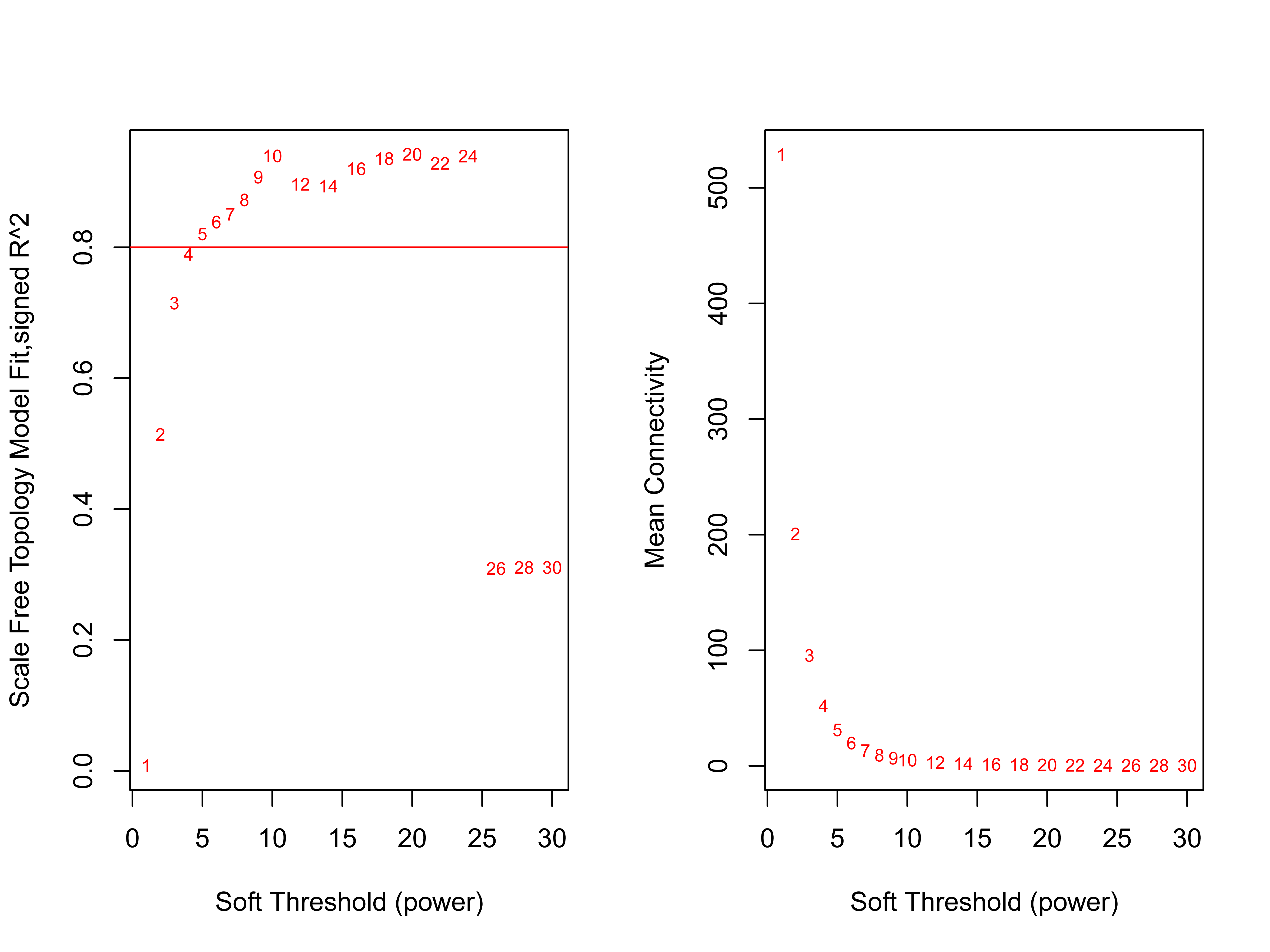
**

**Supplemental Figure 1** Analysis of the scale-free topology model fit index for various soft-thresholding powers (β) (Left panel) and analysis of the mean connectivity for various softthresholding powers (Right panel).

**
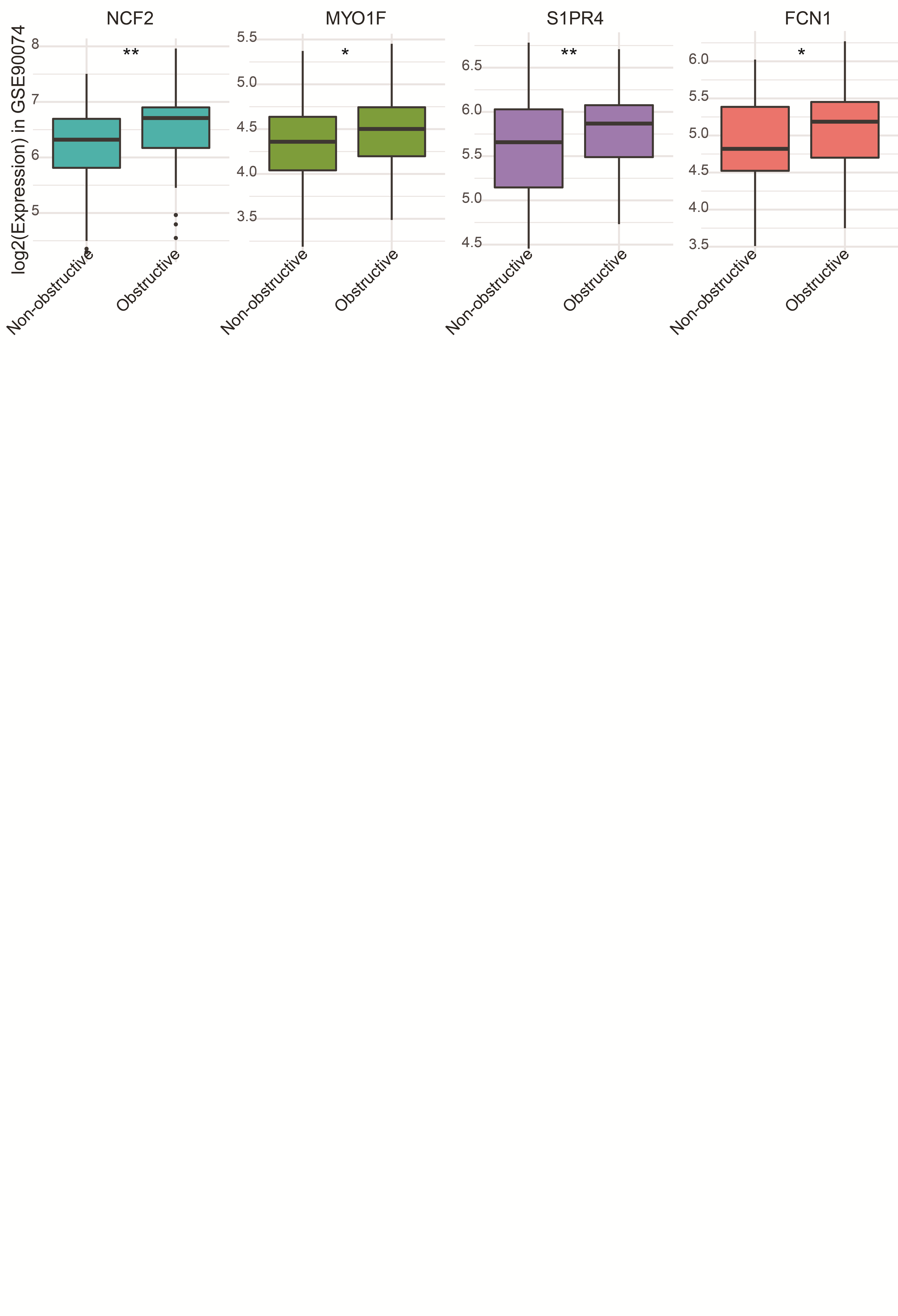
**

**Supplemental Figure2** The mRNA expression levels of 4-gene signature in PBMCs of non-obstructive and obstructive CAD patients in the dataset GSE90074. Boxplots showing median, 25%-75% percentiles and range of log2 (gene expression value). *, p < 0.05; **, p < 0.01; ***, p < 0.001 (Student's t-test).

**
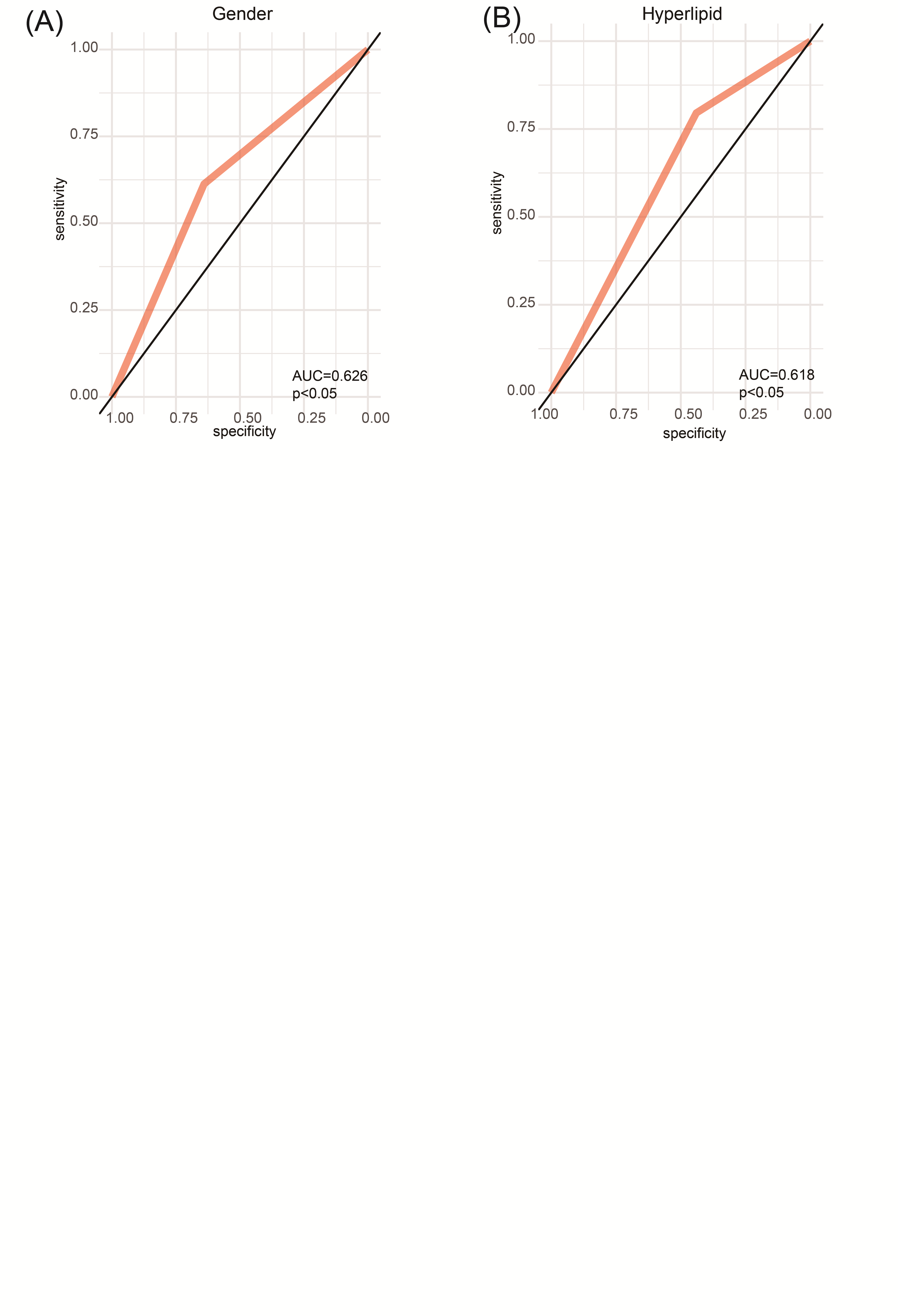
**

**Supplemental Figure 3** ROC curve analysis of gender (A) and hyperlipid (B) for the diagnosis of obstructive CAD in the dataset GSE90074. AUC indicates area and *p*-value is shown under the ROC curve, respectively.

**
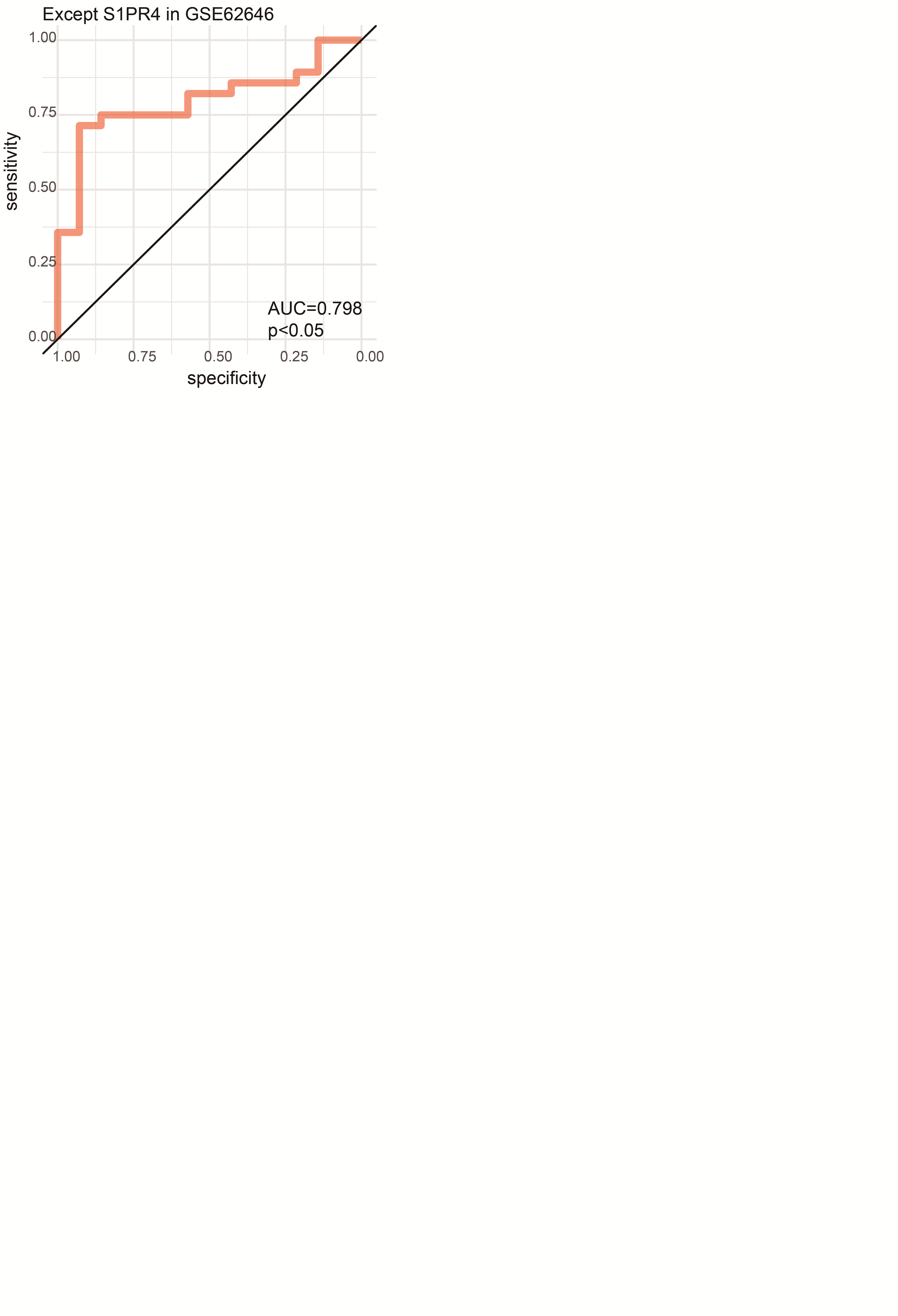
**

**Supplemental Figure 4** ROC curve analysis of the 4-gene signature, except S1PR4, for the discrimination of stable CAD and STEMI patients for the dataset GSE62646. AUC indicates area and *p*-value is shown under the ROC curve, respectively.
