# Supplementary material for: NCF2, MYO1F, S1PR4, and FCN1 as potential noninvasive diagnostic biomarkers in patients with obstructive coronary artery: A weighted gene co‐expression network analysis
Source: J Cell Biochem. 2019 Jun 27;120(10):18219–35. doi: 10.1002/jcb.29128 (PMC6771964; doi:10.1002/jcb.29128)
Supplement: Supplementary file 2 — Supporting information [file JCB-120--s004.doc]

| **Supplemental Table 1** Different expressed genes in CAD | | |
| --- | --- | --- |
| gene | logFC | P.Value |
| NBEA | -0.590024665 | 3.92E-05 |
| PHOSPHO2 | -0.369234571 | 8.47E-05 |
| SNURF | -0.339286818 | 0.000128593 |
| APBB1 | -0.340286483 | 0.000152226 |
| B2M | 0.215354721 | 0.000188161 |
| MEPCE | -0.129040126 | 0.000188793 |
| SCAND2 | -0.266773212 | 0.00020507 |
| PRKX | -0.321630863 | 0.000234963 |
| PRDM1 | 0.268573373 | 0.000268528 |
| ISG20 | 0.252022966 | 0.000302391 |
| WIPI1 | 0.296878918 | 0.00030939 |
| ARFGAP1 | -0.23051444 | 0.000324001 |
| URB1 | -0.285568135 | 0.000368225 |
| LCN15 | 0.261843849 | 0.000400769 |
| ESF1 | -0.29225802 | 0.000427843 |
| VPS52 | -0.198957711 | 0.000498393 |
| CLN8 | -0.233828112 | 0.000500642 |
| ZBTB39 | -0.520888804 | 0.000520474 |
| C1orf93 | -0.22512147 | 0.000531337 |
| ITGB2 | 0.251513738 | 0.00053602 |
| SRP14 | 0.162916636 | 0.000546734 |
| LOC728875 | 0.14982812 | 0.000553495 |
| EIF1AX | -0.246928819 | 0.000558038 |
| TYROBP | 0.261313097 | 0.000573784 |
| ABR | 0.160343503 | 0.000582998 |
| XYLT1 | -0.29580941 | 0.000589409 |
| GLIPR2 | 0.201016977 | 0.000595202 |
| SMC1A | -0.114020725 | 0.000616798 |
| PCDH7 | 0.168071019 | 0.000635622 |
| AIF1 | 0.21673292 | 0.000703221 |
| UBE2D4 | -0.163761877 | 0.000733531 |
| SCHIP1 | -0.423606369 | 0.000737168 |
| HLA-C | 0.280827476 | 0.000740045 |
| SYS1 | 0.14811618 | 0.000743943 |
| MEX3B | -0.278867768 | 0.00074623 |
| NBPF15 | 0.188179983 | 0.000750969 |
| FCGR3A | 0.390223474 | 0.000765658 |
| RRAGC | 0.138434122 | 0.000845654 |
| GBP2 | 0.222764426 | 0.000872115 |
| LINC00152 | 0.20570102 | 0.000882396 |
| FIS1 | -0.122636717 | 0.000891211 |
| C8orf46 | -0.261695959 | 0.000896354 |
| RG9MTD3 | -0.183718409 | 0.000910491 |
| ITGA11 | 0.335616097 | 0.000920433 |
| INTS4 | -0.204146972 | 0.00093033 |
| NEDD8 | 0.088857404 | 0.000933944 |
| RDH11 | -0.172824234 | 0.00095069 |
| DDX3Y | 1.283437623 | 0.000957015 |
| C2orf40 | -0.365736015 | 0.000972524 |
| XIST | -2.492702362 | 0.000973749 |
| PARP9 | 0.259747896 | 0.000990681 |
| PEMT | -0.198586186 | 0.000997281 |
| HLA-B | 0.25186331 | 0.001018149 |
| BTBD11 | -0.343911571 | 0.001018251 |
| CUX2 | -0.285686434 | 0.001025211 |
| MTG1 | -0.232599255 | 0.001048585 |
| ZFP36L2 | 0.193512725 | 0.001049772 |
| METTL19 | -0.141769206 | 0.001089436 |
| TCEA2 | -0.194612102 | 0.001095556 |
| LOC100509814 | -0.143371783 | 0.001105843 |
| VNN2 | 0.29022891 | 0.001107171 |
| ZNF671 | -0.157844529 | 0.001164773 |
| CYBASC3 | -0.195934356 | 0.001180948 |
| MYL5 | -0.23847578 | 0.001183045 |
| HLA-J | 0.29451586 | 0.001203708 |
| EDN3 | 0.156470456 | 0.001220354 |
| TNFAIP2 | 0.233277504 | 0.00123362 |
| HLA-F | 0.215188386 | 0.00126609 |
| ZNF789 | -0.263437166 | 0.001281762 |
| IRF9 | 0.20462462 | 0.001290456 |
| ZBED3 | -0.219248931 | 0.001297314 |
| LOC100128175 | -0.30868052 | 0.001303024 |
| NCF2 | 0.380059845 | 0.001307676 |
| SNHG10 | -0.237381861 | 0.001365887 |
| AVPR1A | -0.311218003 | 0.001381579 |
| TNRC6B | 0.137903374 | 0.001383234 |
| ZNF548 | -0.245393229 | 0.001385015 |
| HEATR1 | -0.184500599 | 0.00139061 |
| ZG16B | -0.392766817 | 0.001457806 |
| FYB | 0.177090733 | 0.001460076 |
| MEGF10 | 0.253018632 | 0.001466459 |
| KDM5D | 1.42621414 | 0.001575567 |
| FLJ40536 | -0.076925597 | 0.001595977 |
| FAM43A | -0.326880573 | 0.001608041 |
| HSD17B10 | -0.150470011 | 0.001622988 |
| HCN1 | 0.080558311 | 0.001628463 |
| TMPRSS11F | -0.145633565 | 0.001646789 |
| RPS4X | -0.183875827 | 0.001658908 |
| TAP1 | 0.221648867 | 0.001666457 |
| SHISA2 | 0.175832818 | 0.001677198 |
| LOC100288077 | -0.074844969 | 0.001685442 |
| ILVBL | -0.163660926 | 0.001687155 |
| P2RY13 | 0.379450564 | 0.001690245 |
| C19orf59 | 0.32105415 | 0.001691815 |
| GTF2H4 | -0.155573962 | 0.001700454 |
| SNED1 | -0.206112393 | 0.001702264 |
| COTL1 | 0.180190472 | 0.001739133 |
| CCNO | 0.172461006 | 0.001827271 |
| EIF1AY | 1.246290778 | 0.001827949 |
| RPS4Y2 | 1.706044091 | 0.001828556 |
| ADAM8 | 0.249951847 | 0.001849304 |
| PAPOLB | -0.135181353 | 0.001855462 |
| LAT2 | 0.162469174 | 0.001865833 |
| CD248 | -0.301536558 | 0.001886138 |
| TXLNG2P | 1.152545186 | 0.001909191 |
| EIF3L | -0.191968309 | 0.001923088 |
| LOC100507316 | -0.278042225 | 0.001926714 |
| RBM4B | -0.142353759 | 0.001927155 |
| LOC100289341 | -0.307062351 | 0.001955006 |
| CD53 | 0.253291929 | 0.001992363 |
| YPEL4 | -0.239061352 | 0.002002675 |
| KIN | 0.09789697 | 0.00200916 |
| DOCK8 | 0.214673056 | 0.002013621 |
| TGFBR2 | 0.174875337 | 0.002028796 |
| CD300LF | 0.299317815 | 0.002032841 |
| CEACAM21 | 0.391452061 | 0.002079278 |
| LOC648771 | -0.150234609 | 0.00210004 |
| SNRPN | -0.235542597 | 0.002138497 |
| EFHD2 | 0.145485847 | 0.002161661 |
| PLAG1 | -0.261196839 | 0.002192914 |
| GTF2E1 | 0.114332574 | 0.002198353 |
| LCP1 | 0.264449765 | 0.002216205 |
| C1orf52 | 0.158307914 | 0.002243152 |
| MAP2K5 | -0.274174404 | 0.002268649 |
| ATG16L2 | 0.218391202 | 0.002304227 |
| PAN3 | 0.190659584 | 0.002321224 |
| CYP2U1 | -0.189944647 | 0.002346731 |
| CA6 | -0.192672138 | 0.002353473 |
| ZNF444 | -0.102088712 | 0.002355373 |
| PKIB | -0.405672051 | 0.002364945 |
| FLYWCH1 | -0.196603907 | 0.002400671 |
| TMEM238 | -0.175317806 | 0.002409164 |
| ATG9B | -0.21827488 | 0.002462003 |
| HLA-E | 0.225256458 | 0.002469105 |
| FMNL3 | -0.204929473 | 0.002496918 |
| CARD17 | 0.200942427 | 0.002507447 |
| ERBB2IP | 0.147075218 | 0.00251745 |
| FCRLA | -0.53551426 | 0.002534618 |
| ASPG | 0.338885259 | 0.002566806 |
| GMFG | 0.276196457 | 0.002577364 |
| CPEB1 | -0.31146958 | 0.002578543 |
| PAIP2B | -0.23420879 | 0.002582655 |
| CRADD | 0.167420871 | 0.002586187 |
| SELL | 0.471717905 | 0.00259897 |
| ELL3 | -0.374589186 | 0.002617111 |
| LYSMD4 | -0.25350402 | 0.002617653 |
| RIMS3 | -0.275889776 | 0.002626221 |
| RPS4Y1 | 1.856142011 | 0.002640669 |
| STK4 | 0.152969949 | 0.002647294 |
| GIT2 | 0.141399667 | 0.002676155 |
| DIRAS3 | 0.313019563 | 0.002682629 |
| APBB1IP | 0.220307253 | 0.002693564 |
| CD40 | -0.313413942 | 0.002697114 |
| LINC00506 | -0.135314054 | 0.002705012 |
| USP15 | 0.223480829 | 0.002719785 |
| LILRB2 | 0.286083972 | 0.002728785 |
| OLIG3 | -0.430168179 | 0.00274151 |
| MRPS2 | -0.13144727 | 0.002773749 |
| RBP7 | 0.193540682 | 0.002807569 |
| VCPIP1 | 0.153944309 | 0.002821304 |
| ITPR2 | 0.234885537 | 0.002886921 |
| AKR7A3 | -0.12493414 | 0.002898872 |
| IFITM1 | 0.240370865 | 0.002899668 |
| WASH2P | 0.110932291 | 0.002900982 |
| TOR1AIP1 | 0.176319204 | 0.002938734 |
| TXNIP | 0.332658277 | 0.002944124 |
| NMI | 0.2355538 | 0.002983922 |
| PALMD | 0.251837866 | 0.002985756 |
| BDH1 | -0.13991884 | 0.002996433 |
| LOC100133131 | -0.377810041 | 0.002997764 |
| C14orf37 | 0.280565171 | 0.003025685 |
| BEND5 | -0.289634734 | 0.00305325 |
| TEKT4P2 | -0.571674886 | 0.003101858 |
| PCDHA5 | 0.389326774 | 0.003104748 |
| AP4S1 | -0.243631069 | 0.003117413 |
| WSCD1 | 0.254005246 | 0.003131545 |
| SCRN2 | -0.120836502 | 0.003137674 |
| LRRC56 | -0.232826702 | 0.003141219 |
| MIEN1 | 0.067959986 | 0.003148465 |
| OR4K1 | 0.237280808 | 0.003161057 |
| ALDH5A1 | -0.215612641 | 0.003175897 |
| COL22A1 | 0.146460654 | 0.003179352 |
| S100A8 | 0.418439473 | 0.003206276 |
| IL2RB | 0.245796896 | 0.003248781 |
| NIPAL3 | -0.210568063 | 0.00327688 |
| CCR4 | -0.320483578 | 0.003278179 |
| PCBP3 | -0.358127861 | 0.003284812 |
| FAM180A | 0.370997035 | 0.003287205 |
| NELF | -0.163461309 | 0.003307679 |
| FAM27A | 0.368943726 | 0.0033102 |
| CASP4 | 0.257463169 | 0.003321053 |
| SIDT1 | -0.331823722 | 0.003321711 |
| MAZ | -0.172181383 | 0.003337181 |
| BCL7A | -0.271323029 | 0.003357175 |
| SHISA5 | 0.192829541 | 0.003396161 |
| GNL1 | -0.147140606 | 0.003411458 |
| FGR | 0.254246585 | 0.003414955 |
| GMIP | 0.143455453 | 0.003417888 |
| PSMC4 | -0.100376138 | 0.003439297 |
| NDUFB11 | -0.144765088 | 0.003468404 |
| COL4A3 | -0.269258058 | 0.003472978 |
| LILRB3 | 0.218064073 | 0.003478417 |
| DEDD | 0.136059288 | 0.0035065 |
| ZNF439 | -0.279995331 | 0.003563613 |
| SLC26A1 | -0.16006825 | 0.003566065 |
| CAPZA1 | 0.246098959 | 0.003570601 |
| ZNF581 | -0.170692352 | 0.003590687 |
| C6orf62 | 0.180335138 | 0.003603952 |
| PPP1R10 | 0.139866778 | 0.003604954 |
| LOC100653227 | -0.250204753 | 0.003612055 |
| CASP1 | 0.17729577 | 0.003612681 |
| BRD9 | -0.140894873 | 0.003635425 |
| FPR1 | 0.274616946 | 0.003642391 |
| MMS19 | -0.113286709 | 0.00367414 |
| QRSL1 | -0.192303727 | 0.003699032 |
| DTX3L | 0.227892407 | 0.003754832 |
| CEBPZ | -0.123301735 | 0.00376144 |
| FAM183B | -0.300047341 | 0.003765686 |
| JDP2 | 0.195868255 | 0.003776553 |
| ARHGAP9 | 0.233019606 | 0.003779173 |
| RAB8B | 0.160689294 | 0.003824053 |
| NTN1 | 0.193745491 | 0.003833982 |
| ZDBF2 | -0.489539321 | 0.003838725 |
| LOC100287628 | -0.239760297 | 0.003850237 |
| TCTN1 | -0.239959824 | 0.003850427 |
| WIPF1 | 0.159336203 | 0.003888918 |
| FAM104A | 0.083837561 | 0.003904301 |
| CENPH | -0.287587016 | 0.003916598 |
| CD300A | 0.142089477 | 0.003920884 |
| PMEPA1 | -0.196449533 | 0.004016304 |
| CCL4 | 0.245530602 | 0.00408827 |
| PTPRK | -0.378340094 | 0.00410846 |
| SLA | 0.225505558 | 0.004115859 |
| PIGU | -0.175784268 | 0.004126479 |
| KRCC1 | 0.129097943 | 0.004152908 |
| WDR89 | -0.230399683 | 0.004154131 |
| S1PR4 | 0.230848352 | 0.004159813 |
| AP2A1 | -0.221613319 | 0.004175323 |
| MCF2L-AS1 | -0.284332152 | 0.004183503 |
| LOC100506122 | -0.077011294 | 0.004196999 |
| FBXW9 | -0.142950196 | 0.004209886 |
| GIMAP5 | 0.283429777 | 0.004218612 |
| TSPEAR | -0.37433368 | 0.004265025 |
| TUBGCP4 | -0.267950295 | 0.004267492 |
| SP100 | 0.140029186 | 0.004309693 |
| SLC18A2 | -0.33426703 | 0.004326504 |
| CD274 | 0.415681333 | 0.004339254 |
| RCSD1 | 0.172545526 | 0.00434715 |
| NBAS | -0.119097003 | 0.004357686 |
| RBL1 | -0.231472574 | 0.004383817 |
| SYT1 | 0.275656413 | 0.004388685 |
| DCAF4L2 | 0.421923897 | 0.004432522 |
| PDXP | 0.12777025 | 0.004441803 |
| CTSS | 0.167402367 | 0.004446645 |
| FBXO15 | -0.441940761 | 0.00445914 |
| LOC155060 | -0.227255435 | 0.004466266 |
| PLBD1 | 0.263918207 | 0.00448589 |
| CSF3 | 0.070188911 | 0.004487495 |
| LRRC55 | 0.113797054 | 0.004497013 |
| SRGN | 0.315689193 | 0.004521609 |
| GCHFR | -0.10182716 | 0.004528658 |
| CIR1 | 0.171925948 | 0.004566592 |
| SDR39U1 | -0.182153388 | 0.004601367 |
| DUT | -0.191506 | 0.004646303 |
| MMP25 | 0.214093922 | 0.004668844 |
| MTA3 | -0.13664144 | 0.004683188 |
| TNFSF10 | 0.192911927 | 0.004685008 |
| LOC157562 | -0.21305085 | 0.00469112 |
| ANKRD32 | 0.168491396 | 0.004692186 |
| CD93 | 0.285285825 | 0.004719997 |
| NKRF | -0.163630032 | 0.004732573 |
| ZNF250 | -0.154827437 | 0.00473875 |
| ERGIC3 | -0.146390694 | 0.004754221 |
| CRTAP | -0.215832867 | 0.004754915 |
| LOC641518 | -0.586672964 | 0.004783513 |
| DYNLT1 | 0.216743616 | 0.004786417 |
| 9-Mar | -0.151361309 | 0.004801179 |
| IDUA | -0.230583492 | 0.004847486 |
| NFKBIA | 0.326256511 | 0.004853104 |
| RMI1 | 0.187806618 | 0.00485345 |
| KBTBD5 | 0.165764231 | 0.004880026 |
| PPM1J | -0.321718679 | 0.004919102 |
| BTNL8 | 0.599780071 | 0.004924315 |
| SCGB3A1 | -0.683714136 | 0.004927476 |
| TRIM21 | 0.168329734 | 0.004932162 |
| ZFY | 0.44319182 | 0.004977322 |
| PPP1R18 | 0.137775408 | 0.004989912 |
| ICAM4 | -0.358539385 | 0.005000492 |
| SNRPA | -0.107558791 | 0.005003678 |
| AGPHD1 | -0.390284065 | 0.005005685 |
| ZNF788 | 0.149879287 | 0.005024802 |
| NOL6 | -0.147036008 | 0.00504709 |
| SAMM50 | -0.115522724 | 0.00505983 |
| VTI1A | 0.095494431 | 0.005080407 |
| S100A9 | 0.301430542 | 0.005102694 |
| CDK4 | -0.18396133 | 0.005107547 |
| RP9 | -0.125284386 | 0.005113341 |
| C6orf47 | 0.112256555 | 0.00516382 |
| KAT2A | -0.17408112 | 0.005174292 |
| TLR2 | 0.260152248 | 0.005174424 |
| IFT57 | -0.203588601 | 0.005206111 |
| ZDHHC1 | 0.316104082 | 0.005207359 |
| ATP1A3 | -0.147382714 | 0.005224307 |
| ELOVL4 | -0.276772611 | 0.005249851 |
| KCNE1L | 0.21919766 | 0.005262575 |
| TMED4 | -0.161630867 | 0.005290314 |
| PPBP | 0.374815042 | 0.005300449 |
| ADAT2 | -0.200231632 | 0.005308585 |
| CCDC144NL | 0.485857039 | 0.00531201 |
| SPRED1 | -0.352222923 | 0.005331153 |
| DAPK2 | 0.215165308 | 0.005343786 |
| DSCR3 | 0.110255604 | 0.005362763 |
| PIAS2 | 0.262396886 | 0.005394523 |
| OSTF1 | 0.170778843 | 0.005454258 |
| SLC23A2 | -0.230605814 | 0.005510609 |
| CD80 | -0.112940319 | 0.005512171 |
| CCDC105 | 0.08703144 | 0.005584306 |
| FLJ32255 | 0.191137409 | 0.005587437 |
| SENP5 | 0.074711905 | 0.005589423 |
| ARRDC5 | -0.464879769 | 0.005609137 |
| FAM58A | -0.147608126 | 0.005620464 |
| ANKMY1 | -0.24682795 | 0.005641362 |
| YWHAB | 0.125629903 | 0.00564334 |
| CA5B | -0.158506802 | 0.005659945 |
| DMAP1 | -0.121133784 | 0.005661552 |
| ATP5D | -0.143081019 | 0.005683427 |
| C16orf5 | -0.172418364 | 0.005726388 |
| ALDH16A1 | -0.163115334 | 0.005731823 |
| C9orf16 | -0.141939745 | 0.005786217 |
| RPL18A | -0.133415326 | 0.005804127 |
| LINC00461 | 0.243095409 | 0.00581083 |
| CDKAL1 | -0.152903657 | 0.005818347 |
| ASB9 | -0.319509063 | 0.00583144 |
| GEMIN4 | -0.131550311 | 0.005841718 |
| ITLN1 | -0.555812744 | 0.005882656 |
| ABHD14A | -0.166456082 | 0.005885989 |
| SQRDL | 0.203426047 | 0.005898565 |
| CYP3A4 | 0.248641743 | 0.005902973 |
| RAD23A | -0.132308052 | 0.005916915 |
| HIGD2A | -0.119484211 | 0.005920565 |
| CLYBL | -0.195023399 | 0.005925441 |
| DNAJC24 | -0.307906545 | 0.005937158 |
| ANKRD30BL | 0.208037635 | 0.005954525 |
| PLXDC2 | 0.286407229 | 0.005955253 |
| AMICA1 | 0.279457588 | 0.005964466 |
| TRAPPC6A | -0.12829737 | 0.005976704 |
| CYTIP | 0.158535744 | 0.005983815 |
| LOC728012 | -0.104326753 | 0.005997051 |
| YIF1A | -0.118336868 | 0.005997857 |
| F13A1 | 0.2857377 | 0.006002488 |
| CD1C | -0.296193248 | 0.006018943 |
| ZBTB2 | 0.120107665 | 0.00602313 |
| TRIM22 | 0.21418279 | 0.006029641 |
| DDB1 | -0.118016082 | 0.006049817 |
| DNAJA3 | -0.15390493 | 0.006059875 |
| NOB1 | -0.146004853 | 0.006076632 |
| GAPT | 0.4335984 | 0.0060825 |
| CYP4F2 | 0.278228425 | 0.006098842 |
| PHF11 | 0.170912744 | 0.006104552 |
| STK31 | -0.168094759 | 0.006126852 |
| GADD45B | 0.186016979 | 0.006147376 |
| PIM1 | 0.147105161 | 0.006164794 |
| CD1B | -0.362391617 | 0.006181153 |
| DIP2C | -0.372000891 | 0.006182744 |
| LRRN2 | 0.218492802 | 0.006186413 |
| CALM2 | 0.239241772 | 0.006213617 |
| NTNG2 | 0.181513323 | 0.006240837 |
| RAB8A | 0.212411012 | 0.00624791 |
| GRWD1 | -0.09391006 | 0.00625196 |
| LOC100507493 | -0.247950145 | 0.006259389 |
| LOC643802 | 0.293169816 | 0.006303498 |
| OVCA2 | -0.094995602 | 0.006319651 |
| RIC3 | -0.402827701 | 0.006327434 |
| TOP3B | -0.254208326 | 0.006399049 |
| GSR | 0.177024086 | 0.006405402 |
| WAC | 0.113906366 | 0.006413912 |
| CFP | 0.242844928 | 0.006418266 |
| RPL12 | -0.107162404 | 0.00643201 |
| ZNF500 | -0.280545403 | 0.006441002 |
| SNHG11 | -0.141679441 | 0.006461236 |
| KPNB1 | 0.247623714 | 0.006538364 |
| C11orf80 | -0.255627516 | 0.006549183 |
| TOLLIP | 0.177436999 | 0.006558168 |
| UPK2 | 0.203920685 | 0.006592439 |
| CYTH4 | 0.226361644 | 0.006623404 |
| LOC100507487 | -0.36689301 | 0.006649591 |
| COL19A1 | -0.398753614 | 0.006665174 |
| PDE4DIP | -0.179659994 | 0.006677561 |
| GPLD1 | -0.208481684 | 0.006679509 |
| RASGRP2 | 0.176680966 | 0.006690006 |
| PPIE | -0.181977183 | 0.006694871 |
| RYR1 | -0.285200765 | 0.00669649 |
| ZCCHC6 | 0.203227494 | 0.006697799 |
| LOC100506667 | 0.079698183 | 0.006705923 |
| ZNF544 | -0.130665868 | 0.00670821 |
| DRG2 | -0.123811524 | 0.006727384 |
| CCZ1 | 0.128682753 | 0.006751529 |
| HNRNPK | 0.131479527 | 0.006768933 |
| PPP3R1 | 0.244224983 | 0.006797206 |
| MCM7 | -0.122094666 | 0.006799526 |
| SNX9 | -0.228813409 | 0.006804692 |
| ZMYM6 | -0.176134957 | 0.006812711 |
| GPER | 0.505956497 | 0.006821894 |
| EXOC1 | 0.096176182 | 0.006843859 |
| UNC119B | -0.121873996 | 0.006880005 |
| FAM20B | -0.165656558 | 0.006892691 |
| PRKCB | 0.106670592 | 0.00692568 |
| GIMAP6 | 0.18193205 | 0.00693399 |
| ZNF232 | -0.154845245 | 0.006996687 |
| BSCL2 | -0.165080982 | 0.007007527 |
| PSMB9 | 0.204899258 | 0.007038891 |
| EVI2A | 0.180338803 | 0.007060357 |
| STMN3 | -0.167500561 | 0.007068189 |
| ISL2 | -0.488493165 | 0.007073889 |
| DOK3 | 0.223238872 | 0.007090508 |
| NENF | -0.155711048 | 0.00709897 |
| OSCAR | 0.201118354 | 0.007134042 |
| COX6A1 | 0.098639674 | 0.00714541 |
| PPT1 | 0.276279784 | 0.007158552 |
| RSF1 | 0.095177424 | 0.007175121 |
| LPAL2 | 0.097416535 | 0.007178841 |
| PYGO2 | -0.073462814 | 0.007189916 |
| ARPC5 | 0.234869646 | 0.007242309 |
| ADAR | 0.174928722 | 0.007242441 |
| PLEKHH3 | 0.146028527 | 0.00727769 |
| PRR13 | 0.176476835 | 0.007282757 |
| PCDHB14 | 0.218207115 | 0.007309885 |
| PPP2R3C | 0.173606951 | 0.007324123 |
| DCXR | -0.151570885 | 0.007352665 |
| POLG | -0.108111773 | 0.00737824 |
| HSPA6 | 0.185538393 | 0.007390996 |
| ZNHIT2 | -0.186733274 | 0.007415954 |
| GEMIN8 | -0.166298417 | 0.007423765 |
| GPR27 | 0.431224468 | 0.007426498 |
| PSMD7 | 0.084082155 | 0.007426799 |
| CDH1 | -0.265983841 | 0.007458853 |
| KDM5A | 0.110639367 | 0.007477935 |
| HMGN2P46 | 0.176526412 | 0.007519181 |
| OGFOD1 | -0.146155297 | 0.007530782 |
| ZNF582 | -0.408193713 | 0.00757427 |
| FAM154A | -0.100475178 | 0.007590662 |
| WBP11 | -0.12868662 | 0.00760189 |
| RAB7B | -0.17516605 | 0.007642216 |
| DKFZP434C153 | 0.142986959 | 0.007676982 |
| ALOX5 | 0.252370309 | 0.007757941 |
| KLF2 | 0.167028168 | 0.00786712 |
| NUFIP1 | -0.210474379 | 0.007874387 |
| DBI | 0.114005586 | 0.007882156 |
| LOC100128398 | -0.215034831 | 0.007898296 |
| PRRC2B | -0.135486854 | 0.007899342 |
| CD27 | 0.17555562 | 0.007901216 |
| ELK3 | -0.236648951 | 0.00790266 |
| MAN1B1 | -0.176768498 | 0.007931539 |
| LAYN | -0.187756454 | 0.007978718 |
| RAP2C | 0.181873981 | 0.007980769 |
| ATP6V0E2 | -0.345296536 | 0.007993619 |
| TRPM4 | -0.384829946 | 0.007999812 |
| DGCR6L | -0.095103483 | 0.008017091 |
| MYH10 | 0.153584736 | 0.00803257 |
| LOC338799 | -0.183934199 | 0.008057509 |
| ELP2 | -0.215923439 | 0.008060389 |
| TFRC | -0.193316612 | 0.008062811 |
| CHUK | 0.248159135 | 0.008077396 |
| SNRPE | -0.1875284 | 0.008103803 |
| GLT1D1 | 0.185949076 | 0.008104012 |
| S100A12 | 0.259238872 | 0.008107638 |
| NDUFAF1 | 0.118243612 | 0.00818654 |
| ALG10B | -0.222796196 | 0.008225014 |
| EPHA3 | 0.146524372 | 0.008228923 |
| CELF2 | 0.145831798 | 0.008253072 |
| LCP2 | 0.129211926 | 0.008271977 |
| BLK | -0.354125887 | 0.008288118 |
| VEZF1 | 0.080752676 | 0.008291878 |
| PPP1R3F | -0.143832093 | 0.008356642 |
| C1orf213 | -0.177224394 | 0.00835988 |
| GABBR1 | -0.273684461 | 0.008367958 |
| HSPB8 | 0.244315799 | 0.008396578 |
| RALGAPA1 | -0.124974785 | 0.008427834 |
| RPL29P2 | -0.179865836 | 0.00845184 |
| GET4 | -0.072277031 | 0.008454162 |
| KRT20 | 0.238993012 | 0.008531699 |
| C6orf211 | 0.100740573 | 0.008532162 |
| MLL5 | 0.092036082 | 0.008537562 |
| HARS | -0.099125985 | 0.008602858 |
| ZNF773 | -0.152304845 | 0.008622786 |
| HCLS1 | 0.219897584 | 0.008625488 |
| DOCK11 | 0.115181357 | 0.008665091 |
| HINT3 | 0.174915277 | 0.008668526 |
| STX11 | 0.251869089 | 0.008673315 |
| FLI1 | 0.127644325 | 0.008685448 |
| LOC100505683 | -0.286580479 | 0.008706114 |
| LOC100131170 | 0.288178333 | 0.008708392 |
| AP2A2 | -0.089875189 | 0.008727431 |
| B3GNT4 | -0.170084264 | 0.008749478 |
| ATF7IP | -0.140380768 | 0.008766 |
| SELPLG | 0.14577773 | 0.008801681 |
| ACOT2 | -0.155649212 | 0.008807858 |
| ANKRD54 | -0.148973789 | 0.008818579 |
| POLR1E | -0.229329902 | 0.008883203 |
| TNFRSF9 | 0.419186632 | 0.008886738 |
| UQCRB | -0.171409649 | 0.008915315 |
| ZNF3 | -0.121150355 | 0.008941994 |
| CDC14C | -0.291041789 | 0.008957274 |
| TCEA3 | -0.290810456 | 0.008963033 |
| TOR1A | 0.170141262 | 0.008972797 |
| KLHL36 | -0.093175192 | 0.008985354 |
| ARCN1 | 0.118062643 | 0.00900072 |
| APOBR | 0.180228718 | 0.009006285 |
| RPL36 | -0.147446802 | 0.009023792 |
| BLNK | -0.411309536 | 0.009025035 |
| CH25H | 0.253368277 | 0.009033113 |
| IRF3 | -0.116629459 | 0.009094848 |
| MAP9 | -0.291260885 | 0.009103547 |
| RBM12 | -0.098353051 | 0.009108509 |
| LST1 | 0.19498133 | 0.009117794 |
| ADAMTS4 | -0.090742893 | 0.009159631 |
| ENAM | -0.077016237 | 0.009186859 |
| C19orf53 | -0.112659961 | 0.009208874 |
| GTF3C5 | -0.080213628 | 0.009213782 |
| TNIP3 | -0.224623818 | 0.009216159 |
| SENP8 | -0.293167777 | 0.009216923 |
| DLX4 | -0.225636961 | 0.00921892 |
| HEBP2 | 0.233592182 | 0.009242447 |
| CCDC124 | -0.092821205 | 0.009253064 |
| ACAD9 | -0.15148345 | 0.009261728 |
| SLC16A6 | 0.187016131 | 0.009283321 |
| DDX31 | -0.203332113 | 0.009326475 |
| NFYC | 0.13710427 | 0.009347096 |
| CXCR4 | 0.220266797 | 0.009357834 |
| C9orf128 | -0.31844252 | 0.0093633 |
| RBM19 | -0.110614994 | 0.009373934 |
| EMX1 | 0.162969802 | 0.00937537 |
| PP2672 | 0.06788714 | 0.00943628 |
| AGPAT4 | -0.170106343 | 0.0094441 |
| SEC22B | 0.191332449 | 0.00946312 |
| TCP10L2 | -0.271636359 | 0.009475911 |
| PYCRL | -0.108195633 | 0.00947932 |
| WDR92 | -0.111081629 | 0.009485249 |
| RGPD5 | -0.109047524 | 0.009504695 |
| SAT1 | 0.268372596 | 0.009558704 |
| IQCK | -0.236642588 | 0.009573915 |
| TSPAN13 | -0.341252627 | 0.009579915 |
| LOC728855 | 0.122623864 | 0.009591993 |
| RNF122 | 0.170872394 | 0.009609838 |
| TELO2 | -0.115377687 | 0.009641698 |
| ANAPC11 | 0.085761662 | 0.009645375 |
| ALOX5AP | 0.250821629 | 0.009667473 |
| FAM86A | -0.233943391 | 0.009674446 |
| RALYL | 0.147666832 | 0.009717695 |
| PRMT7 | -0.160795399 | 0.009718149 |
| CKAP5 | -0.101399938 | 0.009731901 |
| RRP1 | -0.107534589 | 0.00975961 |
| DDX60L | 0.270025217 | 0.009760092 |
| SDHB | 0.096230326 | 0.009761574 |
| GIMAP4 | 0.31087344 | 0.009764971 |
| CBX3 | 0.129264991 | 0.009789743 |
| ZNF16 | -0.261263712 | 0.009801095 |
| ALKBH7 | -0.10250628 | 0.009821172 |
| ACSS3 | 0.25557158 | 0.009825399 |
| CDCA7L | -0.184959342 | 0.009853728 |
| KIDINS220 | 0.13050455 | 0.009870221 |
| BAG3 | -0.159484206 | 0.009909875 |
| RPL3 | -0.105785135 | 0.009911859 |
| SORBS3 | -0.196705216 | 0.009913599 |
| FAM129C | -0.405264514 | 0.009933739 |
| CHIC2 | 0.224388482 | 0.009956711 |
| DCTPP1 | -0.115402442 | 0.00997 |
| EFHB | -0.201377211 | 0.009988297 |
| PNPLA4 | -0.23947473 | 0.010052958 |
| CYP7A1 | -0.240985952 | 0.010129937 |
| C11orf2 | -0.111574016 | 0.010242216 |
| KCNH8 | -0.623593856 | 0.010246124 |
| MPEG1 | 0.297771063 | 0.010269602 |
| GLRX | 0.179827302 | 0.010271707 |
| SLX4 | -0.109108381 | 0.010300508 |
| PRKCA | -0.151693071 | 0.010307447 |
| FKBP7 | -0.22153164 | 0.01031384 |
| C15orf44 | -0.136214146 | 0.010318256 |
| SSTR3 | 0.319207735 | 0.010324996 |
| C1GALT1C1 | 0.115097186 | 0.010344417 |
| LOC100507507 | 0.138046202 | 0.010382196 |
| MFSD7 | 0.164826769 | 0.010403585 |
| KIAA0240 | 0.107922879 | 0.010431477 |
| GAS5 | -0.25418706 | 0.010482636 |
| KIAA0247 | 0.13701163 | 0.010492175 |
| DHX36 | -0.086295807 | 0.010518932 |
| FBLN5 | -0.286098605 | 0.010522616 |
| GATA5 | 0.233998274 | 0.010526757 |
| IGLON5 | 0.292304085 | 0.010554615 |
| POTEG | 0.179276745 | 0.010624701 |
| FOXP2 | 0.079930618 | 0.010635589 |
| STK10 | 0.106967444 | 0.010646205 |
| BTF3 | -0.116775802 | 0.010647705 |
| USP47 | -0.111071025 | 0.010664646 |
| KCTD8 | 0.171230659 | 0.010689797 |
| RNF144B | 0.24678784 | 0.010691591 |
| ACTB | 0.239038859 | 0.01073995 |
| GTF2IRD2B | -0.125200604 | 0.01076921 |
| CBLC | -0.059593716 | 0.010787937 |
| DMRTC1 | -0.646864201 | 0.010827822 |
| GNA12 | -0.124147005 | 0.010867109 |
| MCRS1 | -0.081926393 | 0.010881712 |
| CLEC4E | 0.263951951 | 0.010898627 |
| FAM100B | 0.221415805 | 0.010916795 |
| GATAD2A | 0.100157307 | 0.010922218 |
| C9orf46 | 0.126218444 | 0.010922431 |
| ARHGEF4 | 0.132153434 | 0.010986327 |
| C8orf60 | 0.156689511 | 0.01100077 |
| PSMD4 | 0.106999076 | 0.011067052 |
| PLCG2 | 0.126648786 | 0.011137989 |
| HTR7P1 | -0.212947098 | 0.011162711 |
| DPM3 | -0.140258289 | 0.011173128 |
| BCS1L | -0.141317403 | 0.011203648 |
| MOB2 | -0.09696595 | 0.011217143 |
| ASXL1 | -0.092268662 | 0.01122886 |
| LZTS2 | -0.111872131 | 0.01124652 |
| LYRM1 | 0.201056357 | 0.011254634 |
| LOC100131096 | -0.216394066 | 0.01126986 |
| CDS2 | 0.186006493 | 0.011307145 |
| ACOT7 | -0.134651396 | 0.011329627 |
| IK | 0.11421703 | 0.011348757 |
| UBA3 | 0.171008293 | 0.011370958 |
| UPF1 | 0.160951091 | 0.011412789 |
| TMBIM6 | 0.145517713 | 0.011439136 |
| RGL4 | 0.208329955 | 0.011469399 |
| NUAK2 | 0.190995418 | 0.01150115 |
| C20orf24 | 0.196451083 | 0.01151346 |
| SIRPD | 0.32325555 | 0.011527336 |
| ITGA9 | -0.184130701 | 0.011562537 |
| TPPP3 | -0.470254451 | 0.011572964 |
| DHRSX | 0.153805041 | 0.011581516 |
| MAPK11 | -0.118211126 | 0.011606369 |
| BNC1 | 0.237462868 | 0.011688578 |
| TINF2 | 0.078491194 | 0.011750898 |
| C17orf108 | -0.251370989 | 0.011755591 |
| CHML | -0.183480388 | 0.011767804 |
| STEAP4 | 0.312915957 | 0.011771567 |
| MPI | -0.112509095 | 0.011822215 |
| CEACAM4 | 0.21060757 | 0.011855806 |
| MRPL30 | -0.126554167 | 0.011868043 |
| ZCRB1 | -0.154321237 | 0.011879322 |
| GOLT1B | -0.197479609 | 0.011882972 |
| N4BP3 | -0.125027794 | 0.011884498 |
| ZNF280C | -0.198467481 | 0.011902384 |
| PIGL | -0.201979227 | 0.011920337 |
| CYTH1 | 0.132676009 | 0.011925986 |
| CAPS | -0.17138771 | 0.011927696 |
| EAPP | 0.095181713 | 0.011933787 |
| ND5 | -0.139720416 | 0.011936471 |
| ZNF804B | -0.050898848 | 0.011977947 |
| DDT | -0.116783405 | 0.012003851 |
| IL17RA | 0.199594557 | 0.012023693 |
| PRMT1 | -0.097512939 | 0.012116037 |
| BST1 | 0.180473936 | 0.012117483 |
| POLR2I | -0.110372395 | 0.012118909 |
| KIAA1191 | -0.118351925 | 0.012122707 |
| STK33 | -0.271929841 | 0.012150372 |
| NFE2 | 0.270170482 | 0.012186713 |
| DNASE1L3 | -0.483785929 | 0.01219514 |
| NAT9 | -0.12199422 | 0.012233758 |
| C20orf106 | 0.192864807 | 0.012238503 |
| CLEC4D | 0.357478967 | 0.012239338 |
| B3GAT3 | -0.123096414 | 0.012239931 |
| MSTO1 | -0.217145259 | 0.012263148 |
| GRASP | -0.200340655 | 0.012271179 |
| IRF5 | 0.221387719 | 0.012297678 |
| ABI1 | 0.13121804 | 0.012316544 |
| POP5 | -0.135499326 | 0.012341948 |
| FCAR | 0.287322347 | 0.01235064 |
| PCMTD1 | 0.179626777 | 0.012360728 |
| IRAK3 | 0.295109544 | 0.012371691 |
| OTUD4 | -0.149055232 | 0.012384388 |
| ANKRD5 | -0.266612616 | 0.01241004 |
| TDRD10 | 0.262445218 | 0.012421919 |
| DGCR2 | 0.128884332 | 0.012448691 |
| P4HTM | -0.155639875 | 0.01245043 |
| PAWR | -0.255238518 | 0.012474957 |
| DENND5A | 0.162051502 | 0.012513997 |
| EVI2B | 0.256799087 | 0.01258289 |
| KIAA0430 | 0.107181609 | 0.012591075 |
| RPA3 | -0.13642003 | 0.012607137 |
| PAPD7 | -0.13685853 | 0.012627178 |
| LARS2 | -0.083768308 | 0.012640043 |
| LOC100287590 | -0.193244707 | 0.01265325 |
| LY6H | 0.136924149 | 0.012681868 |
| CLEC16A | -0.157176541 | 0.012700108 |
| ATG4B | -0.088165856 | 0.012726776 |
| DDI2 | -0.175747053 | 0.01273078 |
| HTATIP2 | 0.120416699 | 0.012741775 |
| TUT1 | -0.244037482 | 0.01275334 |
| FAM160B1 | 0.17862527 | 0.012754393 |
| TALDO1 | 0.221069975 | 0.012775393 |
| UBE2I | -0.088920128 | 0.012814602 |
| DENND1C | 0.14676696 | 0.012850642 |
| ZNF382 | -0.375152907 | 0.012857111 |
| UG0898H09 | 0.096402806 | 0.01292775 |
| AGPAT4-IT1 | -0.204480754 | 0.012943003 |
| FTL | 0.214743128 | 0.01294311 |
| ZNF219 | -0.124163658 | 0.012947084 |
| XPNPEP1 | -0.115172272 | 0.012987552 |
| SIRT3 | -0.097638902 | 0.012990228 |
| AP3S1 | 0.130341867 | 0.013004899 |
| MLKL | 0.12965618 | 0.013006287 |
| NPFFR1 | 0.140982036 | 0.0130125 |
| HECA | 0.09278936 | 0.013029809 |
| ACSM1 | -0.349358201 | 0.013072329 |
| KCNJ3 | 0.243409608 | 0.013088357 |
| GNLY | 0.293619745 | 0.013098914 |
| VARS2 | -0.0984782 | 0.013122593 |
| PRKCD | 0.133374786 | 0.01319529 |
| GSTM5 | 0.172857024 | 0.013195937 |
| GSK3A | 0.0786931 | 0.013203381 |
| NDRG2 | -0.161591601 | 0.013213652 |
| MCF2L | -0.127285785 | 0.013215533 |
| FAM41C | 0.117959397 | 0.01323172 |
| AKAP17A | 0.127943405 | 0.013269677 |
| CD22 | -0.434249052 | 0.013300332 |
| GOLGA6L10 | -0.152522528 | 0.013310003 |
| ZC3HAV1 | 0.186691269 | 0.013325532 |
| RPS3 | -0.152554636 | 0.013330975 |
| BTF3P11 | -0.148238291 | 0.013367887 |
| DEXI | -0.121544681 | 0.013420722 |
| CSTF3 | -0.140404786 | 0.013438537 |
| PHLDB3 | -0.192088551 | 0.013450163 |
| MRPS11 | -0.094002225 | 0.013481273 |
| ATRIP | -0.135356003 | 0.013502164 |
| GZMK | 0.258202697 | 0.013515818 |
| FAM86B2 | -0.120840454 | 0.013522705 |
| LYN | 0.187531092 | 0.013525084 |
| PLEK | 0.239778374 | 0.013530925 |
| ZNF580 | -0.127464653 | 0.013532927 |
| EIF2D | -0.120750449 | 0.013561931 |
| ACMSD | 0.13039359 | 0.013579394 |
| CDK2AP2 | -0.115911609 | 0.013591872 |
| DSCR6 | 0.201451632 | 0.01360332 |
| FZD3 | -0.263294678 | 0.013620636 |
| MMP12 | 0.170292874 | 0.013653804 |
| NCAM1 | 0.127537141 | 0.013701594 |
| TLR6 | 0.238122791 | 0.013703308 |
| TMEM102 | -0.148311161 | 0.013707569 |
| NQO1 | 0.34890364 | 0.013736127 |
| OBFC2A | 0.186252369 | 0.013742903 |
| EPHX2 | -0.295539147 | 0.01375685 |
| PLSCR1 | 0.331920654 | 0.01382704 |
| USP32P2 | 0.228539847 | 0.013836348 |
| PSD3 | -0.171325978 | 0.01384978 |
| SUSD3 | -0.209663008 | 0.013869133 |
| CLIC1 | 0.181486043 | 0.013869767 |
| RGS19 | 0.166399174 | 0.013872559 |
| IYD | -0.23348415 | 0.01390423 |
| OST4 | -0.169330787 | 0.013930964 |
| B3GALT4 | 0.089997503 | 0.014026428 |
| HPRT1 | -0.111287992 | 0.014189129 |
| RPL10 | -0.101932203 | 0.014237334 |
| FLRT2 | 0.191643536 | 0.014263635 |
| RPS6KB1 | -0.087324963 | 0.01426393 |
| SERGEF | -0.120648981 | 0.014342335 |
| SNX6 | 0.125001728 | 0.014351129 |
| C14orf80 | -0.127788997 | 0.014361659 |
| PALM2-AKAP2 | 0.121188579 | 0.014372851 |
| PINX1 | -0.160734137 | 0.014397269 |
| ZNF84 | -0.16603656 | 0.014399088 |
| LOC100132319 | 0.246344856 | 0.014411249 |
| CREG1 | 0.148832405 | 0.014411529 |
| C10orf95 | -0.268749299 | 0.014434399 |
| REEP1 | -0.338099696 | 0.014436721 |
| FOXL1 | 0.285135861 | 0.014445922 |
| C11orf45 | -0.256529546 | 0.014452531 |
| BCORP1 | 0.133370159 | 0.014474882 |
| MRPL54 | -0.115063243 | 0.014512812 |
| CLRN1 | 0.074309189 | 0.014527876 |
| SF3B14 | 0.110330589 | 0.014538033 |
| DPPA4 | -0.283771647 | 0.014571376 |
| PDDC1 | -0.137010531 | 0.014599403 |
| DOLK | -0.084425404 | 0.014638572 |
| TFPT | -0.076126008 | 0.014641021 |
| LOC442421 | -0.099837638 | 0.014661933 |
| C20orf103 | -0.304240035 | 0.014669302 |
| CCNB1IP1 | -0.135879491 | 0.014681212 |
| GAN | -0.272760248 | 0.014686849 |
| PI4KA | -0.117100328 | 0.01468697 |
| PECAM1 | 0.146228485 | 0.014705002 |
| AKR7L | -0.117108202 | 0.014739722 |
| SMTN | -0.219840943 | 0.014743873 |
| HLA-H | 0.18681533 | 0.014743944 |
| LOC284933 | 0.20078867 | 0.014749106 |
| GJA10 | 0.049093275 | 0.014778613 |
| YDJC | -0.067119773 | 0.014782331 |
| LOC643355 | -0.330287098 | 0.014791264 |
| MXD1 | 0.201949957 | 0.014799785 |
| GINS1 | 0.242927153 | 0.014802485 |
| TOP1MT | -0.217175096 | 0.014807372 |
| ZNF215 | 0.190980265 | 0.014813285 |
| IER2 | 0.212595355 | 0.014818172 |
| ZC3H4 | 0.096691124 | 0.01483133 |
| FMNL1 | 0.124627732 | 0.014834404 |
| AP2M1 | -0.127905492 | 0.014846171 |
| TTC37 | -0.191867316 | 0.014847607 |
| VSIG1 | -0.509462163 | 0.014855645 |
| ZNF681 | -0.190126019 | 0.014914073 |
| TMEM154 | 0.268438901 | 0.014971256 |
| PCDHGB1 | -0.095136879 | 0.01497446 |
| LRRC25 | 0.271065006 | 0.01497592 |
| PMS2L2 | -0.099270837 | 0.015026497 |
| LOC100287216 | -0.132536259 | 0.015034404 |
| FGF21 | 0.225869219 | 0.015038583 |
| LSM5 | -0.157123758 | 0.015108664 |
| CCDC144A | 0.233298094 | 0.01515565 |
| SPRY3 | 0.157333793 | 0.01516516 |
| CUL3 | -0.277828626 | 0.015233106 |
| CRYM | -0.362375946 | 0.015241261 |
| ZNF546 | -0.192718351 | 0.015241847 |
| COL9A3 | 0.438663143 | 0.015245603 |
| LOC100128893 | 0.274216456 | 0.015298272 |
| NKAP | 0.088650686 | 0.015325319 |
| NECAB1 | 0.220367823 | 0.015358798 |
| TCF4 | -0.150449856 | 0.015425573 |
| EGLN3 | -0.271903416 | 0.015433439 |
| NBPF3 | 0.130886337 | 0.015445299 |
| TBC1D4 | -0.260006291 | 0.015450039 |
| BCKDHB | -0.195664589 | 0.015466154 |
| FAM86EP | -0.164143268 | 0.015472921 |
| QARS | -0.135556368 | 0.01547823 |
| HIST1H2AD | 0.26496569 | 0.015494 |
| TOMM6 | -0.160518328 | 0.015517113 |
| DDX59 | 0.174741515 | 0.015525562 |
| CCNDBP1 | 0.168045652 | 0.015533322 |
| WFDC10B | 0.088085961 | 0.015576239 |
| CEP63 | 0.178672711 | 0.015581232 |
| ZNF167 | 0.200074294 | 0.015598956 |
| ARHGAP10 | -0.159342264 | 0.015652841 |
| CD40LG | -0.449591751 | 0.015657104 |
| HIST2H3D | 0.223223812 | 0.0156723 |
| SH3KBP1 | 0.089013455 | 0.015677931 |
| ATP9B | -0.192608091 | 0.01569985 |
| SYNE2 | 0.171760526 | 0.015732785 |
| GIMAP8 | 0.167358757 | 0.015738891 |
| ZMPSTE24 | 0.142023444 | 0.015776456 |
| MNDA | 0.289550867 | 0.015782963 |
| TSPAN3 | -0.170281672 | 0.015787742 |
| RUVBL2 | -0.083137101 | 0.015809032 |
| PTBP1 | -0.198090132 | 0.015868023 |
| INSM1 | 0.228759235 | 0.015869929 |
| CHST9-AS1 | -0.116307232 | 0.015883746 |
| CCL3 | 0.144776923 | 0.015890119 |
| HERPUD1 | 0.117983359 | 0.015896688 |
| ERV3-2 | 0.283943304 | 0.015904906 |
| ZNF285 | -0.42497041 | 0.015927998 |
| UBE2A | 0.06319896 | 0.015954776 |
| C21orf2 | -0.210532658 | 0.015982683 |
| HIST1H3C | 0.225908187 | 0.01601822 |
| COQ4 | -0.126031963 | 0.016054171 |
| RPL39 | -0.124063626 | 0.016122009 |
| FBXL15 | -0.100891048 | 0.016133433 |
| NOL4 | 0.05156784 | 0.016165288 |
| UPF2 | 0.157505866 | 0.016177343 |
| TMEM160 | -0.11370471 | 0.01618807 |
| FCGRT | 0.141388322 | 0.016198909 |
| FAM176A | 0.223638815 | 0.016216405 |
| LBR | 0.245871542 | 0.016289747 |
| CHGA | 0.208528032 | 0.016337772 |
| LOC100507280 | -0.307077228 | 0.016339637 |
| TRIM34 | 0.132617377 | 0.016355957 |
| PTPRE | 0.116858104 | 0.016371235 |
| KRT6C | 0.131874094 | 0.016376153 |
| SNPH | -0.227800922 | 0.016398284 |
| THOP1 | -0.094779485 | 0.01640829 |
| XPO4 | -0.185583308 | 0.016436513 |
| CA5BP1 | -0.332818355 | 0.01644775 |
| GLTSCR2 | -0.137551352 | 0.016477416 |
| PSMB1 | 0.068990321 | 0.016489016 |
| APOO | -0.116079748 | 0.016491276 |
| LGR5 | 0.205800741 | 0.016494828 |
| CXorf21 | 0.285811426 | 0.016516078 |
| ENG | -0.157439494 | 0.016530326 |
| SUDS3 | 0.114677191 | 0.016611595 |
| C8orf38 | -0.229790828 | 0.016623927 |
| DPH2 | -0.116002996 | 0.01665415 |
| EPHB6 | -0.159710336 | 0.016657798 |
| CXorf23 | 0.155571697 | 0.01668724 |
| ELMO1 | 0.208397837 | 0.016689169 |
| ING1 | 0.129374384 | 0.016770544 |
| SCARA5 | -0.165352948 | 0.016787793 |
| GABPB2 | -0.153922278 | 0.01681114 |
| ATP4A | -0.197217194 | 0.016822709 |
| BEX5 | -0.276348136 | 0.016840461 |
| SPANXA2-OT1 | -0.346095098 | 0.016842803 |
| ZC3H12D | -0.26894729 | 0.016846888 |
| LYZL1 | 0.361906211 | 0.016862583 |
| FCRL1 | -0.405987677 | 0.016899658 |
| PNMT | 0.399330525 | 0.016916931 |
| CMBL | 0.289166496 | 0.016977181 |
| RNF38 | 0.167505285 | 0.01697948 |
| DUS1L | -0.100607563 | 0.01698639 |
| FAM49A | 0.167207535 | 0.017012121 |
| UGT1A8 | 0.092102823 | 0.017016817 |
| PIAS1 | 0.128830162 | 0.017019664 |
| NCF4 | 0.180785648 | 0.01702263 |
| ITPR1 | -0.108898368 | 0.017022809 |
| FMR1 | 0.101849855 | 0.017058998 |
| CCDC166 | -0.375426036 | 0.017067603 |
| FAM40A | -0.071362242 | 0.017106752 |
| PRPSAP1 | -0.105597468 | 0.017153989 |
| EPHB2 | 0.356869447 | 0.017176279 |
| PPIL1 | -0.131520325 | 0.017202726 |
| IL1R2 | 0.300592503 | 0.017227279 |
| KLF8 | -0.368275102 | 0.017293089 |
| SLC15A1 | 0.093599963 | 0.017301366 |
| C16orf54 | 0.278736819 | 0.017337441 |
| TBC1D25 | -0.304275311 | 0.017367608 |
| CAT | 0.212540009 | 0.01739511 |
| CHTF8 | -0.105571373 | 0.017431561 |
| C1orf192 | -0.043703906 | 0.017549037 |
| CASP5 | 0.166927342 | 0.017576322 |
| NANOS3 | 0.274817296 | 0.017628547 |
| LOC650226 | 0.254237524 | 0.017637943 |
| ITGAX | 0.201316123 | 0.017649756 |
| TCEAL5 | -0.143359939 | 0.017651549 |
| AKIRIN2-AS1 | 0.200509127 | 0.017661062 |
| LOC100652953 | -0.160479527 | 0.017678931 |
| FOXG1 | 0.129746701 | 0.017743983 |
| DBT | -0.178494817 | 0.017778479 |
| ATPAF1 | -0.1724221 | 0.017792179 |
| FAM86FP | -0.14264057 | 0.01779754 |
| L3MBTL2 | -0.17136138 | 0.017800246 |
| LTB | 0.191075654 | 0.017813517 |
| NACAP1 | -0.132552301 | 0.017829769 |
| HMGN2 | 0.131704572 | 0.017835449 |
| NECAP1 | 0.166485014 | 0.017837783 |
| ZNF709 | -0.17523209 | 0.017876186 |
| LYRM7 | -0.168170057 | 0.017881781 |
| SAC3D1 | -0.083422463 | 0.017896234 |
| CYP46A1 | 0.236504486 | 0.017896651 |
| ZNF92 | -0.102466835 | 0.017901878 |
| CHD1L | -0.119825726 | 0.018079423 |
| DDX46 | -0.071342436 | 0.018096945 |
| METTL8 | -0.195831498 | 0.018121733 |
| LOC727820 | 0.13285631 | 0.018162799 |
| ARRB2 | 0.132965258 | 0.018166641 |
| TLR4 | 0.18197456 | 0.018186966 |
| ARMC5 | -0.089166082 | 0.018187191 |
| TRPC1 | -0.303826967 | 0.018192517 |
| EFTUD1 | 0.16966342 | 0.018219958 |
| TMPRSS11D | -0.104106246 | 0.01823476 |
| SNAP29 | -0.149420443 | 0.018242894 |
| C10orf54 | 0.170669468 | 0.018250169 |
| OR7G3 | 0.07376948 | 0.018262272 |
| KRT19 | 0.111492409 | 0.018285555 |
| C14orf45 | -0.342971755 | 0.018298525 |
| FAHD2A | -0.087511981 | 0.018347483 |
| MYBPC3 | 0.186895603 | 0.018359279 |
| FAM113B | -0.213753688 | 0.018376409 |
| GOLGA7 | 0.135046045 | 0.018419536 |
| ELK4 | -0.218115122 | 0.018426865 |
| DNAJA1 | 0.114282553 | 0.018457592 |
| C6orf26 | -0.132718783 | 0.018458338 |
| CD180 | -0.566028262 | 0.018481233 |
| NF2 | -0.137901431 | 0.018493949 |
| MDH1B | 0.141311327 | 0.018506195 |
| KCNK16 | -0.14141591 | 0.018506395 |
| PRDX2 | -0.110019718 | 0.018540168 |
| ACTR2 | 0.147031997 | 0.018550559 |
| FBXO6 | 0.156830146 | 0.018557654 |
| ADAMTS19 | 0.21664229 | 0.018620021 |
| HSDL1 | -0.235760769 | 0.018645494 |
| GNG13 | 0.404753302 | 0.018688529 |
| GLIPR1 | 0.095267182 | 0.018736436 |
| SAMD9L | 0.245858802 | 0.018753818 |
| MGC3771 | -0.360240546 | 0.018762891 |
| NDUFC1 | -0.151254393 | 0.018768266 |
| PASK | -0.250323246 | 0.018778672 |
| KANK4 | 0.192504949 | 0.018813659 |
| SP3 | 0.10189448 | 0.018846204 |
| REPIN1 | -0.085640202 | 0.018880301 |
| RFESD | -0.214704417 | 0.018909779 |
| RGS2 | 0.289307264 | 0.018955196 |
| WDR74 | -0.080433993 | 0.018957703 |
| PYCARD | 0.103522256 | 0.0189639 |
| SLC7A7 | 0.182945553 | 0.01896501 |
| ZDHHC23 | -0.243224191 | 0.018982451 |
| RPL13 | -0.102056362 | 0.018997396 |
| KRBA1 | -0.119834799 | 0.019038103 |
| USF1 | 0.324206464 | 0.019045375 |
| LOC100506262 | -0.387180624 | 0.019047571 |
| ERCC2 | -0.093846983 | 0.019049176 |
| SNX11 | 0.168272482 | 0.019053108 |
| PTPN6 | 0.117453991 | 0.019054019 |
| ZYG11A | 0.194478042 | 0.019059438 |
| STAP1 | -0.585371317 | 0.019064726 |
| STAG3 | -0.133491628 | 0.019101038 |
| FP588 | -0.258782488 | 0.019113166 |
| SH3GL1 | 0.108512835 | 0.019155937 |
| FCN1 | 0.216502669 | 0.019164154 |
| GLRA2 | 0.06313732 | 0.019169121 |
| POTEE | 0.159299708 | 0.019171385 |
| HRH1 | -0.188146134 | 0.019182891 |
| ZBTB5 | -0.121277767 | 0.019213729 |
| LOC440104 | -0.152455976 | 0.019226285 |
| PSKH1 | -0.072989794 | 0.019227641 |
| C17orf64 | 0.150973946 | 0.01926315 |
| C21orf91 | 0.100563069 | 0.019267203 |
| FAM82B | -0.078635367 | 0.019267995 |
| BEND3 | -0.147549218 | 0.019288413 |
| MS4A7 | 0.242059946 | 0.019326804 |
| NCF1 | 0.196344153 | 0.019374704 |
| C19orf38 | 0.147548433 | 0.01937843 |
| EIF2AK3 | -0.256484174 | 0.01937972 |
| SMAD1 | -0.196282537 | 0.019394962 |
| NR3C2 | -0.430291659 | 0.019472884 |
| CRELD1 | -0.128679078 | 0.019494805 |
| NR1I2 | 0.250713637 | 0.019516438 |
| GAMT | -0.096686001 | 0.019539354 |
| LOC285141 | 0.251074027 | 0.01957661 |
| ORC1 | -0.278484935 | 0.019582731 |
| PPEF1 | -0.349497073 | 0.019585074 |
| RAB24 | 0.14519377 | 0.019600964 |
| MIER1 | 0.116579494 | 0.01961349 |
| C16orf62 | -0.149615471 | 0.019621495 |
| CEP44 | -0.171273386 | 0.019639329 |
| TRDMT1 | -0.164637545 | 0.019641221 |
| MEF2C | -0.181210817 | 0.019664742 |
| ATF7 | 0.119784564 | 0.019684515 |
| H2AFY | 0.172725977 | 0.019701402 |
| PLEKHA3 | 0.142727076 | 0.019712084 |
| TMEM163 | -0.229249875 | 0.019722657 |
| RAC2 | 0.107482597 | 0.019734955 |
| CCDC66 | -0.116710408 | 0.019735642 |
| LOC100129794 | 0.248677637 | 0.019750517 |
| NOM1 | -0.221049065 | 0.019771711 |
| TNKS2 | 0.171535457 | 0.019772101 |
| RALB | 0.221847888 | 0.01978513 |
| MAB21L1 | -0.106128658 | 0.019789728 |
| ASB16 | 0.153230795 | 0.01979013 |
| UPK1B | 0.058504438 | 0.019823638 |
| RPS10P7 | -0.084151985 | 0.019864202 |
| UGT2B10 | 0.103653364 | 0.01995663 |
| LOC100127972 | -0.297366551 | 0.019959651 |
| C18orf25 | 0.158526843 | 0.019972738 |
| AGBL5 | -0.127333771 | 0.019974809 |
| ND3 | -0.15614152 | 0.020039403 |
| SPRR3 | 0.291396882 | 0.020106644 |
| RFFL | 0.131178056 | 0.02011442 |
| PNLIPRP2 | 0.22815251 | 0.020132467 |
| PRPF40A | 0.087247169 | 0.020135852 |
| DFNB59 | -0.30333896 | 0.020141636 |
| FIZ1 | -0.157084369 | 0.020174171 |
| APOF | -0.183865803 | 0.020227959 |
| RRM1 | -0.216340751 | 0.020242566 |
| DSG1 | -0.037444585 | 0.020247384 |
| LOC100506651 | -0.166001998 | 0.020254362 |
| LOC100128775 | 0.073596515 | 0.020261279 |
| CATSPER3 | -0.295735474 | 0.020262187 |
| LONRF2 | 0.209559356 | 0.020305461 |
| MEI1 | 0.095507123 | 0.020383638 |
| LOC100507311 | 0.237673586 | 0.020385719 |
| NR3C1 | 0.074068596 | 0.020395265 |
| MTERFD3 | 0.162888086 | 0.020412789 |
| DDX50 | -0.15386363 | 0.020427217 |
| SNX20 | 0.15792858 | 0.020476707 |
| TBC1D30 | -0.087106699 | 0.020477759 |
| GZMH | 0.188929133 | 0.020487704 |
| PRAM1 | 0.141561058 | 0.020497893 |
| BIN1 | -0.162642846 | 0.020558576 |
| KIAA1671 | 0.153105492 | 0.020563921 |
| MOK | -0.239945206 | 0.020566794 |
| MAN1C1 | -0.269106231 | 0.020569186 |
| LOC100505622 | -0.501008455 | 0.020591021 |
| SCGB1D2 | -0.069727437 | 0.020620174 |
| KIAA0355 | -0.12548051 | 0.020641527 |
| NBPF14 | 0.184877195 | 0.020727681 |
| MRPL37 | -0.102250648 | 0.020776211 |
| SLITRK4 | -0.17632181 | 0.020785513 |
| TMEM165 | 0.171505095 | 0.020920004 |
| C14orf23 | -0.04322846 | 0.020933073 |
| HNRNPL | 0.130587926 | 0.020953014 |
| ITGAM | 0.300588933 | 0.020971076 |
| SLC7A6 | -0.218025853 | 0.020978645 |
| C12orf10 | -0.082941282 | 0.021011966 |
| KLK9 | 0.137297696 | 0.021033281 |
| AMPD3 | 0.137137337 | 0.021075321 |
| TNFSF13B | 0.155333941 | 0.021088827 |
| CD101 | -0.506645171 | 0.021092683 |
| NLRP3 | 0.20101397 | 0.021097022 |
| LIPE | -0.121636434 | 0.021140884 |
| COBRA1 | -0.074374473 | 0.021157487 |
| ARL8A | 0.161684041 | 0.021161225 |
| CMTM6 | 0.193811383 | 0.021166006 |
| TYSND1 | -0.133475864 | 0.021266211 |
| SLC25A30 | -0.185803226 | 0.021291428 |
| EXD3 | -0.241104016 | 0.021297573 |
| TRUB2 | -0.081345412 | 0.021309475 |
| FAM55D | 0.113934689 | 0.021314788 |
| FLT3LG | -0.318447467 | 0.021324832 |
| SIGMAR1 | -0.107051116 | 0.021382772 |
| SNHG13 | -0.152557037 | 0.021401963 |
| NSUN5 | -0.081146626 | 0.021411021 |
| PDHA2 | -0.074435692 | 0.021452289 |
| USP13 | -0.218046816 | 0.021464636 |
| AUP1 | -0.091962508 | 0.021482644 |
| NPIPL2 | -0.130327841 | 0.021492699 |
| TRMT5 | 0.116939261 | 0.021496578 |
| TBKBP1 | 0.212685496 | 0.021498143 |
| GSX1 | 0.290342023 | 0.021501822 |
| C17orf110 | -0.318134952 | 0.021508213 |
| TM6SF1 | 0.31351362 | 0.021527481 |
| ARPC2 | 0.16421009 | 0.021566912 |
| SPINK2 | -0.491133004 | 0.021606913 |
| RHBDF2 | 0.176603198 | 0.021620459 |
| CNIH | -0.140922254 | 0.02162151 |
| RPS28 | -0.164249467 | 0.021627845 |
| RPL36A | -0.137429032 | 0.02162823 |
| DNAJB7 | -0.077661397 | 0.02163215 |
| GPR68 | -0.223599117 | 0.021749229 |
| IFITM4P | 0.200056564 | 0.02176319 |
| ACRC | 0.129091806 | 0.021763598 |
| OGFOD2 | -0.14560269 | 0.021764678 |
| C9orf24 | -0.302210474 | 0.021787718 |
| ZNF562 | -0.075152758 | 0.021796878 |
| VEGFB | -0.101027021 | 0.021863709 |
| FAM50A | -0.082937561 | 0.021889045 |
| PPP1R3E | -0.183375292 | 0.02190855 |
| MIS18A | -0.091022701 | 0.021934458 |
| TRAF3IP2 | -0.138266521 | 0.021978383 |
| PCYT1A | 0.104272888 | 0.022010079 |
| GZMA | 0.249134986 | 0.022027542 |
| TMEM129 | -0.09802971 | 0.0220556 |
| HIST3H2A | 0.211240844 | 0.022088796 |
| LOC283663 | -0.324351053 | 0.022092702 |
| LRGUK | 0.184181802 | 0.022103741 |
| CYTB | -0.173319781 | 0.022107053 |
| ZC3H12A | 0.117132955 | 0.022113826 |
| NHP2 | -0.100495623 | 0.022151661 |
| TOP1P2 | 0.122921237 | 0.022159706 |
| C19orf63 | -0.126042398 | 0.02221698 |
| CSF3R | 0.15231753 | 0.022241609 |
| TCF20 | 0.163151742 | 0.022242473 |
| OTUD3 | -0.216231831 | 0.022264233 |
| PRPF8 | -0.104659625 | 0.022347369 |
| TNFSF14 | 0.170235445 | 0.022361381 |
| USP11 | -0.104429005 | 0.022364313 |
| TPM2 | -0.089946017 | 0.022387413 |
| ASPRV1 | 0.218204311 | 0.022405242 |
| MYO1F | 0.173637356 | 0.02242942 |
| TSPAN33 | -0.275235044 | 0.022455809 |
| EIF2AK2 | 0.282409063 | 0.022457974 |
| SLC27A6 | 0.199801236 | 0.022513729 |
| KIAA0125 | -0.336893194 | 0.022527095 |
| PPWD1 | -0.081165968 | 0.022530066 |
| C5orf24 | -0.162754892 | 0.022592978 |
| TLR8 | 0.350097057 | 0.022599056 |
| ITCH | 0.092107097 | 0.022631737 |
| GNB4 | 0.171255802 | 0.022634407 |
| MAML1 | 0.114099964 | 0.022643777 |
| TDP2 | 0.219386789 | 0.022664588 |
| FAM127B | -0.111215043 | 0.022687046 |
| RPS27A | -0.094491343 | 0.022704538 |
| PRO1596 | -0.043773745 | 0.02271203 |
| WIPF3 | 0.375862728 | 0.022750555 |
| PAPOLA | 0.094714148 | 0.022796335 |
| NDUFAF4 | -0.192983141 | 0.022820784 |
| SRSF8 | -0.176454441 | 0.022836733 |
| MAP3K5 | 0.142491918 | 0.02283736 |
| H2AFY2 | -0.235735554 | 0.02284528 |
| FGL2 | 0.294306995 | 0.022851076 |
| LOC100130428 | -0.313417921 | 0.022854727 |
| CENPQ | 0.144306079 | 0.022858398 |
| B4GALT7 | -0.131950065 | 0.02288376 |
| EDAR | -0.291138606 | 0.022919976 |
| PDZD11 | -0.079479627 | 0.022933875 |
| SYT17 | -0.363199722 | 0.02293609 |
| ARRDC2 | -0.116397438 | 0.022953586 |
| RRP1B | -0.113065773 | 0.022957803 |
| ACSF3 | -0.104244309 | 0.022968641 |
| FANCF | -0.152919703 | 0.023032299 |
| MRPS21 | -0.140692028 | 0.023053282 |
| ZNF703 | 0.298148548 | 0.023059131 |
| LRRC58 | -0.152439848 | 0.023073209 |
| CD63 | 0.199272208 | 0.023113282 |
| ZSWIM6 | 0.11686537 | 0.023115957 |
| C17orf49 | -0.092032231 | 0.023119979 |
| HPS4 | -0.112792031 | 0.023154914 |
| SNHG5 | 0.293750084 | 0.023160524 |
| SCG3 | 0.179138848 | 0.023202461 |
| KLRB1 | 0.295246303 | 0.02324072 |
| CTDSP1 | 0.09439366 | 0.023273076 |
| SPOPL | 0.158010427 | 0.023368322 |
| CFLAR | 0.1432025 | 0.023398985 |
| IFT140 | -0.183692244 | 0.023414133 |
| MIR17HG | -0.181167331 | 0.023415517 |
| ZNF783 | -0.166126638 | 0.023437443 |
| WDR72 | 0.122397048 | 0.023452374 |
| FXYD5 | -0.106067732 | 0.023464511 |
| FUK | -0.121807828 | 0.023495373 |
| LYPD2 | -0.482511404 | 0.023511312 |
| PYCR2 | -0.098316657 | 0.023516065 |
| MBD3 | -0.133686196 | 0.023520786 |
| CCDC47 | 0.072465159 | 0.023542899 |
| LOC401431 | -0.274523051 | 0.023566415 |
| CD14 | 0.167903501 | 0.023573564 |
| IL21 | 0.155054797 | 0.023577333 |
| KCNN1 | 0.177016763 | 0.023581276 |
| AQP9 | 0.289672698 | 0.023605338 |
| SNW1 | 0.056864459 | 0.023646073 |
| PAPSS2 | -0.229898614 | 0.023668444 |
| CBX5 | -0.116650916 | 0.023676755 |
| TFCP2L1 | -0.411688362 | 0.023742942 |
| URGCP | -0.132424682 | 0.023774368 |
| TIMP2 | 0.179735437 | 0.023791655 |
| EIF2AK4 | -0.145769662 | 0.023809586 |
| CDKL5 | 0.157824801 | 0.023837878 |
| ZIM2 | 0.245025791 | 0.023846318 |
| ACOX3 | -0.155114115 | 0.023946587 |
| KIAA1609 | 0.123361815 | 0.023950285 |
| SLC9A9 | -0.154641148 | 0.024026563 |
| GPRC5D | 0.205865319 | 0.024167941 |
| HMGCL | -0.080799542 | 0.024174087 |
| MGAM | 0.21415242 | 0.024212683 |
| SPNS1 | -0.179788298 | 0.02421544 |
| C11orf61 | -0.250091352 | 0.024223814 |
| ACAN | 0.303588841 | 0.024230972 |
| PHC1 | -0.246071987 | 0.024274098 |
| TMEM105 | 0.215373323 | 0.024277456 |
| FCGR2A | 0.187880095 | 0.024292176 |
| MECR | -0.135759318 | 0.024319498 |
| GUCA1B | -0.233826522 | 0.024364785 |
| CXCR3 | 0.118209826 | 0.024376918 |
| USP17 | 0.375410778 | 0.024407338 |
| PFKM | -0.079810132 | 0.024429124 |
| ACHE | 0.292976401 | 0.024467247 |
| MRPL4 | -0.106155269 | 0.024494406 |
| PCSK7 | -0.139831516 | 0.024535681 |
| IFI16 | 0.236129185 | 0.024540755 |
| RP2 | 0.186513992 | 0.02457789 |
| CENPI | 0.186430142 | 0.024611203 |
| B3GNT8 | 0.12876786 | 0.02462705 |
| CAMSAP1 | -0.208084213 | 0.024723079 |
| LOC100506113 | 0.268985867 | 0.024822895 |
| C1orf38 | 0.141165792 | 0.024858408 |
| SLC36A3 | 0.262919966 | 0.024894946 |
| GPX7 | -0.147808505 | 0.024914022 |
| CD300C | 0.208487461 | 0.024960909 |
| GADD45GIP1 | -0.08890347 | 0.02499329 |
| MACROD1 | 0.144544078 | 0.02500034 |
| LOC100129935 | -0.177890129 | 0.025016868 |
| HOXC13 | -0.176149323 | 0.025023205 |
| FBXL21 | 0.192394242 | 0.025065825 |
| CIRH1A | -0.099323608 | 0.025097905 |
| BIN2 | 0.136740833 | 0.025106772 |
| ZNF843 | 0.176991736 | 0.025107686 |
| ZNF778 | -0.272760112 | 0.025115084 |
| UBR4 | -0.146775425 | 0.025120257 |
| FLJ34690 | 0.152310939 | 0.025159363 |
| SGSH | -0.155452486 | 0.025160237 |
| TEX10 | -0.143250175 | 0.025176156 |
| HP1BP3 | 0.072333885 | 0.025180992 |
| HAUS7 | -0.132856591 | 0.025207628 |
| RILPL2 | 0.135389495 | 0.025235933 |
| FAM65B | 0.211647797 | 0.025246472 |
| AHRR | 0.20922609 | 0.02525876 |
| TBK1 | 0.122959109 | 0.025303512 |
| FAM120A | 0.097738982 | 0.025309199 |
| MAGEF1 | -0.138402968 | 0.025326146 |
| SNHG7 | -0.105931742 | 0.025328763 |
| CYC1 | -0.078906021 | 0.025349646 |
| LOC441455 | 0.144195781 | 0.025350543 |
| GABRD | 0.230839472 | 0.025360502 |
| PARVG | 0.14809835 | 0.025403747 |
| CDK16 | -0.146203734 | 0.025430313 |
| FABP3 | -0.151331343 | 0.025431805 |
| PREX1 | 0.218520408 | 0.025437437 |
| ZNF684 | 0.291709376 | 0.025467479 |
| MYBBP1A | -0.106024053 | 0.025504612 |
| YBEY | -0.204186565 | 0.025526116 |
| TMEM64 | -0.127980459 | 0.02553073 |
| EHMT2 | -0.115552953 | 0.025538232 |
| AZI1 | -0.090787724 | 0.025572032 |
| DPM1 | 0.091329755 | 0.025578606 |
| PELI3 | -0.174359067 | 0.025584596 |
| PRKG2 | -0.091077613 | 0.025623764 |
| NTN5 | -0.17616486 | 0.025639572 |
| DHCR7 | -0.085307109 | 0.025694238 |
| MGC2752 | -0.12521717 | 0.025707177 |
| SYK | 0.114349726 | 0.025735696 |
| SLC2A11 | -0.158774008 | 0.025781252 |
| LOC100509121 | 0.374433303 | 0.025783099 |
| CNDP1 | 0.28595928 | 0.025805297 |
| SOCS4 | -0.24406585 | 0.025815317 |
| TMEM8B | -0.153885521 | 0.025815692 |
| ATP13A2 | -0.112826827 | 0.025840226 |
| HINT2 | -0.089257375 | 0.025855888 |
| S1PR3 | -0.304956346 | 0.025879148 |
| FAM190A | -0.05252651 | 0.025881961 |
| ARHGDIB | 0.176021868 | 0.025887484 |
| LIPG | 0.168614415 | 0.02590774 |
| LOC100129447 | -0.301463085 | 0.025933057 |
| HIST1H3G | 0.128325173 | 0.025970363 |
| FLJ10661 | -0.154589346 | 0.025972542 |
| FCER1G | 0.132878777 | 0.025977168 |
| LOC80054 | -0.355019011 | 0.025980011 |
| SUN1 | -0.17024228 | 0.026012925 |
| OR2L13 | -0.109761059 | 0.026027818 |
| PRSS57 | 0.340680276 | 0.026099417 |
| ZNF48 | -0.108387912 | 0.026103288 |
| C5orf54 | -0.2012955 | 0.026219143 |
| HRSP12 | -0.107879752 | 0.026260429 |
| ZNF767 | -0.139149172 | 0.026270647 |
| NSMCE1 | -0.088734208 | 0.026276576 |
| ZMAT2 | 0.089286367 | 0.02633748 |
| GYPE | -0.285811642 | 0.026346174 |
| CAMK2B | 0.20589483 | 0.026372255 |
| ING5 | -0.111572527 | 0.026403683 |
| MTSS1L | 0.113291292 | 0.026403705 |
| NARS | -0.122346535 | 0.026404415 |
| KRT19P2 | 0.216396732 | 0.026425932 |
| RPL34 | -0.139872013 | 0.026459377 |
| PCLO | 0.109306986 | 0.02647462 |
| C19orf20 | -0.348430929 | 0.026478033 |
| KLRG2 | 0.190594272 | 0.026494783 |
| BRD2 | 0.092488582 | 0.026534774 |
| KLHDC3 | -0.101245132 | 0.026541767 |
| FAM164A | -0.238144727 | 0.026600515 |
| C10orf12 | 0.119600409 | 0.026601587 |
| TGIF2 | -0.191011054 | 0.026606221 |
| DSC1 | -0.560190307 | 0.026612651 |
| OSBPL10 | -0.314232199 | 0.026620405 |
| CAMTA1 | -0.171673854 | 0.026683188 |
| TBC1D10C | 0.093672667 | 0.026695044 |
| UTY | 0.635926052 | 0.026726457 |
| FAM161B | -0.319016311 | 0.026732276 |
| RPS9 | -0.095745424 | 0.026734561 |
| PRPF31 | -0.096505825 | 0.026744377 |
| OR4C15 | 0.342469704 | 0.026788499 |
| GTF2B | 0.111817632 | 0.026794167 |
| ACSL6 | -0.125241836 | 0.026806869 |
| ITPRIP | 0.14171505 | 0.026840937 |
| ANKRD26 | -0.16786969 | 0.026856675 |
| EIF3F | -0.130073708 | 0.026878031 |
| NPTN | 0.22017465 | 0.026884588 |
| C12orf44 | -0.093000595 | 0.026904752 |
| GSPT1 | -0.171208146 | 0.026939469 |
| MOB1A | 0.093269406 | 0.026959175 |
| MAGED1 | -0.108416158 | 0.026970122 |
| NFIL3 | 0.26235909 | 0.027007134 |
| PFDN4 | -0.140351663 | 0.027047916 |
| ACBD6 | -0.090051731 | 0.027127746 |
| C11orf36 | -0.190993081 | 0.027187375 |
| USP21 | -0.181465834 | 0.027206537 |
| C18orf1 | -0.096521378 | 0.027206711 |
| PTGES3 | 0.105942613 | 0.027224187 |
| OGT | 0.108897778 | 0.027227942 |
| JUNB | 0.199852252 | 0.027281979 |
| CENPT | -0.167108692 | 0.027301595 |
| SALL1 | 0.205386756 | 0.027302701 |
| DNAJC15 | -0.163228469 | 0.027321936 |
| NFYA | 0.095025735 | 0.027325357 |
| AKAP11 | -0.170412228 | 0.027388523 |
| LOC100128252 | -0.260281631 | 0.027394576 |
| MAPRE3 | -0.235177792 | 0.027436652 |
| AKIRIN2 | 0.140092769 | 0.027462625 |
| TMEM39B | -0.072101237 | 0.027478492 |
| CD72 | -0.413878372 | 0.027486182 |
| DPY19L2P4 | -0.261165476 | 0.027490588 |
| SEMA4C | -0.149262757 | 0.027498839 |
| LOC728743 | -0.224672208 | 0.027523781 |
| SALL2 | -0.110513294 | 0.027593326 |
| OIP5-AS1 | -0.107057007 | 0.027621328 |
| SGSM3 | -0.109773704 | 0.027635463 |
| CEBPB | 0.201488721 | 0.027669766 |
| TNFRSF1B | 0.134183788 | 0.027689575 |
| CNKSR3 | 0.211823454 | 0.027699843 |
| CST3 | -0.21314766 | 0.027712355 |
| ASF1A | -0.207996159 | 0.027762831 |
| DENND1B | 0.092814155 | 0.027785527 |
| IKBKAP | -0.130463448 | 0.027811305 |
| GPR142 | 0.296876509 | 0.027817891 |
| SLC15A3 | 0.105702202 | 0.02784307 |
| LOC100289079 | -0.231088743 | 0.027843114 |
| ANO9 | -0.246931559 | 0.027861688 |
| DERL1 | 0.077092009 | 0.027867803 |
| LMO2 | 0.135370876 | 0.027872276 |
| SOX14 | 0.073022046 | 0.027879619 |
| ZNF10 | -0.174455957 | 0.027909926 |
| LOC100132014 | -0.066216834 | 0.027926579 |
| TPM3 | 0.100968307 | 0.027937977 |
| PDK2 | -0.089867126 | 0.028028519 |
| TXN | 0.23357366 | 0.028041565 |
| TREML2 | 0.163780304 | 0.028046187 |
| LOC439949 | -0.224214855 | 0.028052338 |
| TM7SF2 | -0.127600014 | 0.028163359 |
| PPAP2B | 0.230634303 | 0.028233636 |
| SERPINA4 | 0.170619729 | 0.028242643 |
| USP49 | -0.267277447 | 0.02827138 |
| ZC3HAV1L | -0.150334454 | 0.028369083 |
| ZNF91 | -0.13487554 | 0.02838679 |
| ISOC1 | -0.140564114 | 0.028414647 |
| ITGBL1 | 0.193389785 | 0.02851308 |
| LOC392288 | 0.180037673 | 0.028515256 |
| TCL6 | -0.100324002 | 0.02852135 |
| RTN4RL1 | -0.194725283 | 0.028528466 |
| ADAM7 | 0.209873786 | 0.02854638 |
| PIN1 | -0.078481395 | 0.028553701 |
| CHMP6 | -0.083165949 | 0.028562529 |
| ANKRD49 | 0.059005747 | 0.028577579 |
| FAM167A | -0.225836154 | 0.028615354 |
| RIC8B | -0.22758398 | 0.028679383 |
| PNPLA8 | -0.136495567 | 0.028693135 |
| NT5E | -0.147722305 | 0.028750728 |
| TMSB4X | 0.118110209 | 0.028767877 |
| RPL27 | -0.089168836 | 0.028803536 |
| DNAH5 | 0.08248462 | 0.028815362 |
| TROVE2 | 0.102582476 | 0.02887846 |
| TMEM53 | -0.101604085 | 0.028883593 |
| FAM159A | -0.306636124 | 0.028923449 |
| PCBD2 | -0.160778314 | 0.028928043 |
| N4BP2L2 | 0.083816726 | 0.028935248 |
| ZEB2 | 0.122980553 | 0.028936888 |
| OSBPL2 | 0.141152814 | 0.028947631 |
| ATF7IP2 | -0.205937617 | 0.028987989 |
| LAPTM5 | 0.151186557 | 0.029025375 |
| TP53INP1 | 0.146675556 | 0.029031708 |
| XPNPEP3 | -0.135132043 | 0.029065166 |
| TCEAL7 | 0.075958216 | 0.029098354 |
| PRO2852 | 0.241742621 | 0.029120329 |
| NCAPD3 | -0.05454871 | 0.029134175 |
| ADCY3 | -0.137227603 | 0.029151358 |
| C3orf26 | -0.168481086 | 0.029161457 |
| GRIP1 | -0.247552469 | 0.029217277 |
| PSG3 | -0.049592797 | 0.029241204 |
| HKR1 | -0.192352947 | 0.029251082 |
| MLH1 | -0.131885067 | 0.029282631 |
| DBIL5P | -0.292151628 | 0.029374507 |
| SPTAN1 | -0.161750083 | 0.029376467 |
| H3F3B | 0.236581948 | 0.029414012 |
| SNRPF | -0.151622537 | 0.029431016 |
| THEM4 | -0.178650111 | 0.029439042 |
| TDRD7 | 0.160758707 | 0.029450572 |
| HSFY2 | -0.044605659 | 0.029460228 |
| AP1S2 | -0.180717257 | 0.029475056 |
| CARD8 | 0.096212117 | 0.029514077 |
| CDC42SE1 | 0.143081963 | 0.029514453 |
| LOC389906 | -0.131648386 | 0.02953139 |
| LOC100507377 | 0.147241436 | 0.029558786 |
| PELI1 | 0.125155828 | 0.029562621 |
| FCGR3B | 0.22896847 | 0.029623176 |
| TAGAP | 0.231571168 | 0.029651849 |
| ARPC3 | 0.172974779 | 0.029663205 |
| CXorf59 | -0.068350523 | 0.029707281 |
| DFNA5 | -0.311037841 | 0.029724553 |
| PCDHA9 | -0.105579795 | 0.029757507 |
| PHLPP2 | -0.194136696 | 0.029834219 |
| TRMT61B | -0.284521929 | 0.029858703 |
| IDH3B | -0.079061526 | 0.029864941 |
| WDR54 | -0.102020975 | 0.029941863 |
| RARA | 0.131062691 | 0.029954988 |
| LOC730755 | 0.186913435 | 0.029970385 |
| C19orf25 | -0.078404682 | 0.029992761 |
| UBE2G2 | -0.081321336 | 0.030033922 |
| SMARCA4 | -0.097531953 | 0.030069113 |
| IL13RA1 | 0.239763976 | 0.030070297 |
| WBSCR22 | -0.136589573 | 0.030076841 |
| SRGAP2P1 | 0.167045911 | 0.030077244 |
| DRD3 | 0.261415435 | 0.03009229 |
| UBXN4 | 0.122547675 | 0.030095662 |
| ZNF746 | 0.148791685 | 0.03018824 |
| TAB2 | 0.109313442 | 0.030201166 |
| NDUFA7 | -0.08326077 | 0.030220227 |
| FBXO38 | 0.135552036 | 0.030254181 |
| SNAP25 | 0.203560181 | 0.030261495 |
| CNIH4 | 0.165680615 | 0.030278133 |
| BIVM | -0.210754871 | 0.030288139 |
| ENO1-AS1 | -0.134897754 | 0.030303081 |
| G2E3 | -0.149638946 | 0.030306816 |
| GDPD2 | 0.194837601 | 0.030308923 |
| MST1R | -0.256521946 | 0.030334628 |
| MINK1 | 0.113689253 | 0.030343381 |
| LILRA3 | 0.185063046 | 0.030371138 |
| IL11RA | -0.164695246 | 0.030372548 |
| AUH | 0.084824588 | 0.030377578 |
| PFKL | -0.099386239 | 0.030379068 |
| DNAJA2 | 0.130946585 | 0.030391111 |
| ARHGAP25 | 0.167801986 | 0.030429754 |
| DOK6 | -0.176944732 | 0.030448267 |
| SFTA1P | -0.142364946 | 0.030544347 |
| UBE2T | -0.134100288 | 0.030548433 |
| NICN1 | -0.113607002 | 0.030548577 |
| PDGFRB | 0.306819063 | 0.030568184 |
| ENPEP | 0.111670486 | 0.030570036 |
| LOC401098 | 0.054395309 | 0.030577757 |
| RPL22 | -0.141379027 | 0.030579455 |
| TRIM8 | 0.099396675 | 0.030607699 |
| WDR87 | -0.066818702 | 0.030635239 |
| IGIP | -0.156768288 | 0.030650894 |
| LOC100505894 | 0.257630875 | 0.030667845 |
| PPFIBP2 | -0.133434999 | 0.030675828 |
| HK3 | 0.190668369 | 0.030681087 |
| SPP1 | -0.30451029 | 0.030689951 |
| HENMT1 | 0.122699852 | 0.030714822 |
| PRICKLE1 | -0.238627834 | 0.030717247 |
| IL6R | 0.175197623 | 0.0307194 |
| GIMAP7 | 0.194802939 | 0.030784515 |
| FBXW10 | -0.200966735 | 0.030787309 |
| USP5 | -0.130636404 | 0.030792849 |
| ZZZ3 | -0.166849504 | 0.030905963 |
| SUMO1 | 0.169079052 | 0.030921437 |
| IPO13 | -0.164854309 | 0.030936324 |
| LSP1 | 0.118184025 | 0.030962597 |
| LOC100506528 | 0.171582209 | 0.030971974 |
| UGT2A3 | 0.050510902 | 0.030978855 |
| HLA-A | 0.144117037 | 0.030983067 |
| RIPK1 | 0.105078195 | 0.031001416 |
| LAPTM4B | -0.201198048 | 0.03106486 |
| WDR43 | -0.133749385 | 0.031084402 |
| CNBD1 | -0.08979338 | 0.031086506 |
| ZNF689 | -0.134136129 | 0.031097523 |
| NDNF | 0.193110417 | 0.031124722 |
| C1QTNF3 | -0.18025053 | 0.031151631 |
| GPBAR1 | 0.16320189 | 0.031157549 |
| MDFI | 0.108734596 | 0.031163003 |
| S100A6 | 0.233147677 | 0.031181596 |
| PI4KB | -0.073410274 | 0.031199476 |
| RTTN | -0.152218835 | 0.031216295 |
| GOLGA2P2Y | -0.253437701 | 0.031236038 |
| NOL12 | 0.071885655 | 0.031251578 |
| RNF130 | 0.138723768 | 0.031259095 |
| KHNYN | -0.070705847 | 0.031259261 |
| PSMC3 | -0.088949084 | 0.03128036 |
| NAB1 | 0.106737349 | 0.031285987 |
| BCL2L1 | -0.190457922 | 0.03133206 |
| ADRA1D | 0.15729599 | 0.031351403 |
| ZNF777 | -0.122161075 | 0.031357705 |
| LOC100652733 | -0.178257764 | 0.031407312 |
| BCL11A | -0.204013634 | 0.031420804 |
| TMEM71 | 0.210467829 | 0.031428585 |
| SNRNP27 | 0.093661532 | 0.03144416 |
| DEFB129 | 0.182983406 | 0.031465729 |
| ANKRD46 | -0.081363259 | 0.031468546 |
| PPP1R3D | 0.192688006 | 0.031500015 |
| NAA25 | -0.235265185 | 0.031503595 |
| PLEKHO2 | 0.101118701 | 0.031543558 |
| POMT2 | -0.125826893 | 0.031562169 |
| RFX6 | -0.056405748 | 0.031599142 |
| TMEM114 | 0.18243013 | 0.031610854 |
| GOLIM4 | 0.180122327 | 0.031629609 |
| DHRS9 | 0.158821072 | 0.031660259 |
| HAUS5 | -0.26847021 | 0.031674462 |
| XRCC4 | 0.135178643 | 0.031674999 |
| MRC1 | -0.448741655 | 0.031723216 |
| ANKRD30BP2 | 0.148768203 | 0.031757962 |
| OR4S1 | -0.156908858 | 0.03178464 |
| HTR2A | 0.163323143 | 0.031796385 |
| MCM2 | -0.142054833 | 0.031826826 |
| TRA2A | 0.11688193 | 0.03187289 |
| APEX1 | -0.101267946 | 0.031882102 |
| CCDC142 | -0.101959471 | 0.031928285 |
| PON3 | 0.257909774 | 0.031995171 |
| SIGLEC5 | 0.203782104 | 0.032026039 |
| CACNB3 | 0.097416521 | 0.032049001 |
| CYB5R4 | 0.173603971 | 0.032081822 |
| KBTBD6 | -0.37848729 | 0.032086284 |
| MADD | -0.110037281 | 0.032107089 |
| BPTF | 0.061715136 | 0.032123217 |
| PARP6 | -0.078798657 | 0.032169264 |
| MCTP2 | 0.229463888 | 0.032202726 |
| C1orf63 | 0.103777022 | 0.032240005 |
| MT1M | -0.189837246 | 0.032328966 |
| DYSF | 0.138277352 | 0.032341044 |
| PTCD2 | -0.154261889 | 0.032345401 |
| PLCXD1 | -0.140205717 | 0.032392578 |
| CSF1R | 0.184685704 | 0.03243203 |
| DPH5 | -0.094706161 | 0.032437448 |
| BTBD2 | -0.103977351 | 0.032457178 |
| COG2 | -0.121346461 | 0.032503103 |
| CATSPER2 | -0.175984322 | 0.032510447 |
| LOC100505869 | 0.412947999 | 0.032516787 |
| LOC100505915 | -0.188164602 | 0.032584761 |
| NUDCD1 | -0.168855911 | 0.032597984 |
| FAM160B2 | -0.122739414 | 0.032611948 |
| LCE1C | 0.119269432 | 0.03263155 |
| CCDC28A | 0.083728703 | 0.032645783 |
| PRRT2 | -0.170612486 | 0.032647234 |
| PHF21B | 0.177562882 | 0.032654828 |
| LYRM4 | -0.117334528 | 0.032655868 |
| PDIK1L | -0.143094935 | 0.032663048 |
| CHMP5 | 0.173009481 | 0.032664606 |
| ST6GAL1 | -0.216012873 | 0.032670015 |
| PLEKHG1 | -0.414720581 | 0.032676335 |
| AQP2 | 0.102749727 | 0.032755362 |
| C11orf95 | -0.213608214 | 0.03278277 |
| BRI3 | 0.182998548 | 0.03279305 |
| SLC2A9 | -0.179469054 | 0.032815104 |
| MTR | -0.138310532 | 0.032817694 |
| TBCK | -0.137190271 | 0.03292465 |
| MB21D1 | 0.171326155 | 0.032959751 |
| ABCA3 | -0.125348168 | 0.032966652 |
| LOC152217 | -0.100688579 | 0.032967931 |
| R3HDM2 | 0.06894744 | 0.032971068 |
| SMPX | 0.209077412 | 0.032978466 |
| NFKBIL1 | -0.190091217 | 0.033008614 |
| CD37 | 0.14070896 | 0.033022233 |
| PFN1P2 | 0.096785098 | 0.033042459 |
| ZNF835 | -0.235081637 | 0.033042801 |
| IDH1 | 0.119150271 | 0.033056907 |
| HLA-DRA | 0.210096172 | 0.03307048 |
| OR5AK2 | -0.064019817 | 0.033072706 |
| CEP135 | -0.149797128 | 0.033092453 |
| CELF5 | -0.188223408 | 0.03309432 |
| KIF26B | 0.133323163 | 0.033127781 |
| C21orf104 | 0.106499076 | 0.033155741 |
| GPC6 | 0.139096713 | 0.033168244 |
| ZFAND5 | 0.085816477 | 0.033185594 |
| NUP85 | -0.082127258 | 0.033215525 |
| SCTR | 0.120267245 | 0.033273424 |
| DNAJC21 | -0.158418073 | 0.033320892 |
| CRYGS | -0.146481934 | 0.03336205 |
| KDM2A | 0.118599576 | 0.033367864 |
| PCCA | -0.117227543 | 0.033392155 |
| ULBP1 | 0.08819232 | 0.033410895 |
| EIF2C3 | 0.094720293 | 0.033416828 |
| C1orf51 | 0.154927513 | 0.033477275 |
| DLX1 | 0.145162632 | 0.033495823 |
| OMP | -0.287402829 | 0.033505401 |
| C2orf81 | -0.257373432 | 0.0335166 |
| FASN | -0.205658138 | 0.033525357 |
| ZFP28 | -0.115880915 | 0.033536669 |
| RNF19B | 0.199211083 | 0.033539245 |
| SHPRH | -0.096314962 | 0.033612065 |
| MGA | -0.134625314 | 0.033668526 |
| ZC3HC1 | -0.089496304 | 0.033721528 |
| POLR2E | -0.090494824 | 0.033750707 |
| MBTD1 | -0.137917231 | 0.033764743 |
| POTEM | 0.17026212 | 0.033779827 |
| MEOX1 | -0.314381305 | 0.03378515 |
| COX2 | -0.152882995 | 0.033788686 |
| SPINT2 | -0.101187924 | 0.033820312 |
| MEF2A | 0.207889012 | 0.033825479 |
| EI24 | -0.093516455 | 0.033901457 |
| CYB5D2 | -0.070249515 | 0.033910819 |
| DOK1 | 0.096339849 | 0.033930514 |
| VEZT | -0.131564989 | 0.033956896 |
| SERF1B | -0.098601324 | 0.03397661 |
| RAP1A | 0.162215927 | 0.03408076 |
| SERTAD2 | -0.152219897 | 0.034086687 |
| FFAR2 | 0.160185838 | 0.034154784 |
| POLR3F | -0.115698694 | 0.034167008 |
| ZNF503-AS1 | 0.18859771 | 0.034240319 |
| TRIM31 | -0.075232087 | 0.034262405 |
| SSH2 | 0.19417325 | 0.03427872 |
| XRCC1 | -0.066487706 | 0.03429619 |
| ECSIT | -0.081301453 | 0.034353548 |
| YWHAZ | 0.105909108 | 0.034443259 |
| EFNA3 | 0.195202969 | 0.034448925 |
| MYL12A | 0.169356028 | 0.034458384 |
| PSTPIP1 | 0.134174182 | 0.034588413 |
| EIF1 | 0.145693673 | 0.03460135 |
| HMGB2 | 0.303741928 | 0.034659338 |
| ZNF691 | -0.121973267 | 0.034682464 |
| KIAA0100 | -0.074152071 | 0.034692718 |
| AKR7A2 | -0.114743775 | 0.034754473 |
| ZNF227 | -0.299344129 | 0.034768001 |
| FAM166B | 0.203775365 | 0.034768948 |
| CTNNB1 | 0.106420903 | 0.034779448 |
| CKLF | 0.150464251 | 0.034786806 |
| CRISPLD2 | 0.231523255 | 0.034850214 |
| KLC4 | -0.184457885 | 0.034863509 |
| MTERFD2 | -0.150583354 | 0.034887065 |
| HBB | 0.392303059 | 0.03491202 |
| GRB10 | 0.416032701 | 0.034959594 |
| PDCD5 | -0.146489347 | 0.034972486 |
| SMCR5 | 0.116603461 | 0.034973031 |
| MX1 | 0.296961014 | 0.035052123 |
| ZNF7 | -0.075664537 | 0.035085195 |
| MSL2 | 0.119538419 | 0.035105126 |
| SCLT1 | 0.127821074 | 0.03513292 |
| GPD1L | -0.149030144 | 0.035133839 |
| HIF3A | 0.113638689 | 0.035135536 |
| IFITM3 | 0.204919642 | 0.035141956 |
| PKIA | -0.221187239 | 0.035142901 |
| DTWD2 | -0.198533897 | 0.035179014 |
| GCA | 0.316267816 | 0.035209476 |
| ZNF513 | -0.099097328 | 0.035229357 |
| EXPH5 | -0.214824736 | 0.035259428 |
| PHLDA1 | -0.114136422 | 0.035263362 |
| TRPT1 | -0.05980658 | 0.035263821 |
| SAV1 | -0.151454669 | 0.035289774 |
| TANK | 0.149344503 | 0.03530521 |
| FRMD5 | 0.17393655 | 0.035306049 |
| PRPF19 | -0.145972029 | 0.035307634 |
| ERAP2 | -0.326941487 | 0.035308888 |
| EPHA8 | 0.100819674 | 0.035311856 |
| SNRK | 0.111121762 | 0.035329405 |
| TUBGCP6 | -0.114344487 | 0.03535126 |
| ABCD2 | -0.389548611 | 0.035373979 |
| GIMAP2 | 0.104078579 | 0.035429434 |
| CDC5L | 0.042365994 | 0.035498567 |
| CDC42EP3 | 0.151079412 | 0.035506783 |
| RXRA | 0.112533415 | 0.035520448 |
| FAM76A | -0.110571702 | 0.035537833 |
| ABCF3 | -0.069188586 | 0.035545306 |
| C4orf26 | -0.07528885 | 0.03556921 |
| RPP30 | -0.106019377 | 0.035579343 |
| TFAP4 | 0.227869632 | 0.03558101 |
| 7-Mar | 0.158849842 | 0.035581655 |
| CHD1 | 0.096694062 | 0.035647447 |
| BCAR1 | 0.091182025 | 0.035671967 |
| POLE3 | -0.094874881 | 0.035740618 |
| PP12719 | 0.251846906 | 0.035740862 |
| UXT | -0.099280656 | 0.03575125 |
| DHX37 | -0.090342362 | 0.035755571 |
| DNM1P46 | 0.171307277 | 0.035760604 |
| MYL12B | 0.133902795 | 0.035798237 |
| HVCN1 | 0.130427295 | 0.035803449 |
| TSG101 | 0.09228538 | 0.035813456 |
| CTNNBL1 | -0.086112063 | 0.035866128 |
| PCBP1 | 0.122677699 | 0.035908833 |
| C11orf65 | -0.215179626 | 0.03594015 |
| SPEF2 | -0.160381393 | 0.035946801 |
| RPL35A | -0.092117631 | 0.035969653 |
| HSPA1A | 0.227797604 | 0.036014662 |
| MASP1 | 0.127261266 | 0.036018002 |
| IDI1 | 0.244973554 | 0.036087918 |
| RPS12 | -0.163938011 | 0.036106025 |
| TMEM191A | -0.116054735 | 0.036132978 |
| ANO6 | -0.186553915 | 0.036171583 |
| DPEP2 | 0.180535334 | 0.036179092 |
| NCAPD2 | -0.100111256 | 0.036196908 |
| E4F1 | -0.128009476 | 0.036210592 |
| FAM111A | 0.133613362 | 0.036249009 |
| RASGRP3 | -0.366792831 | 0.036250927 |
| RING1 | -0.077258962 | 0.036266414 |
| KDELC1 | -0.157897614 | 0.036279419 |
| RABL2A | -0.137853856 | 0.036317636 |
| THRAP3 | 0.082210309 | 0.036346283 |
| C3orf24 | -0.072692092 | 0.03641902 |
| ANKRD13A | 0.130264562 | 0.036441116 |
| NHP2L1 | -0.107995585 | 0.036443083 |
| POU6F1 | -0.187223429 | 0.036463892 |
| LMNA | -0.143851505 | 0.036501135 |
| PNMA3 | -0.202634981 | 0.036512828 |
| ACAD8 | 0.100870366 | 0.036533349 |
| ZNF799 | -0.247271285 | 0.036538942 |
| HMGB1 | 0.101259605 | 0.036555714 |
| DOM3Z | -0.105270392 | 0.036670977 |
| RALGPS2 | -0.252066276 | 0.036672711 |
| MGC4473 | 0.189445233 | 0.036706279 |
| VPREB3 | -0.307569013 | 0.03671026 |
| C12orf76 | -0.120599285 | 0.036711387 |
| SSBP2 | -0.185063173 | 0.036774205 |
| C5orf15 | -0.081146073 | 0.036808262 |
| ZFAND3 | 0.133506132 | 0.036826809 |
| NME4 | -0.122466285 | 0.036833604 |
| LOC100192204 | -0.124145114 | 0.036862302 |
| DTX1 | -0.179268458 | 0.03686802 |
| ZNF576 | -0.11122267 | 0.036869571 |
| ZFP90 | -0.145769179 | 0.0369107 |
| PXN | 0.142777751 | 0.036911162 |
| PITX2 | 0.172862409 | 0.036917107 |
| PLXNC1 | 0.213609021 | 0.036943851 |
| UBE2W | 0.13351279 | 0.036981375 |
| HOXA13 | 0.151794773 | 0.037030581 |
| C20orf4 | -0.084276791 | 0.037040504 |
| ACTR3 | 0.123670548 | 0.037061131 |
| NSL1 | 0.128804834 | 0.03706673 |
| PLD6 | -0.185752769 | 0.037069375 |
| GPBP1L1 | 0.108891388 | 0.037084896 |
| CASC3 | 0.147534727 | 0.037111131 |
| OIT3 | -0.080930859 | 0.037148111 |
| ZNF438 | 0.160746898 | 0.037159003 |
| LOC100505663 | -0.071347334 | 0.037164502 |
| H2AFZ | 0.120354249 | 0.037248255 |
| NPR2 | -0.292235026 | 0.037271733 |
| OGDH | -0.099181174 | 0.037279848 |
| ILK | -0.093055282 | 0.037290066 |
| USP39 | 0.065705572 | 0.037291132 |
| PIEZO2 | 0.105927334 | 0.037329606 |
| CCDC80 | -0.142884556 | 0.037386985 |
| ZNF230 | 0.22750335 | 0.037410746 |
| PKIG | -0.162663065 | 0.037428755 |
| ARTN | -0.189058945 | 0.037441178 |
| KIF12 | 0.093506706 | 0.037469156 |
| NFE4 | 0.368102665 | 0.037471049 |
| LOC100652917 | -0.334981075 | 0.037540381 |
| LASP1 | 0.11933289 | 0.037544731 |
| TRAPPC9 | -0.089872945 | 0.037551197 |
| VASP | 0.212174946 | 0.037557218 |
| MVK | 0.141892161 | 0.037560929 |
| CHI3L2 | -0.235260702 | 0.03756386 |
| DMD | -0.072144244 | 0.037608711 |
| SARNP | 0.0890836 | 0.037656429 |
| TMEM140 | 0.13517468 | 0.037673944 |
| CLPX | 0.066988662 | 0.037727466 |
| NSMAF | 0.155750758 | 0.037804785 |
| RBM8A | 0.085140914 | 0.037823666 |
| MPV17L2 | -0.082999704 | 0.03783045 |
| DIRC1 | 0.286396149 | 0.037885091 |
| GGT1 | -0.112072359 | 0.037899002 |
| KCNJ15 | 0.228679926 | 0.03795798 |
| LRAT | 0.110636058 | 0.038021082 |
| XAB2 | -0.202133692 | 0.038132675 |
| RAPGEF6 | -0.131860241 | 0.038140905 |
| LGR4 | -0.157550142 | 0.038169904 |
| CXCR1 | 0.201957941 | 0.038173377 |
| C12orf57 | -0.126404002 | 0.038273261 |
| ARMC3 | -0.172704927 | 0.038276391 |
| ADRB2 | 0.120465312 | 0.038350637 |
| BCR | -0.078424342 | 0.038376854 |
| ZSCAN18 | -0.171762962 | 0.03840068 |
| PRDM13 | -0.24713638 | 0.038428046 |
| RECQL5 | -0.12532975 | 0.038487588 |
| ATP5G2 | -0.095361204 | 0.03852613 |
| ADIPOR1 | 0.161284658 | 0.038540935 |
| SNTB1 | 0.167042064 | 0.038593981 |
| C1orf35 | -0.133750231 | 0.038613585 |
| LOC653075 | -0.111808821 | 0.038619741 |
| GPR22 | -0.048682866 | 0.038621141 |
| VPS11 | -0.084264848 | 0.038625305 |
| SRSF3 | 0.09050822 | 0.038649577 |
| DUSP1 | 0.25969598 | 0.038724861 |
| AKAP13 | 0.126325569 | 0.038741434 |
| LOC541471 | 0.102740166 | 0.038744151 |
| PDE8B | -0.190940777 | 0.038760137 |
| FOXN3 | 0.098492891 | 0.038809545 |
| PRSS1 | 0.119734464 | 0.038847519 |
| DDX4 | -0.036753363 | 0.038871325 |
| IL6 | -0.167468243 | 0.03887424 |
| SPOCK1 | -0.27132327 | 0.038905484 |
| RPL24 | -0.07012013 | 0.038911795 |
| NUP133 | -0.088017958 | 0.038937472 |
| SMARCA2 | 0.086611699 | 0.038938476 |
| C20orf27 | -0.167674097 | 0.0389863 |
| AADAT | -0.19343684 | 0.039012567 |
| C11orf51 | -0.112245173 | 0.039064248 |
| NEK8 | 0.179629093 | 0.039075717 |
| POM121L1P | 0.26141958 | 0.03913254 |
| IFT74 | -0.187584942 | 0.039140489 |
| FAM89B | 0.066365441 | 0.039173917 |
| STAT1 | 0.190171373 | 0.039183359 |
| LOC441124 | 0.166756105 | 0.03918949 |
| RBFOX1 | 0.120979142 | 0.039232999 |
| WDR59 | -0.090246553 | 0.039318804 |
| RGAG4 | -0.300656673 | 0.039337837 |
| RGS18 | 0.157342539 | 0.03939672 |
| PSME1 | 0.104789101 | 0.039407822 |
| RBMX | -0.098507179 | 0.039441723 |
| BLZF1 | 0.178652684 | 0.039479058 |
| TP53 | -0.201728364 | 0.039503845 |
| GUSBP4 | -0.213760748 | 0.039583609 |
| GMCL1 | 0.129228739 | 0.039598402 |
| ZNF536 | -0.146615298 | 0.039616971 |
| CNGA4 | -0.186536221 | 0.03963075 |
| TATDN1 | -0.105564255 | 0.039644884 |
| RASGRF2 | -0.336438402 | 0.039654625 |
| PSMA6 | 0.079656987 | 0.039658604 |
| AGSK1 | -0.102352641 | 0.039659116 |
| FADS3 | -0.103326561 | 0.039667632 |
| PSTK | -0.177226984 | 0.039682698 |
| SCAPER | -0.167237668 | 0.039721598 |
| ABCF1 | -0.090281896 | 0.039730739 |
| LATS2 | 0.12096194 | 0.039746655 |
| IFIT2 | 0.284255497 | 0.039763095 |
| XRCC5 | 0.074491514 | 0.039768838 |
| OSM | 0.118959151 | 0.039787704 |
| KCNJ2 | 0.156291084 | 0.039817322 |
| NLRP10 | 0.088946891 | 0.039905601 |
| LINC00246A | -0.278867497 | 0.039922441 |
| HIC1 | -0.177861525 | 0.039952963 |
| TTL | -0.095288539 | 0.039967924 |
| PLEKHJ1 | -0.058550218 | 0.039978709 |
| FAM195A | -0.1458197 | 0.040008282 |
| POLG2 | -0.098184907 | 0.040135931 |
| PLXDC1 | -0.357387785 | 0.040165632 |
| SETD1A | -0.260008343 | 0.040191219 |
| C2orf44 | -0.169475834 | 0.04022805 |
| DNAJC14 | 0.099796308 | 0.040255763 |
| GALK2 | 0.059812764 | 0.040300407 |
| LOC100128843 | 0.352790841 | 0.040386728 |
| MRPL44 | 0.094408338 | 0.04042331 |
| LGI1 | 0.181160056 | 0.040473637 |
| CTNND1 | -0.136389995 | 0.040474651 |
| PEBP4 | -0.128161469 | 0.040487884 |
| MLYCD | -0.115874436 | 0.040494812 |
| CDC14B | -0.131862803 | 0.040495382 |
| ACADS | -0.247699785 | 0.040525101 |
| NR1D2 | -0.168559844 | 0.040572491 |
| LOC200726 | -0.178497522 | 0.04059721 |
| SREK1 | 0.06741647 | 0.040687676 |
| BATF2 | 0.272839964 | 0.04069089 |
| CES4A | -0.205684908 | 0.040693288 |
| HIST3H3 | 0.199060752 | 0.040708125 |
| ACD | -0.087423509 | 0.040717111 |
| IL17RD | 0.125133549 | 0.040722511 |
| MYEF2 | -0.320657021 | 0.040765617 |
| 4-Mar | 0.136821343 | 0.040791287 |
| GALNT13 | 0.067729824 | 0.040794349 |
| EPHA1 | -0.262396602 | 0.040795633 |
| UBE2J1 | 0.212765861 | 0.040804302 |
| NDUFS8 | -0.105834768 | 0.040807093 |
| UBAP2 | -0.136469738 | 0.04082305 |
| ZNF264 | -0.138129976 | 0.040840729 |
| CTAGE7P | 0.10113556 | 0.040864372 |
| OR52K3P | 0.30588176 | 0.040951467 |
| ACVR1C | -0.313775119 | 0.040996346 |
| DHX35 | -0.101089547 | 0.041023829 |
| BCL11B | -0.113476509 | 0.041024894 |
| ZBP1 | 0.130804394 | 0.041039906 |
| AGBL1 | 0.154067035 | 0.041067888 |
| HDAC11 | -0.245022639 | 0.041070815 |
| METTL13 | -0.077586196 | 0.041072758 |
| NUDT3 | 0.090188839 | 0.04107333 |
| SLC6A15 | 0.133880775 | 0.041120085 |
| SLC11A1 | 0.155885184 | 0.041145072 |
| MSN | 0.08631396 | 0.041145167 |
| HSP90AA1 | 0.101751084 | 0.041164642 |
| LOC100652828 | -0.1557166 | 0.041191269 |
| CDKN2D | 0.190007552 | 0.041335318 |
| TMEM145 | 0.112915502 | 0.041344628 |
| ANXA7 | 0.084513887 | 0.041355085 |
| C20orf165 | 0.265681247 | 0.041476051 |
| RPS2 | -0.061945945 | 0.041485852 |
| MAP3K8 | 0.124540377 | 0.041504868 |
| CNTROB | -0.076807863 | 0.041525168 |
| BMP15 | -0.045306418 | 0.041566458 |
| ICMT | -0.110095241 | 0.041601457 |
| DIABLO | -0.055763478 | 0.041641328 |
| NKAIN1 | 0.252802752 | 0.041697173 |
| ZNF217 | 0.147940829 | 0.041709696 |
| ZKSCAN5 | -0.13026477 | 0.041712017 |
| ZNF131 | -0.106085881 | 0.041726621 |
| PGK2 | 0.076081645 | 0.041739289 |
| SHMT2 | -0.107274379 | 0.041777763 |
| PDE1C | 0.181560559 | 0.041782128 |
| RASL11A | -0.340212247 | 0.041790253 |
| C21orf59 | -0.13988293 | 0.041814089 |
| ARNT | 0.104910661 | 0.041837192 |
| HSD17B11 | 0.162495435 | 0.04186686 |
| LOC100506930 | -0.124975382 | 0.041869595 |
| MAGOHB | -0.11857843 | 0.041882845 |
| KIAA0195 | -0.115254761 | 0.041901384 |
| ECT2 | 0.138203208 | 0.041910929 |
| ZADH2 | -0.11108557 | 0.041916713 |
| PDHB | -0.092387572 | 0.042058335 |
| ZNF287 | -0.287500116 | 0.042111476 |
| ITFG2 | -0.11567876 | 0.042157837 |
| MAP2K6 | 0.269916388 | 0.042170084 |
| FIBP | -0.080721797 | 0.042175213 |
| PART1 | 0.101832205 | 0.042175894 |
| MAL | -0.260843186 | 0.042178242 |
| NUP155 | -0.099637439 | 0.042197205 |
| NEK2 | 0.207567963 | 0.042208949 |
| SYCE2 | -0.270620129 | 0.042245069 |
| SELT | 0.108712797 | 0.042251161 |
| ANKRD58 | 0.112385737 | 0.042304209 |
| SLC39A3 | -0.111457267 | 0.04232208 |
| TSHZ2 | -0.306091773 | 0.042337732 |
| GAD1 | 0.115501882 | 0.042343532 |
| CRLF2 | -0.188237272 | 0.042363286 |
| LOC643401 | 0.197689559 | 0.042367084 |
| PCDP1 | -0.078253345 | 0.042411711 |
| APOL1 | 0.173274308 | 0.042429597 |
| CST7 | 0.282998519 | 0.042442611 |
| GABBR2 | 0.161366896 | 0.042443639 |
| FMO4 | -0.268698184 | 0.042466503 |
| VHLL | -0.27420505 | 0.042483304 |
| HDHD3 | -0.264024785 | 0.042549391 |
| NPL | 0.157457844 | 0.042552484 |
| ACP2 | -0.172471255 | 0.042578871 |
| TNFAIP3 | 0.200398617 | 0.042654701 |
| AKAP1 | -0.113698872 | 0.042683943 |
| MGRN1 | 0.140743735 | 0.042698354 |
| STX7 | 0.144351926 | 0.042722083 |
| OFD1 | -0.114873729 | 0.042769425 |
| COX6C | -0.162084727 | 0.042782522 |
| DIEXF | -0.146530972 | 0.042786553 |
| KRTAP13-1 | -0.187966933 | 0.042814543 |
| MAT2B | 0.108671833 | 0.042824272 |
| GRIA3 | 0.079351849 | 0.042886 |
| C3orf80 | 0.211573392 | 0.042914728 |
| KPNA1 | 0.215546671 | 0.042918247 |
| HIST2H2AB | 0.18316727 | 0.042923767 |
| PHF19 | -0.075491302 | 0.042928471 |
| VRK2 | 0.118849344 | 0.042948832 |
| REXO1L1 | 0.281958471 | 0.042976205 |
| MAP2K3 | 0.143256213 | 0.042983288 |
| IL22RA2 | -0.055388062 | 0.043044402 |
| MTBP | 0.171898596 | 0.043057075 |
| RASAL3 | 0.126851525 | 0.043057346 |
| RPL29 | -0.109033255 | 0.043143466 |
| ALG9 | -0.115574498 | 0.043162245 |
| BAD | -0.054242553 | 0.043176832 |
| CXADRP2 | 0.208194847 | 0.043183602 |
| DACT1 | -0.292359927 | 0.043186508 |
| DEFA4 | 0.44360751 | 0.043214778 |
| HIST1H2AJ | 0.175496705 | 0.043217567 |
| RHOA | 0.133636315 | 0.043271861 |
| SSH3 | 0.126290505 | 0.043323187 |
| KCTD12 | 0.144615208 | 0.043331376 |
| ELF4 | 0.13059265 | 0.043375619 |
| G6PC | 0.102639314 | 0.043404396 |
| LOC96610 | 0.178446633 | 0.043407646 |
| PUF60 | -0.073366522 | 0.043420749 |
| CD163 | 0.255748563 | 0.043459287 |
| SLC25A35 | -0.173486527 | 0.043475854 |
| HMX1 | -0.252890238 | 0.043479814 |
| TERF2IP | 0.061238794 | 0.043482729 |
| PCDH9 | -0.234306458 | 0.043485007 |
| ANKRD11 | 0.099061632 | 0.04351378 |
| GLTPD2 | 0.227081557 | 0.043547691 |
| MRRF | -0.147507098 | 0.043568655 |
| STAT3 | 0.18361198 | 0.043645921 |
| RAB21 | 0.145024391 | 0.043665117 |
| KISS1 | -0.286384657 | 0.043672384 |
| PAFAH1B3 | -0.081868535 | 0.043676703 |
| ADCK5 | -0.117282056 | 0.043682872 |
| PNMA6A | -0.24406122 | 0.043756104 |
| UBC | 0.194670153 | 0.043784337 |
| MRPL40 | -0.111788787 | 0.043805748 |
| CTAGE4 | 0.077101004 | 0.043809179 |
| SVIP | -0.144328725 | 0.0438256 |
| OXNAD1 | -0.171451088 | 0.043855953 |
| LOC284889 | -0.105334077 | 0.043859775 |
| PTGES | 0.216425012 | 0.043863454 |
| FRAT2 | 0.15429582 | 0.043875385 |
| PRF1 | 0.156383314 | 0.043875492 |
| GPRASP1 | -0.275730405 | 0.043880012 |
| DENND4C | -0.181383695 | 0.043885845 |
| LOC100506687 | 0.166301938 | 0.043890238 |
| LOC100289255 | 0.180063596 | 0.043946942 |
| C4orf48 | -0.104461117 | 0.043970194 |
| AFG3L1P | -0.10742875 | 0.044028661 |
| PLD4 | -0.296966376 | 0.044039542 |
| DDX5 | 0.115573502 | 0.044089084 |
| ROCK1 | 0.131245495 | 0.044107775 |
| PSMF1 | -0.053429948 | 0.044161981 |
| OR51B4 | 0.087642797 | 0.044185849 |
| RNF208 | -0.23824237 | 0.044188483 |
| DDX58 | 0.222852431 | 0.044233321 |
| PHKG2 | -0.110187321 | 0.044268537 |
| PF4 | 0.166939642 | 0.044309219 |
| ARHGAP21 | -0.176836851 | 0.044318622 |
| SRPK1 | 0.277419812 | 0.044330623 |
| PSG4 | -0.182230084 | 0.044336474 |
| TSPAN4 | -0.072092767 | 0.044369305 |
| RASGRP4 | 0.150046487 | 0.044373188 |
| APBA2 | -0.225666354 | 0.044446677 |
| SEPSECS | 0.183452599 | 0.044482301 |
| ZNF525 | -0.165212497 | 0.044501922 |
| ADH1C | 0.205528328 | 0.044502108 |
| HLA-DRB4 | 0.185086319 | 0.044540945 |
| RANGRF | -0.115341472 | 0.044571023 |
| HDHD1 | -0.114933163 | 0.044609887 |
| PCSK4 | -0.137303696 | 0.044633581 |
| C11orf1 | -0.070343741 | 0.044707609 |
| PITPNM1 | -0.119962828 | 0.044710259 |
| PSMD12 | 0.059454745 | 0.044758767 |
| IFNGR2 | 0.147778055 | 0.044766022 |
| GHITM | 0.063026345 | 0.044838244 |
| FAM19A1 | -0.416363354 | 0.044906632 |
| KPNA5 | -0.23686487 | 0.044909586 |
| THG1L | -0.083837257 | 0.044915303 |
| LOC100507431 | -0.063646993 | 0.044954251 |
| TMEM50A | 0.107353198 | 0.044964113 |
| SNX22 | -0.176497144 | 0.045025526 |
| KREMEN2 | -0.197894911 | 0.045082244 |
| IGFBPL1 | 0.138179891 | 0.045116752 |
| DR1 | 0.082818905 | 0.045161763 |
| TCTN3 | -0.130645903 | 0.045171764 |
| FAM8A1 | 0.196253733 | 0.045248727 |
| ABLIM2 | -0.270126869 | 0.045258094 |
| H3F3C | 0.242560151 | 0.045275322 |
| INPP4A | 0.0981227 | 0.045307931 |
| SARM1 | -0.177995699 | 0.045395162 |
| PROK2 | 0.421524203 | 0.045407212 |
| DDX19B | -0.067948089 | 0.045444433 |
| LOC100129973 | -0.220499336 | 0.045457376 |
| DDX51 | -0.072482678 | 0.04547123 |
| DAPP1 | 0.130821167 | 0.045474659 |
| ARL6IP1 | 0.103105833 | 0.045480695 |
| LOC441461 | -0.257389934 | 0.045482118 |
| SLU7 | 0.068101308 | 0.045485524 |
| HTR3C | -0.104862809 | 0.045486493 |
| LOC100144602 | 0.159384042 | 0.0454879 |
| FLJ13224 | -0.194221165 | 0.04549333 |
| IDH3G | -0.079508775 | 0.045506613 |
| LOC100127891 | 0.129157163 | 0.045516217 |
| ANXA8L2 | 0.181295328 | 0.045516909 |
| UROD | -0.078015142 | 0.045517765 |
| POLD4 | -0.057223698 | 0.045519534 |
| H1FOO | -0.106553496 | 0.045578929 |
| NEURL4 | -0.066482385 | 0.045615672 |
| TSTA3 | -0.076560193 | 0.045692062 |
| ATP6V1G1 | 0.118366924 | 0.045712589 |
| LOC100652972 | -0.259108717 | 0.045712784 |
| PCDHB7 | 0.258753582 | 0.045718964 |
| RNF152 | 0.097601731 | 0.045751517 |
| C1orf172 | -0.293609667 | 0.045763408 |
| SLC8A1 | 0.226298495 | 0.04576395 |
| MAB21L2 | 0.422476865 | 0.045767682 |
| PRDM2 | 0.084568671 | 0.04579597 |
| C12orf66 | -0.164809575 | 0.045805104 |
| GRHL1 | 0.260636383 | 0.045810006 |
| NAT6 | -0.116086994 | 0.045878593 |
| AGPAT5 | -0.136191535 | 0.045940125 |
| LOC255177 | -0.049044478 | 0.046020727 |
| TMEM132C | -0.14185244 | 0.046087596 |
| SLC6A12 | -0.418673797 | 0.046108761 |
| EMBP1 | 0.165795419 | 0.046111889 |
| ZNF304 | -0.158761656 | 0.046139467 |
| GHRL | 0.126173962 | 0.046204289 |
| KIAA0319 | 0.286765682 | 0.04621807 |
| KRT76 | 0.231826375 | 0.046222613 |
| TREM1 | 0.173858175 | 0.046234317 |
| SLC35F1 | 0.18228499 | 0.046313736 |
| PCDH1 | 0.150030761 | 0.046353181 |
| SH2D3C | 0.133691872 | 0.046369958 |
| THRSP | -0.098988995 | 0.046374908 |
| SWAP70 | -0.159192474 | 0.046468817 |
| POM121L10P | 0.076792642 | 0.0465233 |
| FLAD1 | -0.062923893 | 0.046532243 |
| FAM92B | 0.120402578 | 0.046620549 |
| MRS2 | -0.124660243 | 0.046692546 |
| EXOSC5 | -0.111056802 | 0.046727354 |
| HEATR6 | -0.114096493 | 0.046791787 |
| EXOC3L2 | 0.109165864 | 0.04682406 |
| CUTA | -0.113829486 | 0.046899601 |
| ABCC11 | 0.197367246 | 0.046908218 |
| SBK2 | 0.203540062 | 0.046915381 |
| LOC100128185 | 0.037951734 | 0.046934532 |
| EDEM2 | 0.123345598 | 0.046940323 |
| C6orf124 | 0.395979053 | 0.046962851 |
| AKTIP | 0.150813047 | 0.046997222 |
| CDK2AP1 | -0.14743236 | 0.047037372 |
| C21orf56 | -0.238612941 | 0.047105691 |
| IL12RB1 | 0.207476874 | 0.047124506 |
| PSMB4 | 0.065102909 | 0.047147258 |
| EDEM1 | 0.115342209 | 0.047165859 |
| KRT35 | 0.13519516 | 0.047295137 |
| P2RX5 | -0.209763453 | 0.047344782 |
| C2orf73 | 0.083789293 | 0.047413067 |
| NARG2 | -0.120631365 | 0.047464241 |
| TPCN1 | -0.155988561 | 0.047491438 |
| ZNF25 | -0.12091972 | 0.047494852 |
| NIT2 | -0.153504017 | 0.047602973 |
| ADRBK1 | 0.082357509 | 0.047647507 |
| ALG8 | -0.120123728 | 0.047657797 |
| SPCS3 | 0.115511649 | 0.047691593 |
| GGPS1 | 0.07152842 | 0.047719438 |
| RNF187 | -0.087098427 | 0.047778363 |
| HMP19 | 0.214673262 | 0.047823946 |
| TMEM25 | -0.175828943 | 0.047871941 |
| STOML2 | -0.070540809 | 0.047884317 |
| GPR112 | 0.094493407 | 0.047898112 |
| ZIK1 | -0.203621463 | 0.047917388 |
| DAOA-AS1 | 0.037495041 | 0.047922422 |
| TEX264 | -0.108106671 | 0.047932907 |
| NFX1 | -0.078317926 | 0.048020295 |
| RAPGEF1 | -0.151523855 | 0.048026386 |
| SLC25A31 | 0.038803455 | 0.048061554 |
| ASPH | 0.308578743 | 0.048079764 |
| PLEKHM3 | 0.084269504 | 0.048080559 |
| MRPL21 | -0.140144362 | 0.048094447 |
| TDRG1 | 0.052298348 | 0.048111959 |
| ANK3 | -0.209396404 | 0.048130961 |
| PPP1CB | 0.144769987 | 0.048135405 |
| LRRTM2 | -0.141296119 | 0.048140902 |
| ANAPC7 | -0.155256792 | 0.048162554 |
| C10orf25 | -0.225322411 | 0.048195819 |
| C12orf50 | 0.100599751 | 0.048210643 |
| TRAPPC10 | 0.078139274 | 0.048218637 |
| MTO1 | -0.090303034 | 0.048248075 |
| PHACTR4 | -0.0923566 | 0.048252591 |
| EPB41L4A-AS1 | -0.148785239 | 0.048280778 |
| HADH | -0.086244547 | 0.048286161 |
| HMBS | -0.114210354 | 0.048333952 |
| EIF4EBP1 | -0.095118476 | 0.048340833 |
| ATP6V0E1 | 0.12233643 | 0.048343878 |
| LOC100505870 | 0.095677548 | 0.048361761 |
| RSAD1 | -0.111686805 | 0.048390439 |
| RPL15 | -0.09434036 | 0.048413146 |
| POLD1 | -0.064728742 | 0.048426078 |
| POLN | 0.081435884 | 0.048501409 |
| C6orf145 | 0.124544224 | 0.048504621 |
| C22orf43 | 0.100441008 | 0.048528475 |
| FXYD4 | 0.239861337 | 0.048587665 |
| MYCBPAP | -0.321322291 | 0.048614878 |
| FAS | 0.178416114 | 0.048633901 |
| TRIP12 | 0.092129793 | 0.048644827 |
| TMEM223 | -0.113947762 | 0.048647107 |
| FAM27E3 | 0.284742529 | 0.048655739 |
| MCL1 | 0.151944262 | 0.048681451 |
| KCTD4 | 0.040465682 | 0.048693292 |
| NELL2 | -0.293381349 | 0.048704713 |
| C17orf70 | -0.104213304 | 0.048720517 |
| IGLL5 | 0.177927924 | 0.048726007 |
| MYCBP2 | -0.104726275 | 0.048744671 |
| RAB30 | -0.2565025 | 0.048755754 |
| BTBD9 | -0.137965489 | 0.048818295 |
| CALCA | 0.078917489 | 0.048826884 |
| LOC100130950 | -0.260273806 | 0.048845982 |
| ATP8A2 | -0.246316878 | 0.048884066 |
| CRYAB | -0.218967289 | 0.048899949 |
| BOLA1 | -0.111267572 | 0.048952055 |
| SPPL2A | 0.084387778 | 0.048959564 |
| C17orf98 | -0.103918438 | 0.048974661 |
| CERS6 | -0.155925687 | 0.048988879 |
| KBTBD8 | -0.233918912 | 0.049009391 |
| MLLT3 | -0.217747229 | 0.049056507 |
| STX1B | 0.189293395 | 0.049066344 |
| ALG1L | -0.099334787 | 0.049098813 |
| PGA3 | -0.387443586 | 0.049101061 |
| POTEKP | 0.167616788 | 0.049110458 |
| GALNT9 | 0.205565364 | 0.049125475 |
| SERPINB6 | -0.134723125 | 0.049144479 |
| CNTN6 | -0.176895552 | 0.049146364 |
| LOC100131662 | -0.261758164 | 0.049197348 |
| KIAA1274 | -0.26399126 | 0.049243269 |
| IRF4 | -0.209985588 | 0.049346699 |
| PKD1P1 | -0.088102 | 0.049347749 |
| LOC100131176 | -0.470021241 | 0.049412061 |
| IL17B | 0.179172665 | 0.049449545 |
| TREML3 | -0.341159733 | 0.049470573 |
| CHMP1B | 0.132720936 | 0.049531601 |
| RFTN2 | 0.065790121 | 0.049554532 |
| TMX1 | 0.108917209 | 0.049627936 |
| CXCR2P1 | 0.24805605 | 0.049670582 |
| PLAGL1 | 0.156873493 | 0.049693062 |
| PLXNA1 | -0.104192195 | 0.04975537 |
| THSD4 | 0.102115021 | 0.049802637 |
| CADPS2 | 0.176553282 | 0.049823989 |
| HIRIP3 | -0.131045206 | 0.049862988 |
| AGL | -0.10837452 | 0.049866072 |
| GRB2 | 0.103113239 | 0.049866644 |
| FAM91A1 | 0.100262354 | 0.049876277 |
| RPL13AP3 | -0.069307036 | 0.049920302 |
| POU5F2 | -0.175436547 | 0.049950563 |
| CMTM2 | 0.265762721 | 0.049966637 |
|  |  |  |
|  |  |  |
|  |  |  |
|  |  |  |
|  |  |  |
|  |  |  |
|  |  |  |
|  |  |  |
|  |  |  |
|  |  |  |
|  |  |  |
|  |  |  |
|  |  |  |
|  |  |  |
|  |  |  |
|  |  |  |
|  |  |  |
|  |  |  |
|  |  |  |
|  |  |  |
|  |  |  |
|  |  |  |
|  |  |  |
|  |  |  |
|  |  |  |
|  |  |  |
|  |  |  |
|  |  |  |
|  |  |  |
|  |  |  |
|  |  |  |
|  |  |  |
