# Supplementary material for: NCF2, MYO1F, S1PR4, and FCN1 as potential noninvasive diagnostic biomarkers in patients with obstructive coronary artery: A weighted gene co‐expression network analysis
Source: J Cell Biochem. 2019 Jun 27;120(10):18219–35. doi: 10.1002/jcb.29128 (PMC6771964; doi:10.1002/jcb.29128)
Supplement: Supplementary file 3 — Supporting information [file JCB-120--s001.doc]

| **Supplemental Table 2** Genes in different modules | | | | |
| --- | --- | --- | --- | --- |
| substanceBXH | log2(FC) | pValue | moduleColor | co-expression node |
| FGL2 | 0.294306995 | 0.022851076 | blue | 5 |
| FCGR3B | 0.22896847 | 0.029623176 | blue | 7 |
| MYO1F | 0.173637356 | 0.02242942 | blue | 8 |
| MNDA | 0.289550867 | 0.015782963 | blue | 8 |
| CSF1R | 0.184685704 | 0.03243203 | blue | 8 |
| ALOX5 | 0.252370309 | 0.007757941 | blue | 9 |
| S100A9 | 0.301430542 | 0.005102694 | blue | 10 |
| NCF2 | 0.380059845 | 0.001307676 | blue | 11 |
| S1PR4 | 0.230848352 | 0.004159813 | blue | 11 |
| CLEC4E | 0.263951951 | 0.010898627 | blue | 15 |
| AMICA1 | 0.279457588 | 0.005964466 | blue | 17 |
| FCN1 | 0.216502669 | 0.019164154 | blue | 20 |
| TLR2 | 0.260152248 | 0.005174424 | blue | 22 |
| B2M | 0.215354721 | 0.000188161 | blue | 0 |
| PRDM1 | 0.268573373 | 0.000268528 | blue | 0 |
| ISG20 | 0.252022966 | 0.000302391 | blue | 0 |
| VPS52 | -0.198957711 | 0.000498393 | blue | 0 |
| XYLT1 | -0.29580941 | 0.000589409 | blue | 0 |
| GLIPR2 | 0.201016977 | 0.000595202 | blue | 0 |
| AIF1 | 0.21673292 | 0.000703221 | blue | 0 |
| GBP2 | 0.222764426 | 0.000872115 | blue | 0 |
| PARP9 | 0.259747896 | 0.000990681 | blue | 0 |
| PEMT | -0.198586186 | 0.000997281 | blue | 0 |
| TCEA2 | -0.194612102 | 0.001095556 | blue | 0 |
| VNN2 | 0.29022891 | 0.001107171 | blue | 0 |
| TNFAIP2 | 0.233277504 | 0.00123362 | blue | 0 |
| TNRC6B | 0.137903374 | 0.001383234 | blue | 0 |
| FYB | 0.177090733 | 0.001460076 | blue | 0 |
| HSD17B10 | -0.150470011 | 0.001622988 | blue | 0 |
| P2RY13 | 0.379450564 | 0.001690245 | blue | 0 |
| C19orf59 | 0.32105415 | 0.001691815 | blue | 0 |
| ADAM8 | 0.249951847 | 0.001849304 | blue | 0 |
| LAT2 | 0.162469174 | 0.001865833 | blue | 0 |
| RBM4B | -0.142353759 | 0.001927155 | blue | 0 |
| CD53 | 0.253291929 | 0.001992363 | blue | 0 |
| DOCK8 | 0.214673056 | 0.002013621 | blue | 0 |
| CD300LF | 0.299317815 | 0.002032841 | blue | 0 |
| LOC648771 | -0.150234609 | 0.00210004 | blue | 0 |
| C1orf52 | 0.158307914 | 0.002243152 | blue | 0 |
| ATG16L2 | 0.218391202 | 0.002304227 | blue | 0 |
| TMEM238 | -0.175317806 | 0.002409164 | blue | 0 |
| CARD17 | 0.200942427 | 0.002507447 | blue | 0 |
| SELL | 0.471717905 | 0.00259897 | blue | 0 |
| LYSMD4 | -0.25350402 | 0.002617653 | blue | 0 |
| STK4 | 0.152969949 | 0.002647294 | blue | 0 |
| APBB1IP | 0.220307253 | 0.002693564 | blue | 0 |
| IL2RB | 0.245796896 | 0.003248781 | blue | 0 |
| GMIP | 0.143455453 | 0.003417888 | blue | 0 |
| NDUFB11 | -0.144765088 | 0.003468404 | blue | 0 |
| FPR1 | 0.274616946 | 0.003642391 | blue | 0 |
| WIPF1 | 0.159336203 | 0.003888918 | blue | 0 |
| CENPH | -0.287587016 | 0.003916598 | blue | 0 |
| CD300A | 0.142089477 | 0.003920884 | blue | 0 |
| CCL4 | 0.245530602 | 0.00408827 | blue | 0 |
| SLA | 0.225505558 | 0.004115859 | blue | 0 |
| GIMAP5 | 0.283429777 | 0.004218612 | blue | 0 |
| SP100 | 0.140029186 | 0.004309693 | blue | 0 |
| RCSD1 | 0.172545526 | 0.00434715 | blue | 0 |
| CTSS | 0.167402367 | 0.004446645 | blue | 0 |
| SRGN | 0.315689193 | 0.004521609 | blue | 0 |
| TNFSF10 | 0.192911927 | 0.004685008 | blue | 0 |
| CD93 | 0.285285825 | 0.004719997 | blue | 0 |
| NKRF | -0.163630032 | 0.004732573 | blue | 0 |
| ERGIC3 | -0.146390694 | 0.004754221 | blue | 0 |
| SNRPA | -0.107558791 | 0.005003678 | blue | 0 |
| CDK4 | -0.18396133 | 0.005107547 | blue | 0 |
| PPBP | 0.374815042 | 0.005300449 | blue | 0 |
| DAPK2 | 0.215165308 | 0.005343786 | blue | 0 |
| FLJ32255 | 0.191137409 | 0.005587437 | blue | 0 |
| ATP5D | -0.143081019 | 0.005683427 | blue | 0 |
| LINC00461 | 0.243095409 | 0.00581083 | blue | 0 |
| HIGD2A | -0.119484211 | 0.005920565 | blue | 0 |
| PLXDC2 | 0.286407229 | 0.005955253 | blue | 0 |
| CYTIP | 0.158535744 | 0.005983815 | blue | 0 |
| YIF1A | -0.118336868 | 0.005997857 | blue | 0 |
| F13A1 | 0.2857377 | 0.006002488 | blue | 0 |
| TRIM22 | 0.21418279 | 0.006029641 | blue | 0 |
| GAPT | 0.4335984 | 0.0060825 | blue | 0 |
| NTNG2 | 0.181513323 | 0.006240837 | blue | 0 |
| LOC643802 | 0.293169816 | 0.006303498 | blue | 0 |
| CFP | 0.242844928 | 0.006418266 | blue | 0 |
| RPL12 | -0.107162404 | 0.00643201 | blue | 0 |
| CYTH4 | 0.226361644 | 0.006623404 | blue | 0 |
| DRG2 | -0.123811524 | 0.006727384 | blue | 0 |
| GIMAP6 | 0.18193205 | 0.00693399 | blue | 0 |
| DOK3 | 0.223238872 | 0.007090508 | blue | 0 |
| PCDHB14 | 0.218207115 | 0.007309885 | blue | 0 |
| DCXR | -0.151570885 | 0.007352665 | blue | 0 |
| POLG | -0.108111773 | 0.00737824 | blue | 0 |
| HSPA6 | 0.185538393 | 0.007390996 | blue | 0 |
| CDH1 | -0.265983841 | 0.007458853 | blue | 0 |
| WBP11 | -0.12868662 | 0.00760189 | blue | 0 |
| KLF2 | 0.167028168 | 0.00786712 | blue | 0 |
| CD27 | 0.17555562 | 0.007901216 | blue | 0 |
| S100A12 | 0.259238872 | 0.008107638 | blue | 0 |
| LCP2 | 0.129211926 | 0.008271977 | blue | 0 |
| VEZF1 | 0.080752676 | 0.008291878 | blue | 0 |
| STX11 | 0.251869089 | 0.008673315 | blue | 0 |
| LOC728855 | 0.122623864 | 0.009591993 | blue | 0 |
| CKAP5 | -0.101399938 | 0.009731901 | blue | 0 |
| SORBS3 | -0.196705216 | 0.009913599 | blue | 0 |
| MPEG1 | 0.297771063 | 0.010269602 | blue | 0 |
| MFSD7 | 0.164826769 | 0.010403585 | blue | 0 |
| KIAA0247 | 0.13701163 | 0.010492175 | blue | 0 |
| STK10 | 0.106967444 | 0.010646205 | blue | 0 |
| C8orf60 | 0.156689511 | 0.01100077 | blue | 0 |
| PLCG2 | 0.126648786 | 0.011137989 | blue | 0 |
| DPM3 | -0.140258289 | 0.011173128 | blue | 0 |
| NUAK2 | 0.190995418 | 0.01150115 | blue | 0 |
| TINF2 | 0.078491194 | 0.011750898 | blue | 0 |
| CEACAM4 | 0.21060757 | 0.011855806 | blue | 0 |
| ZCRB1 | -0.154321237 | 0.011879322 | blue | 0 |
| CYTH1 | 0.132676009 | 0.011925986 | blue | 0 |
| IL17RA | 0.199594557 | 0.012023693 | blue | 0 |
| BST1 | 0.180473936 | 0.012117483 | blue | 0 |
| CLEC4D | 0.357478967 | 0.012239338 | blue | 0 |
| FCAR | 0.287322347 | 0.01235064 | blue | 0 |
| IRAK3 | 0.295109544 | 0.012371691 | blue | 0 |
| EVI2B | 0.256799087 | 0.01258289 | blue | 0 |
| CLEC16A | -0.157176541 | 0.012700108 | blue | 0 |
| UBE2I | -0.088920128 | 0.012814602 | blue | 0 |
| DENND1C | 0.14676696 | 0.012850642 | blue | 0 |
| GNLY | 0.293619745 | 0.013098914 | blue | 0 |
| PRKCD | 0.133374786 | 0.01319529 | blue | 0 |
| GZMK | 0.258202697 | 0.013515818 | blue | 0 |
| PLEK | 0.239778374 | 0.013530925 | blue | 0 |
| ZNF580 | -0.127464653 | 0.013532927 | blue | 0 |
| CDK2AP2 | -0.115911609 | 0.013591872 | blue | 0 |
| TLR6 | 0.238122791 | 0.013703308 | blue | 0 |
| B3GALT4 | 0.089997503 | 0.014026428 | blue | 0 |
| HPRT1 | -0.111287992 | 0.014189129 | blue | 0 |
| TFPT | -0.076126008 | 0.014641021 | blue | 0 |
| PECAM1 | 0.146228485 | 0.014705002 | blue | 0 |
| TMEM154 | 0.268438901 | 0.014971256 | blue | 0 |
| LRRC25 | 0.271065006 | 0.01497592 | blue | 0 |
| GIMAP8 | 0.167358757 | 0.015738891 | blue | 0 |
| PTBP1 | -0.198090132 | 0.015868023 | blue | 0 |
| CCL3 | 0.144776923 | 0.015890119 | blue | 0 |
| RPL39 | -0.124063626 | 0.016122009 | blue | 0 |
| GABPB2 | -0.153922278 | 0.01681114 | blue | 0 |
| FAM49A | 0.167207535 | 0.017012121 | blue | 0 |
| NCF4 | 0.180785648 | 0.01702263 | blue | 0 |
| IL1R2 | 0.300592503 | 0.017227279 | blue | 0 |
| LOC650226 | 0.254237524 | 0.017637943 | blue | 0 |
| DBT | -0.178494817 | 0.017778479 | blue | 0 |
| LTB | 0.191075654 | 0.017813517 | blue | 0 |
| DDX46 | -0.071342436 | 0.018096945 | blue | 0 |
| LOC727820 | 0.13285631 | 0.018162799 | blue | 0 |
| ARRB2 | 0.132965258 | 0.018166641 | blue | 0 |
| TLR4 | 0.18197456 | 0.018186966 | blue | 0 |
| MYBPC3 | 0.186895603 | 0.018359279 | blue | 0 |
| NDUFC1 | -0.151254393 | 0.018768266 | blue | 0 |
| RGS2 | 0.289307264 | 0.018955196 | blue | 0 |
| SLC7A7 | 0.182945553 | 0.01896501 | blue | 0 |
| POTEE | 0.159299708 | 0.019171385 | blue | 0 |
| MS4A7 | 0.242059946 | 0.019326804 | blue | 0 |
| C19orf38 | 0.147548433 | 0.01937843 | blue | 0 |
| EIF2AK3 | -0.256484174 | 0.01937972 | blue | 0 |
| CRELD1 | -0.128679078 | 0.019494805 | blue | 0 |
| PRAM1 | 0.141561058 | 0.020497893 | blue | 0 |
| MRPL37 | -0.102250648 | 0.020776211 | blue | 0 |
| ITGAM | 0.300588933 | 0.020971076 | blue | 0 |
| TNFSF13B | 0.155333941 | 0.021088827 | blue | 0 |
| NLRP3 | 0.20101397 | 0.021097022 | blue | 0 |
| SIGMAR1 | -0.107051116 | 0.021382772 | blue | 0 |
| CNIH | -0.140922254 | 0.02162151 | blue | 0 |
| GZMA | 0.249134986 | 0.022027542 | blue | 0 |
| CYTB | -0.173319781 | 0.022107053 | blue | 0 |
| ZC3H12A | 0.117132955 | 0.022113826 | blue | 0 |
| TNFSF14 | 0.170235445 | 0.022361381 | blue | 0 |
| ASPRV1 | 0.218204311 | 0.022405242 | blue | 0 |
| TLR8 | 0.350097057 | 0.022599056 | blue | 0 |
| FAM127B | -0.111215043 | 0.022687046 | blue | 0 |
| PDZD11 | -0.079479627 | 0.022933875 | blue | 0 |
| ACSF3 | -0.104244309 | 0.022968641 | blue | 0 |
| C17orf49 | -0.092032231 | 0.023119979 | blue | 0 |
| EIF2AK4 | -0.145769662 | 0.023809586 | blue | 0 |
| MGAM | 0.21415242 | 0.024212683 | blue | 0 |
| C11orf61 | -0.250091352 | 0.024223814 | blue | 0 |
| FCGR2A | 0.187880095 | 0.024292176 | blue | 0 |
| CXCR3 | 0.118209826 | 0.024376918 | blue | 0 |
| C1orf38 | 0.141165792 | 0.024858408 | blue | 0 |
| BIN2 | 0.136740833 | 0.025106772 | blue | 0 |
| FAM65B | 0.211647797 | 0.025246472 | blue | 0 |
| CYC1 | -0.078906021 | 0.025349646 | blue | 0 |
| PARVG | 0.14809835 | 0.025403747 | blue | 0 |
| CDK16 | -0.146203734 | 0.025430313 | blue | 0 |
| SYK | 0.114349726 | 0.025735696 | blue | 0 |
| FCER1G | 0.132878777 | 0.025977168 | blue | 0 |
| NSMCE1 | -0.088734208 | 0.026276576 | blue | 0 |
| TBC1D10C | 0.093672667 | 0.026695044 | blue | 0 |
| MAGED1 | -0.108416158 | 0.026970122 | blue | 0 |
| USP21 | -0.181465834 | 0.027206537 | blue | 0 |
| LOC728743 | -0.224672208 | 0.027523781 | blue | 0 |
| OIP5-AS1 | -0.107057007 | 0.027621328 | blue | 0 |
| TNFRSF1B | 0.134183788 | 0.027689575 | blue | 0 |
| CST3 | -0.21314766 | 0.027712355 | blue | 0 |
| SLC15A3 | 0.105702202 | 0.02784307 | blue | 0 |
| TREML2 | 0.163780304 | 0.028046187 | blue | 0 |
| RPL27 | -0.089168836 | 0.028803536 | blue | 0 |
| LAPTM5 | 0.151186557 | 0.029025375 | blue | 0 |
| SPTAN1 | -0.161750083 | 0.029376467 | blue | 0 |
| TAGAP | 0.231571168 | 0.029651849 | blue | 0 |
| LILRA3 | 0.185063046 | 0.030371138 | blue | 0 |
| UBE2T | -0.134100288 | 0.030548433 | blue | 0 |
| HK3 | 0.190668369 | 0.030681087 | blue | 0 |
| IL6R | 0.175197623 | 0.0307194 | blue | 0 |
| USP5 | -0.130636404 | 0.030792849 | blue | 0 |
| ZZZ3 | -0.166849504 | 0.030905963 | blue | 0 |
| IPO13 | -0.164854309 | 0.030936324 | blue | 0 |
| LSP1 | 0.118184025 | 0.030962597 | blue | 0 |
| LAPTM4B | -0.201198048 | 0.03106486 | blue | 0 |
| GPBAR1 | 0.16320189 | 0.031157549 | blue | 0 |
| PI4KB | -0.073410274 | 0.031199476 | blue | 0 |
| TMEM71 | 0.210467829 | 0.031428585 | blue | 0 |
| ANKRD46 | -0.081363259 | 0.031468546 | blue | 0 |
| PLEKHO2 | 0.101118701 | 0.031543558 | blue | 0 |
| DHRS9 | 0.158821072 | 0.031660259 | blue | 0 |
| MCM2 | -0.142054833 | 0.031826826 | blue | 0 |
| SIGLEC5 | 0.203782104 | 0.032026039 | blue | 0 |
| DYSF | 0.138277352 | 0.032341044 | blue | 0 |
| LCE1C | 0.119269432 | 0.03263155 | blue | 0 |
| CD37 | 0.14070896 | 0.033022233 | blue | 0 |
| HLA-DRA | 0.210096172 | 0.03307048 | blue | 0 |
| COX2 | -0.152882995 | 0.033788686 | blue | 0 |
| FFAR2 | 0.160185838 | 0.034154784 | blue | 0 |
| POLR3F | -0.115698694 | 0.034167008 | blue | 0 |
| PSTPIP1 | 0.134174182 | 0.034588413 | blue | 0 |
| ZNF691 | -0.121973267 | 0.034682464 | blue | 0 |
| ZNF227 | -0.299344129 | 0.034768001 | blue | 0 |
| HBB | 0.392303059 | 0.03491202 | blue | 0 |
| SMCR5 | 0.116603461 | 0.034973031 | blue | 0 |
| HVCN1 | 0.130427295 | 0.035803449 | blue | 0 |
| KDELC1 | -0.157897614 | 0.036279419 | blue | 0 |
| HMGB1 | 0.101259605 | 0.036555714 | blue | 0 |
| C5orf15 | -0.081146073 | 0.036808262 | blue | 0 |
| LOC100192204 | -0.124145114 | 0.036862302 | blue | 0 |
| MPV17L2 | -0.082999704 | 0.03783045 | blue | 0 |
| RAPGEF6 | -0.131860241 | 0.038140905 | blue | 0 |
| ADRB2 | 0.120465312 | 0.038350637 | blue | 0 |
| AKAP13 | 0.126325569 | 0.038741434 | blue | 0 |
| LOC441124 | 0.166756105 | 0.03918949 | blue | 0 |
| RGS18 | 0.157342539 | 0.03939672 | blue | 0 |
| RBMX | -0.098507179 | 0.039441723 | blue | 0 |
| TP53 | -0.201728364 | 0.039503845 | blue | 0 |
| OSM | 0.118959151 | 0.039787704 | blue | 0 |
| UBAP2 | -0.136469738 | 0.04082305 | blue | 0 |
| ZBP1 | 0.130804394 | 0.041039906 | blue | 0 |
| ZNF131 | -0.106085881 | 0.041726621 | blue | 0 |
| LOC643401 | 0.197689559 | 0.042367084 | blue | 0 |
| CST7 | 0.282998519 | 0.042442611 | blue | 0 |
| RASAL3 | 0.126851525 | 0.043057346 | blue | 0 |
| CD163 | 0.255748563 | 0.043459287 | blue | 0 |
| PRF1 | 0.156383314 | 0.043875492 | blue | 0 |
| RASGRP4 | 0.150046487 | 0.044373188 | blue | 0 |
| ZNF525 | -0.165212497 | 0.044501922 | blue | 0 |
| HLA-DRB4 | 0.185086319 | 0.044540945 | blue | 0 |
| PROK2 | 0.421524203 | 0.045407212 | blue | 0 |
| GHRL | 0.126173962 | 0.046204289 | blue | 0 |
| SH2D3C | 0.133691872 | 0.046369958 | blue | 0 |
| MRS2 | -0.124660243 | 0.046692546 | blue | 0 |
| EXOC3L2 | 0.109165864 | 0.04682406 | blue | 0 |
| RNF187 | -0.087098427 | 0.047778363 | blue | 0 |
| TEX264 | -0.108106671 | 0.047932907 | blue | 0 |
| EIF4EBP1 | -0.095118476 | 0.048340833 | blue | 0 |
| C22orf43 | 0.100441008 | 0.048528475 | blue | 0 |
| SERPINB6 | -0.134723125 | 0.049144479 | blue | 0 |
| RPL13AP3 | -0.069307036 | 0.049920302 | blue | 0 |
| MMP25 | 0.214093922 | 0.004668844 | blue | 1 |
| GLT1D1 | 0.185949076 | 0.008104012 | blue | 1 |
| RGL4 | 0.208329955 | 0.011469399 | blue | 1 |
| PTPRE | 0.116858104 | 0.016371235 | blue | 1 |
| PF4 | 0.166939642 | 0.044309219 | blue | 1 |
| CSF3R | 0.15231753 | 0.022241609 | blue | 1 |
| SLC11A1 | 0.155885184 | 0.041145072 | blue | 1 |
| ITGAX | 0.201316123 | 0.017649756 | blue | 1 |
| ITGB2 | 0.251513738 | 0.00053602 | blue | 1 |
| FGR | 0.254246585 | 0.003414955 | blue | 1 |
| TYROBP | 0.261313097 | 0.000573784 | blue | 1 |
| MXD1 | 0.201949957 | 0.014799785 | blue | 2 |
| KCNJ2 | 0.156291084 | 0.039817322 | blue | 2 |
| CXCR2P1 | 0.24805605 | 0.049670582 | blue | 2 |
| CXCR1 | 0.201957941 | 0.038173377 | blue | 2 |
| AQP9 | 0.289672698 | 0.023605338 | blue | 2 |
| ALOX5AP | 0.250821629 | 0.009667473 | blue | 2 |
| LILRB2 | 0.286083972 | 0.002728785 | blue | 2 |
| B3GNT8 | 0.12876786 | 0.02462705 | blue | 3 |
| CMTM2 | 0.265762721 | 0.049966637 | blue | 3 |
| SELPLG | 0.14577773 | 0.008801681 | blue | 3 |
| S100A8 | 0.418439473 | 0.003206276 | blue | 3 |
| CD14 | 0.167903501 | 0.023573564 | blue | 3 |
| FCGR3A | 0.390223474 | 0.000765658 | blue | 3 |
| APOBR | 0.180228718 | 0.009006285 | blue | 4 |
| TREM1 | 0.173858175 | 0.046234317 | blue | 4 |
| ARHGAP9 | 0.233019606 | 0.003779173 | blue | 4 |
| GIMAP4 | 0.31087344 | 0.009764971 | blue | 4 |
| PTPN6 | 0.117453991 | 0.019054019 | blue | 5 |
| GZMH | 0.188929133 | 0.020487704 | blue | 7 |
| KCNJ15 | 0.228679926 | 0.03795798 | blue | 7 |
| RASGRP2 | 0.176680966 | 0.006690006 | blue | 7 |
| GIMAP7 | 0.194802939 | 0.030784515 | blue | 7 |
| DPEP2 | 0.180535334 | 0.036179092 | blue | 7 |
| LST1 | 0.19498133 | 0.009117794 | blue | 8 |
| C10orf54 | 0.170669468 | 0.018250169 | blue | 14 |
| NCF1 | 0.196344153 | 0.019374704 | blue | 15 |
| LILRB3 | 0.218064073 | 0.003478417 | blue | 17 |
| URB1 | -0.285568135 | 0.000368225 | brown | 0 |
| EIF3L | -0.191968309 | 0.001923088 | brown | 0 |
| SNRPN | -0.235542597 | 0.002138497 | brown | 0 |
| MRPS2 | -0.13144727 | 0.002773749 | brown | 0 |
| TXNIP | 0.332658277 | 0.002944124 | brown | 0 |
| PPP1R10 | 0.139866778 | 0.003604954 | brown | 0 |
| DUT | -0.191506 | 0.004646303 | brown | 0 |
| ANKRD32 | 0.168491396 | 0.004692186 | brown | 0 |
| CRTAP | -0.215832867 | 0.004754915 | brown | 0 |
| IDUA | -0.230583492 | 0.004847486 | brown | 0 |
| RPL18A | -0.133415326 | 0.005804127 | brown | 0 |
| DNAJC24 | -0.307906545 | 0.005937158 | brown | 0 |
| NOB1 | -0.146004853 | 0.006076632 | brown | 0 |
| PDE4DIP | -0.179659994 | 0.006677561 | brown | 0 |
| ARPC5 | 0.234869646 | 0.007242309 | brown | 0 |
| ALG10B | -0.222796196 | 0.008225014 | brown | 0 |
| HCLS1 | 0.219897584 | 0.008625488 | brown | 0 |
| RPL36 | -0.147446802 | 0.009023792 | brown | 0 |
| SLC16A6 | 0.187016131 | 0.009283321 | brown | 0 |
| EMX1 | 0.162969802 | 0.00937537 | brown | 0 |
| BAG3 | -0.159484206 | 0.009909875 | brown | 0 |
| RPL3 | -0.105785135 | 0.009911859 | brown | 0 |
| MRPL30 | -0.126554167 | 0.011868043 | brown | 0 |
| POLR2I | -0.110372395 | 0.012118909 | brown | 0 |
| PAPD7 | -0.13685853 | 0.012627178 | brown | 0 |
| MRPS11 | -0.094002225 | 0.013481273 | brown | 0 |
| EIF2D | -0.120750449 | 0.013561931 | brown | 0 |
| MRPL54 | -0.115063243 | 0.014512812 | brown | 0 |
| DOLK | -0.084425404 | 0.014638572 | brown | 0 |
| FAM86EP | -0.164143268 | 0.015472921 | brown | 0 |
| TOMM6 | -0.160518328 | 0.015517113 | brown | 0 |
| FBXL15 | -0.100891048 | 0.016133433 | brown | 0 |
| TMEM160 | -0.11370471 | 0.01618807 | brown | 0 |
| XPO4 | -0.185583308 | 0.016436513 | brown | 0 |
| LOC100652953 | -0.160479527 | 0.017678931 | brown | 0 |
| FAM86FP | -0.14264057 | 0.01779754 | brown | 0 |
| LYRM7 | -0.168170057 | 0.017881781 | brown | 0 |
| SAC3D1 | -0.083422463 | 0.017896234 | brown | 0 |
| KRT19 | 0.111492409 | 0.018285555 | brown | 0 |
| REPIN1 | -0.085640202 | 0.018880301 | brown | 0 |
| RPL13 | -0.102056362 | 0.018997396 | brown | 0 |
| MEF2C | -0.181210817 | 0.019664742 | brown | 0 |
| RALB | 0.221847888 | 0.01978513 | brown | 0 |
| RPS10P7 | -0.084151985 | 0.019864202 | brown | 0 |
| SLC7A6 | -0.218025853 | 0.020978645 | brown | 0 |
| RPL36A | -0.137429032 | 0.02162823 | brown | 0 |
| PCYT1A | 0.104272888 | 0.022010079 | brown | 0 |
| C19orf63 | -0.126042398 | 0.02221698 | brown | 0 |
| RPS27A | -0.094491343 | 0.022704538 | brown | 0 |
| FANCF | -0.152919703 | 0.023032299 | brown | 0 |
| CTDSP1 | 0.09439366 | 0.023273076 | brown | 0 |
| MRPL4 | -0.106155269 | 0.024494406 | brown | 0 |
| CIRH1A | -0.099323608 | 0.025097905 | brown | 0 |
| PELI3 | -0.174359067 | 0.025584596 | brown | 0 |
| NARS | -0.122346535 | 0.026404415 | brown | 0 |
| EIF3F | -0.130073708 | 0.026878031 | brown | 0 |
| PFDN4 | -0.140351663 | 0.027047916 | brown | 0 |
| AKAP11 | -0.170412228 | 0.027388523 | brown | 0 |
| SNRPF | -0.151622537 | 0.029431016 | brown | 0 |
| IDH3B | -0.079061526 | 0.029864941 | brown | 0 |
| NDUFA7 | -0.08326077 | 0.030220227 | brown | 0 |
| PSMC3 | -0.088949084 | 0.03128036 | brown | 0 |
| LYRM4 | -0.117334528 | 0.032655868 | brown | 0 |
| PDIK1L | -0.143094935 | 0.032663048 | brown | 0 |
| MB21D1 | 0.171326155 | 0.032959751 | brown | 0 |
| LOC152217 | -0.100688579 | 0.032967931 | brown | 0 |
| DNAJC21 | -0.158418073 | 0.033320892 | brown | 0 |
| PCCA | -0.117227543 | 0.033392155 | brown | 0 |
| MEF2A | 0.207889012 | 0.033825479 | brown | 0 |
| VEZT | -0.131564989 | 0.033956896 | brown | 0 |
| KIAA0100 | -0.074152071 | 0.034692718 | brown | 0 |
| PDCD5 | -0.146489347 | 0.034972486 | brown | 0 |
| UXT | -0.099280656 | 0.03575125 | brown | 0 |
| C20orf4 | -0.084276791 | 0.037040504 | brown | 0 |
| VASP | 0.212174946 | 0.037557218 | brown | 0 |
| ATP5G2 | -0.095361204 | 0.03852613 | brown | 0 |
| RPL24 | -0.07012013 | 0.038911795 | brown | 0 |
| NUP133 | -0.088017958 | 0.038937472 | brown | 0 |
| IFT74 | -0.187584942 | 0.039140489 | brown | 0 |
| ICMT | -0.110095241 | 0.041601457 | brown | 0 |
| C21orf59 | -0.13988293 | 0.041814089 | brown | 0 |
| PDHB | -0.092387572 | 0.042058335 | brown | 0 |
| PUF60 | -0.073366522 | 0.043420749 | brown | 0 |
| PAFAH1B3 | -0.081868535 | 0.043676703 | brown | 0 |
| MRPL40 | -0.111788787 | 0.043805748 | brown | 0 |
| C11orf1 | -0.070343741 | 0.044707609 | brown | 0 |
| THG1L | -0.083837257 | 0.044915303 | brown | 0 |
| EMBP1 | 0.165795419 | 0.046111889 | brown | 0 |
| CUTA | -0.113829486 | 0.046899601 | brown | 0 |
| ZNF25 | -0.12091972 | 0.047494852 | brown | 0 |
| STOML2 | -0.070540809 | 0.047884317 | brown | 0 |
| MRPL21 | -0.140144362 | 0.048094447 | brown | 0 |
| RPL15 | -0.09434036 | 0.048413146 | brown | 0 |
| RPL29P2 | -0.179865836 | 0.00845184 | brown | 1 |
| BTF3 | -0.116775802 | 0.010647705 | brown | 1 |
| BTF3P11 | -0.148238291 | 0.013367887 | brown | 1 |
| RPL29 | -0.109033255 | 0.043143466 | brown | 1 |
| NBEA | -0.590024665 | 0.0000392 | grey | 0 |
| PHOSPHO2 | -0.369234571 | 0.0000847 | grey | 0 |
| APBB1 | -0.340286483 | 0.000152226 | grey | 0 |
| MEPCE | -0.129040126 | 0.000188793 | grey | 0 |
| SCAND2 | -0.266773212 | 0.00020507 | grey | 0 |
| ARFGAP1 | -0.23051444 | 0.000324001 | grey | 0 |
| LCN15 | 0.261843849 | 0.000400769 | grey | 0 |
| CLN8 | -0.233828112 | 0.000500642 | grey | 0 |
| ZBTB39 | -0.520888804 | 0.000520474 | grey | 0 |
| C1orf93 | -0.22512147 | 0.000531337 | grey | 0 |
| LOC728875 | 0.14982812 | 0.000553495 | grey | 0 |
| EIF1AX | -0.246928819 | 0.000558038 | grey | 0 |
| SMC1A | -0.114020725 | 0.000616798 | grey | 0 |
| PCDH7 | 0.168071019 | 0.000635622 | grey | 0 |
| UBE2D4 | -0.163761877 | 0.000733531 | grey | 0 |
| SCHIP1 | -0.423606369 | 0.000737168 | grey | 0 |
| SYS1 | 0.14811618 | 0.000743943 | grey | 0 |
| MEX3B | -0.278867768 | 0.00074623 | grey | 0 |
| NBPF15 | 0.188179983 | 0.000750969 | grey | 0 |
| LINC00152 | 0.20570102 | 0.000882396 | grey | 0 |
| FIS1 | -0.122636717 | 0.000891211 | grey | 0 |
| C8orf46 | -0.261695959 | 0.000896354 | grey | 0 |
| ITGA11 | 0.335616097 | 0.000920433 | grey | 0 |
| INTS4 | -0.204146972 | 0.00093033 | grey | 0 |
| NEDD8 | 0.088857404 | 0.000933944 | grey | 0 |
| C2orf40 | -0.365736015 | 0.000972524 | grey | 0 |
| BTBD11 | -0.343911571 | 0.001018251 | grey | 0 |
| CUX2 | -0.285686434 | 0.001025211 | grey | 0 |
| ZFP36L2 | 0.193512725 | 0.001049772 | grey | 0 |
| METTL19 | -0.141769206 | 0.001089436 | grey | 0 |
| LOC100509814 | -0.143371783 | 0.001105843 | grey | 0 |
| ZNF671 | -0.157844529 | 0.001164773 | grey | 0 |
| CYBASC3 | -0.195934356 | 0.001180948 | grey | 0 |
| MYL5 | -0.23847578 | 0.001183045 | grey | 0 |
| EDN3 | 0.156470456 | 0.001220354 | grey | 0 |
| IRF9 | 0.20462462 | 0.001290456 | grey | 0 |
| LOC100128175 | -0.30868052 | 0.001303024 | grey | 0 |
| AVPR1A | -0.311218003 | 0.001381579 | grey | 0 |
| ZNF548 | -0.245393229 | 0.001385015 | grey | 0 |
| HEATR1 | -0.184500599 | 0.00139061 | grey | 0 |
| ZG16B | -0.392766817 | 0.001457806 | grey | 0 |
| MEGF10 | 0.253018632 | 0.001466459 | grey | 0 |
| FLJ40536 | -0.076925597 | 0.001595977 | grey | 0 |
| FAM43A | -0.326880573 | 0.001608041 | grey | 0 |
| HCN1 | 0.080558311 | 0.001628463 | grey | 0 |
| TMPRSS11F | -0.145633565 | 0.001646789 | grey | 0 |
| RPS4X | -0.183875827 | 0.001658908 | grey | 0 |
| TAP1 | 0.221648867 | 0.001666457 | grey | 0 |
| SHISA2 | 0.175832818 | 0.001677198 | grey | 0 |
| LOC100288077 | -0.074844969 | 0.001685442 | grey | 0 |
| GTF2H4 | -0.155573962 | 0.001700454 | grey | 0 |
| SNED1 | -0.206112393 | 0.001702264 | grey | 0 |
| CCNO | 0.172461006 | 0.001827271 | grey | 0 |
| PAPOLB | -0.135181353 | 0.001855462 | grey | 0 |
| CD248 | -0.301536558 | 0.001886138 | grey | 0 |
| LOC100507316 | -0.278042225 | 0.001926714 | grey | 0 |
| LOC100289341 | -0.307062351 | 0.001955006 | grey | 0 |
| YPEL4 | -0.239061352 | 0.002002675 | grey | 0 |
| KIN | 0.09789697 | 0.00200916 | grey | 0 |
| CEACAM21 | 0.391452061 | 0.002079278 | grey | 0 |
| PLAG1 | -0.261196839 | 0.002192914 | grey | 0 |
| GTF2E1 | 0.114332574 | 0.002198353 | grey | 0 |
| MAP2K5 | -0.274174404 | 0.002268649 | grey | 0 |
| CA6 | -0.192672138 | 0.002353473 | grey | 0 |
| PKIB | -0.405672051 | 0.002364945 | grey | 0 |
| FLYWCH1 | -0.196603907 | 0.002400671 | grey | 0 |
| ATG9B | -0.21827488 | 0.002462003 | grey | 0 |
| FMNL3 | -0.204929473 | 0.002496918 | grey | 0 |
| FCRLA | -0.53551426 | 0.002534618 | grey | 0 |
| ASPG | 0.338885259 | 0.002566806 | grey | 0 |
| CPEB1 | -0.31146958 | 0.002578543 | grey | 0 |
| PAIP2B | -0.23420879 | 0.002582655 | grey | 0 |
| ELL3 | -0.374589186 | 0.002617111 | grey | 0 |
| RIMS3 | -0.275889776 | 0.002626221 | grey | 0 |
| DIRAS3 | 0.313019563 | 0.002682629 | grey | 0 |
| LINC00506 | -0.135314054 | 0.002705012 | grey | 0 |
| OLIG3 | -0.430168179 | 0.00274151 | grey | 0 |
| WASH2P | 0.110932291 | 0.002900982 | grey | 0 |
| PALMD | 0.251837866 | 0.002985756 | grey | 0 |
| BDH1 | -0.13991884 | 0.002996433 | grey | 0 |
| LOC100133131 | -0.377810041 | 0.002997764 | grey | 0 |
| C14orf37 | 0.280565171 | 0.003025685 | grey | 0 |
| BEND5 | -0.289634734 | 0.00305325 | grey | 0 |
| TEKT4P2 | -0.571674886 | 0.003101858 | grey | 0 |
| PCDHA5 | 0.389326774 | 0.003104748 | grey | 0 |
| AP4S1 | -0.243631069 | 0.003117413 | grey | 0 |
| WSCD1 | 0.254005246 | 0.003131545 | grey | 0 |
| SCRN2 | -0.120836502 | 0.003137674 | grey | 0 |
| MIEN1 | 0.067959986 | 0.003148465 | grey | 0 |
| OR4K1 | 0.237280808 | 0.003161057 | grey | 0 |
| ALDH5A1 | -0.215612641 | 0.003175897 | grey | 0 |
| COL22A1 | 0.146460654 | 0.003179352 | grey | 0 |
| CCR4 | -0.320483578 | 0.003278179 | grey | 0 |
| PCBP3 | -0.358127861 | 0.003284812 | grey | 0 |
| FAM180A | 0.370997035 | 0.003287205 | grey | 0 |
| NELF | -0.163461309 | 0.003307679 | grey | 0 |
| SIDT1 | -0.331823722 | 0.003321711 | grey | 0 |
| MAZ | -0.172181383 | 0.003337181 | grey | 0 |
| BCL7A | -0.271323029 | 0.003357175 | grey | 0 |
| GNL1 | -0.147140606 | 0.003411458 | grey | 0 |
| COL4A3 | -0.269258058 | 0.003472978 | grey | 0 |
| ZNF439 | -0.279995331 | 0.003563613 | grey | 0 |
| SLC26A1 | -0.16006825 | 0.003566065 | grey | 0 |
| ZNF581 | -0.170692352 | 0.003590687 | grey | 0 |
| LOC100653227 | -0.250204753 | 0.003612055 | grey | 0 |
| CASP1 | 0.17729577 | 0.003612681 | grey | 0 |
| QRSL1 | -0.192303727 | 0.003699032 | grey | 0 |
| DTX3L | 0.227892407 | 0.003754832 | grey | 0 |
| FAM183B | -0.300047341 | 0.003765686 | grey | 0 |
| NTN1 | 0.193745491 | 0.003833982 | grey | 0 |
| ZDBF2 | -0.489539321 | 0.003838725 | grey | 0 |
| TCTN1 | -0.239959824 | 0.003850427 | grey | 0 |
| FAM104A | 0.083837561 | 0.003904301 | grey | 0 |
| PMEPA1 | -0.196449533 | 0.004016304 | grey | 0 |
| PTPRK | -0.378340094 | 0.00410846 | grey | 0 |
| PIGU | -0.175784268 | 0.004126479 | grey | 0 |
| AP2A1 | -0.221613319 | 0.004175323 | grey | 0 |
| MCF2L-AS1 | -0.284332152 | 0.004183503 | grey | 0 |
| LOC100506122 | -0.077011294 | 0.004196999 | grey | 0 |
| FBXW9 | -0.142950196 | 0.004209886 | grey | 0 |
| TSPEAR | -0.37433368 | 0.004265025 | grey | 0 |
| TUBGCP4 | -0.267950295 | 0.004267492 | grey | 0 |
| SLC18A2 | -0.33426703 | 0.004326504 | grey | 0 |
| CD274 | 0.415681333 | 0.004339254 | grey | 0 |
| RBL1 | -0.231472574 | 0.004383817 | grey | 0 |
| SYT1 | 0.275656413 | 0.004388685 | grey | 0 |
| DCAF4L2 | 0.421923897 | 0.004432522 | grey | 0 |
| PDXP | 0.12777025 | 0.004441803 | grey | 0 |
| FBXO15 | -0.441940761 | 0.00445914 | grey | 0 |
| CSF3 | 0.070188911 | 0.004487495 | grey | 0 |
| LRRC55 | 0.113797054 | 0.004497013 | grey | 0 |
| GCHFR | -0.10182716 | 0.004528658 | grey | 0 |
| ZNF250 | -0.154827437 | 0.00473875 | grey | 0 |
| LOC641518 | -0.586672964 | 0.004783513 | grey | 0 |
| KBTBD5 | 0.165764231 | 0.004880026 | grey | 0 |
| PPM1J | -0.321718679 | 0.004919102 | grey | 0 |
| BTNL8 | 0.599780071 | 0.004924315 | grey | 0 |
| SCGB3A1 | -0.683714136 | 0.004927476 | grey | 0 |
| TRIM21 | 0.168329734 | 0.004932162 | grey | 0 |
| ZFY | 0.44319182 | 0.004977322 | grey | 0 |
| ICAM4 | -0.358539385 | 0.005000492 | grey | 0 |
| AGPHD1 | -0.390284065 | 0.005005685 | grey | 0 |
| ZNF788 | 0.149879287 | 0.005024802 | grey | 0 |
| NOL6 | -0.147036008 | 0.00504709 | grey | 0 |
| IFT57 | -0.203588601 | 0.005206111 | grey | 0 |
| ZDHHC1 | 0.316104082 | 0.005207359 | grey | 0 |
| ELOVL4 | -0.276772611 | 0.005249851 | grey | 0 |
| TMED4 | -0.161630867 | 0.005290314 | grey | 0 |
| CCDC144NL | 0.485857039 | 0.00531201 | grey | 0 |
| SPRED1 | -0.352222923 | 0.005331153 | grey | 0 |
| PIAS2 | 0.262396886 | 0.005394523 | grey | 0 |
| SLC23A2 | -0.230605814 | 0.005510609 | grey | 0 |
| CD80 | -0.112940319 | 0.005512171 | grey | 0 |
| CCDC105 | 0.08703144 | 0.005584306 | grey | 0 |
| SENP5 | 0.074711905 | 0.005589423 | grey | 0 |
| ARRDC5 | -0.464879769 | 0.005609137 | grey | 0 |
| ANKMY1 | -0.24682795 | 0.005641362 | grey | 0 |
| CA5B | -0.158506802 | 0.005659945 | grey | 0 |
| C16orf5 | -0.172418364 | 0.005726388 | grey | 0 |
| C9orf16 | -0.141939745 | 0.005786217 | grey | 0 |
| CDKAL1 | -0.152903657 | 0.005818347 | grey | 0 |
| ASB9 | -0.319509063 | 0.00583144 | grey | 0 |
| ITLN1 | -0.555812744 | 0.005882656 | grey | 0 |
| CYP3A4 | 0.248641743 | 0.005902973 | grey | 0 |
| CLYBL | -0.195023399 | 0.005925441 | grey | 0 |
| ANKRD30BL | 0.208037635 | 0.005954525 | grey | 0 |
| LOC728012 | -0.104326753 | 0.005997051 | grey | 0 |
| ZBTB2 | 0.120107665 | 0.00602313 | grey | 0 |
| PHF11 | 0.170912744 | 0.006104552 | grey | 0 |
| STK31 | -0.168094759 | 0.006126852 | grey | 0 |
| CD1B | -0.362391617 | 0.006181153 | grey | 0 |
| DIP2C | -0.372000891 | 0.006182744 | grey | 0 |
| LRRN2 | 0.218492802 | 0.006186413 | grey | 0 |
| RAB8A | 0.212411012 | 0.00624791 | grey | 0 |
| LOC100507493 | -0.247950145 | 0.006259389 | grey | 0 |
| TOP3B | -0.254208326 | 0.006399049 | grey | 0 |
| SNHG11 | -0.141679441 | 0.006461236 | grey | 0 |
| C11orf80 | -0.255627516 | 0.006549183 | grey | 0 |
| TOLLIP | 0.177436999 | 0.006558168 | grey | 0 |
| UPK2 | 0.203920685 | 0.006592439 | grey | 0 |
| LOC100507487 | -0.36689301 | 0.006649591 | grey | 0 |
| COL19A1 | -0.398753614 | 0.006665174 | grey | 0 |
| GPLD1 | -0.208481684 | 0.006679509 | grey | 0 |
| RYR1 | -0.285200765 | 0.00669649 | grey | 0 |
| LOC100506667 | 0.079698183 | 0.006705923 | grey | 0 |
| ZNF544 | -0.130665868 | 0.00670821 | grey | 0 |
| CCZ1 | 0.128682753 | 0.006751529 | grey | 0 |
| SNX9 | -0.228813409 | 0.006804692 | grey | 0 |
| ZMYM6 | -0.176134957 | 0.006812711 | grey | 0 |
| GPER | 0.505956497 | 0.006821894 | grey | 0 |
| UNC119B | -0.121873996 | 0.006880005 | grey | 0 |
| FAM20B | -0.165656558 | 0.006892691 | grey | 0 |
| BSCL2 | -0.165080982 | 0.007007527 | grey | 0 |
| PSMB9 | 0.204899258 | 0.007038891 | grey | 0 |
| ISL2 | -0.488493165 | 0.007073889 | grey | 0 |
| OSCAR | 0.201118354 | 0.007134042 | grey | 0 |
| COX6A1 | 0.098639674 | 0.00714541 | grey | 0 |
| LPAL2 | 0.097416535 | 0.007178841 | grey | 0 |
| PLEKHH3 | 0.146028527 | 0.00727769 | grey | 0 |
| ZNHIT2 | -0.186733274 | 0.007415954 | grey | 0 |
| GEMIN8 | -0.166298417 | 0.007423765 | grey | 0 |
| PSMD7 | 0.084082155 | 0.007426799 | grey | 0 |
| ZNF582 | -0.408193713 | 0.00757427 | grey | 0 |
| FAM154A | -0.100475178 | 0.007590662 | grey | 0 |
| RAB7B | -0.17516605 | 0.007642216 | grey | 0 |
| DKFZP434C153 | 0.142986959 | 0.007676982 | grey | 0 |
| NUFIP1 | -0.210474379 | 0.007874387 | grey | 0 |
| DBI | 0.114005586 | 0.007882156 | grey | 0 |
| LOC100128398 | -0.215034831 | 0.007898296 | grey | 0 |
| PRRC2B | -0.135486854 | 0.007899342 | grey | 0 |
| ELK3 | -0.236648951 | 0.00790266 | grey | 0 |
| MAN1B1 | -0.176768498 | 0.007931539 | grey | 0 |
| LAYN | -0.187756454 | 0.007978718 | grey | 0 |
| TRPM4 | -0.384829946 | 0.007999812 | grey | 0 |
| MYH10 | 0.153584736 | 0.00803257 | grey | 0 |
| NDUFAF1 | 0.118243612 | 0.00818654 | grey | 0 |
| EPHA3 | 0.146524372 | 0.008228923 | grey | 0 |
| BLK | -0.354125887 | 0.008288118 | grey | 0 |
| PPP1R3F | -0.143832093 | 0.008356642 | grey | 0 |
| C1orf213 | -0.177224394 | 0.00835988 | grey | 0 |
| HSPB8 | 0.244315799 | 0.008396578 | grey | 0 |
| RALGAPA1 | -0.124974785 | 0.008427834 | grey | 0 |
| GET4 | -0.072277031 | 0.008454162 | grey | 0 |
| KRT20 | 0.238993012 | 0.008531699 | grey | 0 |
| C6orf211 | 0.100740573 | 0.008532162 | grey | 0 |
| ZNF773 | -0.152304845 | 0.008622786 | grey | 0 |
| LOC100505683 | -0.286580479 | 0.008706114 | grey | 0 |
| B3GNT4 | -0.170084264 | 0.008749478 | grey | 0 |
| ATF7IP | -0.140380768 | 0.008766 | grey | 0 |
| ACOT2 | -0.155649212 | 0.008807858 | grey | 0 |
| ANKRD54 | -0.148973789 | 0.008818579 | grey | 0 |
| POLR1E | -0.229329902 | 0.008883203 | grey | 0 |
| TNFRSF9 | 0.419186632 | 0.008886738 | grey | 0 |
| ZNF3 | -0.121150355 | 0.008941994 | grey | 0 |
| CDC14C | -0.291041789 | 0.008957274 | grey | 0 |
| TCEA3 | -0.290810456 | 0.008963033 | grey | 0 |
| KLHL36 | -0.093175192 | 0.008985354 | grey | 0 |
| ARCN1 | 0.118062643 | 0.00900072 | grey | 0 |
| BLNK | -0.411309536 | 0.009025035 | grey | 0 |
| CH25H | 0.253368277 | 0.009033113 | grey | 0 |
| IRF3 | -0.116629459 | 0.009094848 | grey | 0 |
| MAP9 | -0.291260885 | 0.009103547 | grey | 0 |
| ADAMTS4 | -0.090742893 | 0.009159631 | grey | 0 |
| ENAM | -0.077016237 | 0.009186859 | grey | 0 |
| TNIP3 | -0.224623818 | 0.009216159 | grey | 0 |
| SENP8 | -0.293167777 | 0.009216923 | grey | 0 |
| DLX4 | -0.225636961 | 0.00921892 | grey | 0 |
| CCDC124 | -0.092821205 | 0.009253064 | grey | 0 |
| DDX31 | -0.203332113 | 0.009326475 | grey | 0 |
| NFYC | 0.13710427 | 0.009347096 | grey | 0 |
| C9orf128 | -0.31844252 | 0.0093633 | grey | 0 |
| PP2672 | 0.06788714 | 0.00943628 | grey | 0 |
| AGPAT4 | -0.170106343 | 0.0094441 | grey | 0 |
| TCP10L2 | -0.271636359 | 0.009475911 | grey | 0 |
| PYCRL | -0.108195633 | 0.00947932 | grey | 0 |
| RGPD5 | -0.109047524 | 0.009504695 | grey | 0 |
| IQCK | -0.236642588 | 0.009573915 | grey | 0 |
| TSPAN13 | -0.341252627 | 0.009579915 | grey | 0 |
| ANAPC11 | 0.085761662 | 0.009645375 | grey | 0 |
| FAM86A | -0.233943391 | 0.009674446 | grey | 0 |
| RALYL | 0.147666832 | 0.009717695 | grey | 0 |
| PRMT7 | -0.160795399 | 0.009718149 | grey | 0 |
| ZNF16 | -0.261263712 | 0.009801095 | grey | 0 |
| ACSS3 | 0.25557158 | 0.009825399 | grey | 0 |
| CDCA7L | -0.184959342 | 0.009853728 | grey | 0 |
| FAM129C | -0.405264514 | 0.009933739 | grey | 0 |
| EFHB | -0.201377211 | 0.009988297 | grey | 0 |
| PNPLA4 | -0.23947473 | 0.010052958 | grey | 0 |
| CYP7A1 | -0.240985952 | 0.010129937 | grey | 0 |
| KCNH8 | -0.623593856 | 0.010246124 | grey | 0 |
| SLX4 | -0.109108381 | 0.010300508 | grey | 0 |
| FKBP7 | -0.22153164 | 0.01031384 | grey | 0 |
| SSTR3 | 0.319207735 | 0.010324996 | grey | 0 |
| DHX36 | -0.086295807 | 0.010518932 | grey | 0 |
| FBLN5 | -0.286098605 | 0.010522616 | grey | 0 |
| GATA5 | 0.233998274 | 0.010526757 | grey | 0 |
| IGLON5 | 0.292304085 | 0.010554615 | grey | 0 |
| POTEG | 0.179276745 | 0.010624701 | grey | 0 |
| FOXP2 | 0.079930618 | 0.010635589 | grey | 0 |
| USP47 | -0.111071025 | 0.010664646 | grey | 0 |
| KCTD8 | 0.171230659 | 0.010689797 | grey | 0 |
| GTF2IRD2B | -0.125200604 | 0.01076921 | grey | 0 |
| CBLC | -0.059593716 | 0.010787937 | grey | 0 |
| GNA12 | -0.124147005 | 0.010867109 | grey | 0 |
| MCRS1 | -0.081926393 | 0.010881712 | grey | 0 |
| C9orf46 | 0.126218444 | 0.010922431 | grey | 0 |
| ARHGEF4 | 0.132153434 | 0.010986327 | grey | 0 |
| HTR7P1 | -0.212947098 | 0.011162711 | grey | 0 |
| MOB2 | -0.09696595 | 0.011217143 | grey | 0 |
| LOC100131096 | -0.216394066 | 0.01126986 | grey | 0 |
| ACOT7 | -0.134651396 | 0.011329627 | grey | 0 |
| SIRPD | 0.32325555 | 0.011527336 | grey | 0 |
| ITGA9 | -0.184130701 | 0.011562537 | grey | 0 |
| MAPK11 | -0.118211126 | 0.011606369 | grey | 0 |
| BNC1 | 0.237462868 | 0.011688578 | grey | 0 |
| C17orf108 | -0.251370989 | 0.011755591 | grey | 0 |
| CHML | -0.183480388 | 0.011767804 | grey | 0 |
| GOLT1B | -0.197479609 | 0.011882972 | grey | 0 |
| N4BP3 | -0.125027794 | 0.011884498 | grey | 0 |
| ZNF280C | -0.198467481 | 0.011902384 | grey | 0 |
| CAPS | -0.17138771 | 0.011927696 | grey | 0 |
| EAPP | 0.095181713 | 0.011933787 | grey | 0 |
| ZNF804B | -0.050898848 | 0.011977947 | grey | 0 |
| STK33 | -0.271929841 | 0.012150372 | grey | 0 |
| DNASE1L3 | -0.483785929 | 0.01219514 | grey | 0 |
| B3GAT3 | -0.123096414 | 0.012239931 | grey | 0 |
| MSTO1 | -0.217145259 | 0.012263148 | grey | 0 |
| GRASP | -0.200340655 | 0.012271179 | grey | 0 |
| IRF5 | 0.221387719 | 0.012297678 | grey | 0 |
| OTUD4 | -0.149055232 | 0.012384388 | grey | 0 |
| ANKRD5 | -0.266612616 | 0.01241004 | grey | 0 |
| TDRD10 | 0.262445218 | 0.012421919 | grey | 0 |
| PAWR | -0.255238518 | 0.012474957 | grey | 0 |
| LOC100287590 | -0.193244707 | 0.01265325 | grey | 0 |
| LY6H | 0.136924149 | 0.012681868 | grey | 0 |
| ATG4B | -0.088165856 | 0.012726776 | grey | 0 |
| DDI2 | -0.175747053 | 0.01273078 | grey | 0 |
| TUT1 | -0.244037482 | 0.01275334 | grey | 0 |
| ZNF382 | -0.375152907 | 0.012857111 | grey | 0 |
| UG0898H09 | 0.096402806 | 0.01292775 | grey | 0 |
| AGPAT4-IT1 | -0.204480754 | 0.012943003 | grey | 0 |
| ZNF219 | -0.124163658 | 0.012947084 | grey | 0 |
| SIRT3 | -0.097638902 | 0.012990228 | grey | 0 |
| NPFFR1 | 0.140982036 | 0.0130125 | grey | 0 |
| ACSM1 | -0.349358201 | 0.013072329 | grey | 0 |
| KCNJ3 | 0.243409608 | 0.013088357 | grey | 0 |
| GSTM5 | 0.172857024 | 0.013195937 | grey | 0 |
| NDRG2 | -0.161591601 | 0.013213652 | grey | 0 |
| MCF2L | -0.127285785 | 0.013215533 | grey | 0 |
| FAM41C | 0.117959397 | 0.01323172 | grey | 0 |
| AKAP17A | 0.127943405 | 0.013269677 | grey | 0 |
| CD22 | -0.434249052 | 0.013300332 | grey | 0 |
| GOLGA6L10 | -0.152522528 | 0.013310003 | grey | 0 |
| RPS3 | -0.152554636 | 0.013330975 | grey | 0 |
| ATRIP | -0.135356003 | 0.013502164 | grey | 0 |
| FAM86B2 | -0.120840454 | 0.013522705 | grey | 0 |
| ACMSD | 0.13039359 | 0.013579394 | grey | 0 |
| DSCR6 | 0.201451632 | 0.01360332 | grey | 0 |
| FZD3 | -0.263294678 | 0.013620636 | grey | 0 |
| MMP12 | 0.170292874 | 0.013653804 | grey | 0 |
| NCAM1 | 0.127537141 | 0.013701594 | grey | 0 |
| NQO1 | 0.34890364 | 0.013736127 | grey | 0 |
| USP32P2 | 0.228539847 | 0.013836348 | grey | 0 |
| PSD3 | -0.171325978 | 0.01384978 | grey | 0 |
| IYD | -0.23348415 | 0.01390423 | grey | 0 |
| RPL10 | -0.101932203 | 0.014237334 | grey | 0 |
| FLRT2 | 0.191643536 | 0.014263635 | grey | 0 |
| RPS6KB1 | -0.087324963 | 0.01426393 | grey | 0 |
| SNX6 | 0.125001728 | 0.014351129 | grey | 0 |
| PINX1 | -0.160734137 | 0.014397269 | grey | 0 |
| LOC100132319 | 0.246344856 | 0.014411249 | grey | 0 |
| CREG1 | 0.148832405 | 0.014411529 | grey | 0 |
| C10orf95 | -0.268749299 | 0.014434399 | grey | 0 |
| REEP1 | -0.338099696 | 0.014436721 | grey | 0 |
| FOXL1 | 0.285135861 | 0.014445922 | grey | 0 |
| C11orf45 | -0.256529546 | 0.014452531 | grey | 0 |
| BCORP1 | 0.133370159 | 0.014474882 | grey | 0 |
| CLRN1 | 0.074309189 | 0.014527876 | grey | 0 |
| DPPA4 | -0.283771647 | 0.014571376 | grey | 0 |
| LOC442421 | -0.099837638 | 0.014661933 | grey | 0 |
| C20orf103 | -0.304240035 | 0.014669302 | grey | 0 |
| GAN | -0.272760248 | 0.014686849 | grey | 0 |
| SMTN | -0.219840943 | 0.014743873 | grey | 0 |
| LOC284933 | 0.20078867 | 0.014749106 | grey | 0 |
| YDJC | -0.067119773 | 0.014782331 | grey | 0 |
| LOC643355 | -0.330287098 | 0.014791264 | grey | 0 |
| GINS1 | 0.242927153 | 0.014802485 | grey | 0 |
| TOP1MT | -0.217175096 | 0.014807372 | grey | 0 |
| ZNF215 | 0.190980265 | 0.014813285 | grey | 0 |
| AP2M1 | -0.127905492 | 0.014846171 | grey | 0 |
| VSIG1 | -0.509462163 | 0.014855645 | grey | 0 |
| ZNF681 | -0.190126019 | 0.014914073 | grey | 0 |
| PCDHGB1 | -0.095136879 | 0.01497446 | grey | 0 |
| PMS2L2 | -0.099270837 | 0.015026497 | grey | 0 |
| LOC100287216 | -0.132536259 | 0.015034404 | grey | 0 |
| FGF21 | 0.225869219 | 0.015038583 | grey | 0 |
| CCDC144A | 0.233298094 | 0.01515565 | grey | 0 |
| SPRY3 | 0.157333793 | 0.01516516 | grey | 0 |
| CUL3 | -0.277828626 | 0.015233106 | grey | 0 |
| CRYM | -0.362375946 | 0.015241261 | grey | 0 |
| ZNF546 | -0.192718351 | 0.015241847 | grey | 0 |
| COL9A3 | 0.438663143 | 0.015245603 | grey | 0 |
| LOC100128893 | 0.274216456 | 0.015298272 | grey | 0 |
| NECAB1 | 0.220367823 | 0.015358798 | grey | 0 |
| TCF4 | -0.150449856 | 0.015425573 | grey | 0 |
| EGLN3 | -0.271903416 | 0.015433439 | grey | 0 |
| WFDC10B | 0.088085961 | 0.015576239 | grey | 0 |
| CEP63 | 0.178672711 | 0.015581232 | grey | 0 |
| ZNF167 | 0.200074294 | 0.015598956 | grey | 0 |
| CD40LG | -0.449591751 | 0.015657104 | grey | 0 |
| ATP9B | -0.192608091 | 0.01569985 | grey | 0 |
| SYNE2 | 0.171760526 | 0.015732785 | grey | 0 |
| RUVBL2 | -0.083137101 | 0.015809032 | grey | 0 |
| INSM1 | 0.228759235 | 0.015869929 | grey | 0 |
| CHST9-AS1 | -0.116307232 | 0.015883746 | grey | 0 |
| ZNF285 | -0.42497041 | 0.015927998 | grey | 0 |
| UBE2A | 0.06319896 | 0.015954776 | grey | 0 |
| C21orf2 | -0.210532658 | 0.015982683 | grey | 0 |
| NOL4 | 0.05156784 | 0.016165288 | grey | 0 |
| FAM176A | 0.223638815 | 0.016216405 | grey | 0 |
| CHGA | 0.208528032 | 0.016337772 | grey | 0 |
| LOC100507280 | -0.307077228 | 0.016339637 | grey | 0 |
| TRIM34 | 0.132617377 | 0.016355957 | grey | 0 |
| KRT6C | 0.131874094 | 0.016376153 | grey | 0 |
| CA5BP1 | -0.332818355 | 0.01644775 | grey | 0 |
| PSMB1 | 0.068990321 | 0.016489016 | grey | 0 |
| LGR5 | 0.205800741 | 0.016494828 | grey | 0 |
| CXorf21 | 0.285811426 | 0.016516078 | grey | 0 |
| ENG | -0.157439494 | 0.016530326 | grey | 0 |
| C8orf38 | -0.229790828 | 0.016623927 | grey | 0 |
| EPHB6 | -0.159710336 | 0.016657798 | grey | 0 |
| SCARA5 | -0.165352948 | 0.016787793 | grey | 0 |
| ATP4A | -0.197217194 | 0.016822709 | grey | 0 |
| BEX5 | -0.276348136 | 0.016840461 | grey | 0 |
| SPANXA2-OT1 | -0.346095098 | 0.016842803 | grey | 0 |
| ZC3H12D | -0.26894729 | 0.016846888 | grey | 0 |
| LYZL1 | 0.361906211 | 0.016862583 | grey | 0 |
| FCRL1 | -0.405987677 | 0.016899658 | grey | 0 |
| PNMT | 0.399330525 | 0.016916931 | grey | 0 |
| CMBL | 0.289166496 | 0.016977181 | grey | 0 |
| RNF38 | 0.167505285 | 0.01697948 | grey | 0 |
| UGT1A8 | 0.092102823 | 0.017016817 | grey | 0 |
| ITPR1 | -0.108898368 | 0.017022809 | grey | 0 |
| FMR1 | 0.101849855 | 0.017058998 | grey | 0 |
| CCDC166 | -0.375426036 | 0.017067603 | grey | 0 |
| FAM40A | -0.071362242 | 0.017106752 | grey | 0 |
| EPHB2 | 0.356869447 | 0.017176279 | grey | 0 |
| SLC15A1 | 0.093599963 | 0.017301366 | grey | 0 |
| C16orf54 | 0.278736819 | 0.017337441 | grey | 0 |
| TBC1D25 | -0.304275311 | 0.017367608 | grey | 0 |
| CAT | 0.212540009 | 0.01739511 | grey | 0 |
| CHTF8 | -0.105571373 | 0.017431561 | grey | 0 |
| C1orf192 | -0.043703906 | 0.017549037 | grey | 0 |
| NANOS3 | 0.274817296 | 0.017628547 | grey | 0 |
| FOXG1 | 0.129746701 | 0.017743983 | grey | 0 |
| ATPAF1 | -0.1724221 | 0.017792179 | grey | 0 |
| L3MBTL2 | -0.17136138 | 0.017800246 | grey | 0 |
| NECAP1 | 0.166485014 | 0.017837783 | grey | 0 |
| ZNF709 | -0.17523209 | 0.017876186 | grey | 0 |
| CYP46A1 | 0.236504486 | 0.017896651 | grey | 0 |
| ZNF92 | -0.102466835 | 0.017901878 | grey | 0 |
| TRPC1 | -0.303826967 | 0.018192517 | grey | 0 |
| EFTUD1 | 0.16966342 | 0.018219958 | grey | 0 |
| TMPRSS11D | -0.104106246 | 0.01823476 | grey | 0 |
| SNAP29 | -0.149420443 | 0.018242894 | grey | 0 |
| OR7G3 | 0.07376948 | 0.018262272 | grey | 0 |
| C14orf45 | -0.342971755 | 0.018298525 | grey | 0 |
| ELK4 | -0.218115122 | 0.018426865 | grey | 0 |
| CD180 | -0.566028262 | 0.018481233 | grey | 0 |
| NF2 | -0.137901431 | 0.018493949 | grey | 0 |
| MDH1B | 0.141311327 | 0.018506195 | grey | 0 |
| KCNK16 | -0.14141591 | 0.018506395 | grey | 0 |
| PRDX2 | -0.110019718 | 0.018540168 | grey | 0 |
| FBXO6 | 0.156830146 | 0.018557654 | grey | 0 |
| ADAMTS19 | 0.21664229 | 0.018620021 | grey | 0 |
| HSDL1 | -0.235760769 | 0.018645494 | grey | 0 |
| GNG13 | 0.404753302 | 0.018688529 | grey | 0 |
| SAMD9L | 0.245858802 | 0.018753818 | grey | 0 |
| MGC3771 | -0.360240546 | 0.018762891 | grey | 0 |
| PASK | -0.250323246 | 0.018778672 | grey | 0 |
| KANK4 | 0.192504949 | 0.018813659 | grey | 0 |
| SP3 | 0.10189448 | 0.018846204 | grey | 0 |
| USF1 | 0.324206464 | 0.019045375 | grey | 0 |
| LOC100506262 | -0.387180624 | 0.019047571 | grey | 0 |
| ERCC2 | -0.093846983 | 0.019049176 | grey | 0 |
| ZYG11A | 0.194478042 | 0.019059438 | grey | 0 |
| STAP1 | -0.585371317 | 0.019064726 | grey | 0 |
| STAG3 | -0.133491628 | 0.019101038 | grey | 0 |
| FP588 | -0.258782488 | 0.019113166 | grey | 0 |
| GLRA2 | 0.06313732 | 0.019169121 | grey | 0 |
| HRH1 | -0.188146134 | 0.019182891 | grey | 0 |
| LOC440104 | -0.152455976 | 0.019226285 | grey | 0 |
| C17orf64 | 0.150973946 | 0.01926315 | grey | 0 |
| C21orf91 | 0.100563069 | 0.019267203 | grey | 0 |
| FAM82B | -0.078635367 | 0.019267995 | grey | 0 |
| BEND3 | -0.147549218 | 0.019288413 | grey | 0 |
| SMAD1 | -0.196282537 | 0.019394962 | grey | 0 |
| NR3C2 | -0.430291659 | 0.019472884 | grey | 0 |
| NR1I2 | 0.250713637 | 0.019516438 | grey | 0 |
| GAMT | -0.096686001 | 0.019539354 | grey | 0 |
| LOC285141 | 0.251074027 | 0.01957661 | grey | 0 |
| ORC1 | -0.278484935 | 0.019582731 | grey | 0 |
| PPEF1 | -0.349497073 | 0.019585074 | grey | 0 |
| C16orf62 | -0.149615471 | 0.019621495 | grey | 0 |
| CEP44 | -0.171273386 | 0.019639329 | grey | 0 |
| TRDMT1 | -0.164637545 | 0.019641221 | grey | 0 |
| ATF7 | 0.119784564 | 0.019684515 | grey | 0 |
| PLEKHA3 | 0.142727076 | 0.019712084 | grey | 0 |
| TMEM163 | -0.229249875 | 0.019722657 | grey | 0 |
| CCDC66 | -0.116710408 | 0.019735642 | grey | 0 |
| LOC100129794 | 0.248677637 | 0.019750517 | grey | 0 |
| NOM1 | -0.221049065 | 0.019771711 | grey | 0 |
| MAB21L1 | -0.106128658 | 0.019789728 | grey | 0 |
| ASB16 | 0.153230795 | 0.01979013 | grey | 0 |
| UPK1B | 0.058504438 | 0.019823638 | grey | 0 |
| UGT2B10 | 0.103653364 | 0.01995663 | grey | 0 |
| LOC100127972 | -0.297366551 | 0.019959651 | grey | 0 |
| AGBL5 | -0.127333771 | 0.019974809 | grey | 0 |
| SPRR3 | 0.291396882 | 0.020106644 | grey | 0 |
| PNLIPRP2 | 0.22815251 | 0.020132467 | grey | 0 |
| PRPF40A | 0.087247169 | 0.020135852 | grey | 0 |
| DFNB59 | -0.30333896 | 0.020141636 | grey | 0 |
| FIZ1 | -0.157084369 | 0.020174171 | grey | 0 |
| APOF | -0.183865803 | 0.020227959 | grey | 0 |
| DSG1 | -0.037444585 | 0.020247384 | grey | 0 |
| LOC100506651 | -0.166001998 | 0.020254362 | grey | 0 |
| LOC100128775 | 0.073596515 | 0.020261279 | grey | 0 |
| CATSPER3 | -0.295735474 | 0.020262187 | grey | 0 |
| LONRF2 | 0.209559356 | 0.020305461 | grey | 0 |
| MEI1 | 0.095507123 | 0.020383638 | grey | 0 |
| LOC100507311 | 0.237673586 | 0.020385719 | grey | 0 |
| NR3C1 | 0.074068596 | 0.020395265 | grey | 0 |
| MTERFD3 | 0.162888086 | 0.020412789 | grey | 0 |
| DDX50 | -0.15386363 | 0.020427217 | grey | 0 |
| SNX20 | 0.15792858 | 0.020476707 | grey | 0 |
| TBC1D30 | -0.087106699 | 0.020477759 | grey | 0 |
| KIAA1671 | 0.153105492 | 0.020563921 | grey | 0 |
| MOK | -0.239945206 | 0.020566794 | grey | 0 |
| MAN1C1 | -0.269106231 | 0.020569186 | grey | 0 |
| LOC100505622 | -0.501008455 | 0.020591021 | grey | 0 |
| SCGB1D2 | -0.069727437 | 0.020620174 | grey | 0 |
| SLITRK4 | -0.17632181 | 0.020785513 | grey | 0 |
| C14orf23 | -0.04322846 | 0.020933073 | grey | 0 |
| HNRNPL | 0.130587926 | 0.020953014 | grey | 0 |
| C12orf10 | -0.082941282 | 0.021011966 | grey | 0 |
| KLK9 | 0.137297696 | 0.021033281 | grey | 0 |
| AMPD3 | 0.137137337 | 0.021075321 | grey | 0 |
| CD101 | -0.506645171 | 0.021092683 | grey | 0 |
| LIPE | -0.121636434 | 0.021140884 | grey | 0 |
| SLC25A30 | -0.185803226 | 0.021291428 | grey | 0 |
| EXD3 | -0.241104016 | 0.021297573 | grey | 0 |
| FAM55D | 0.113934689 | 0.021314788 | grey | 0 |
| SNHG13 | -0.152557037 | 0.021401963 | grey | 0 |
| PDHA2 | -0.074435692 | 0.021452289 | grey | 0 |
| USP13 | -0.218046816 | 0.021464636 | grey | 0 |
| AUP1 | -0.091962508 | 0.021482644 | grey | 0 |
| TBKBP1 | 0.212685496 | 0.021498143 | grey | 0 |
| GSX1 | 0.290342023 | 0.021501822 | grey | 0 |
| C17orf110 | -0.318134952 | 0.021508213 | grey | 0 |
| TM6SF1 | 0.31351362 | 0.021527481 | grey | 0 |
| SPINK2 | -0.491133004 | 0.021606913 | grey | 0 |
| RHBDF2 | 0.176603198 | 0.021620459 | grey | 0 |
| DNAJB7 | -0.077661397 | 0.02163215 | grey | 0 |
| GPR68 | -0.223599117 | 0.021749229 | grey | 0 |
| ACRC | 0.129091806 | 0.021763598 | grey | 0 |
| OGFOD2 | -0.14560269 | 0.021764678 | grey | 0 |
| C9orf24 | -0.302210474 | 0.021787718 | grey | 0 |
| ZNF562 | -0.075152758 | 0.021796878 | grey | 0 |
| PPP1R3E | -0.183375292 | 0.02190855 | grey | 0 |
| TRAF3IP2 | -0.138266521 | 0.021978383 | grey | 0 |
| TMEM129 | -0.09802971 | 0.0220556 | grey | 0 |
| LOC283663 | -0.324351053 | 0.022092702 | grey | 0 |
| LRGUK | 0.184181802 | 0.022103741 | grey | 0 |
| TPM2 | -0.089946017 | 0.022387413 | grey | 0 |
| TSPAN33 | -0.275235044 | 0.022455809 | grey | 0 |
| SLC27A6 | 0.199801236 | 0.022513729 | grey | 0 |
| KIAA0125 | -0.336893194 | 0.022527095 | grey | 0 |
| C5orf24 | -0.162754892 | 0.022592978 | grey | 0 |
| PRO1596 | -0.043773745 | 0.02271203 | grey | 0 |
| WIPF3 | 0.375862728 | 0.022750555 | grey | 0 |
| H2AFY2 | -0.235735554 | 0.02284528 | grey | 0 |
| LOC100130428 | -0.313417921 | 0.022854727 | grey | 0 |
| CENPQ | 0.144306079 | 0.022858398 | grey | 0 |
| B4GALT7 | -0.131950065 | 0.02288376 | grey | 0 |
| EDAR | -0.291138606 | 0.022919976 | grey | 0 |
| SYT17 | -0.363199722 | 0.02293609 | grey | 0 |
| ZNF703 | 0.298148548 | 0.023059131 | grey | 0 |
| LRRC58 | -0.152439848 | 0.023073209 | grey | 0 |
| SNHG5 | 0.293750084 | 0.023160524 | grey | 0 |
| SCG3 | 0.179138848 | 0.023202461 | grey | 0 |
| KLRB1 | 0.295246303 | 0.02324072 | grey | 0 |
| IFT140 | -0.183692244 | 0.023414133 | grey | 0 |
| MIR17HG | -0.181167331 | 0.023415517 | grey | 0 |
| WDR72 | 0.122397048 | 0.023452374 | grey | 0 |
| LYPD2 | -0.482511404 | 0.023511312 | grey | 0 |
| MBD3 | -0.133686196 | 0.023520786 | grey | 0 |
| LOC401431 | -0.274523051 | 0.023566415 | grey | 0 |
| IL21 | 0.155054797 | 0.023577333 | grey | 0 |
| KCNN1 | 0.177016763 | 0.023581276 | grey | 0 |
| SNW1 | 0.056864459 | 0.023646073 | grey | 0 |
| PAPSS2 | -0.229898614 | 0.023668444 | grey | 0 |
| CBX5 | -0.116650916 | 0.023676755 | grey | 0 |
| TFCP2L1 | -0.411688362 | 0.023742942 | grey | 0 |
| ZIM2 | 0.245025791 | 0.023846318 | grey | 0 |
| ACOX3 | -0.155114115 | 0.023946587 | grey | 0 |
| KIAA1609 | 0.123361815 | 0.023950285 | grey | 0 |
| SLC9A9 | -0.154641148 | 0.024026563 | grey | 0 |
| GPRC5D | 0.205865319 | 0.024167941 | grey | 0 |
| HMGCL | -0.080799542 | 0.024174087 | grey | 0 |
| SPNS1 | -0.179788298 | 0.02421544 | grey | 0 |
| ACAN | 0.303588841 | 0.024230972 | grey | 0 |
| PHC1 | -0.246071987 | 0.024274098 | grey | 0 |
| MECR | -0.135759318 | 0.024319498 | grey | 0 |
| GUCA1B | -0.233826522 | 0.024364785 | grey | 0 |
| USP17 | 0.375410778 | 0.024407338 | grey | 0 |
| PFKM | -0.079810132 | 0.024429124 | grey | 0 |
| ACHE | 0.292976401 | 0.024467247 | grey | 0 |
| PCSK7 | -0.139831516 | 0.024535681 | grey | 0 |
| CENPI | 0.186430142 | 0.024611203 | grey | 0 |
| LOC100506113 | 0.268985867 | 0.024822895 | grey | 0 |
| SLC36A3 | 0.262919966 | 0.024894946 | grey | 0 |
| GPX7 | -0.147808505 | 0.024914022 | grey | 0 |
| CD300C | 0.208487461 | 0.024960909 | grey | 0 |
| MACROD1 | 0.144544078 | 0.02500034 | grey | 0 |
| LOC100129935 | -0.177890129 | 0.025016868 | grey | 0 |
| HOXC13 | -0.176149323 | 0.025023205 | grey | 0 |
| FBXL21 | 0.192394242 | 0.025065825 | grey | 0 |
| ZNF843 | 0.176991736 | 0.025107686 | grey | 0 |
| ZNF778 | -0.272760112 | 0.025115084 | grey | 0 |
| UBR4 | -0.146775425 | 0.025120257 | grey | 0 |
| FLJ34690 | 0.152310939 | 0.025159363 | grey | 0 |
| SGSH | -0.155452486 | 0.025160237 | grey | 0 |
| HP1BP3 | 0.072333885 | 0.025180992 | grey | 0 |
| HAUS7 | -0.132856591 | 0.025207628 | grey | 0 |
| AHRR | 0.20922609 | 0.02525876 | grey | 0 |
| SNHG7 | -0.105931742 | 0.025328763 | grey | 0 |
| GABRD | 0.230839472 | 0.025360502 | grey | 0 |
| FABP3 | -0.151331343 | 0.025431805 | grey | 0 |
| ZNF684 | 0.291709376 | 0.025467479 | grey | 0 |
| YBEY | -0.204186565 | 0.025526116 | grey | 0 |
| TMEM64 | -0.127980459 | 0.02553073 | grey | 0 |
| EHMT2 | -0.115552953 | 0.025538232 | grey | 0 |
| DPM1 | 0.091329755 | 0.025578606 | grey | 0 |
| PRKG2 | -0.091077613 | 0.025623764 | grey | 0 |
| NTN5 | -0.17616486 | 0.025639572 | grey | 0 |
| DHCR7 | -0.085307109 | 0.025694238 | grey | 0 |
| MGC2752 | -0.12521717 | 0.025707177 | grey | 0 |
| SLC2A11 | -0.158774008 | 0.025781252 | grey | 0 |
| LOC100509121 | 0.374433303 | 0.025783099 | grey | 0 |
| CNDP1 | 0.28595928 | 0.025805297 | grey | 0 |
| SOCS4 | -0.24406585 | 0.025815317 | grey | 0 |
| ATP13A2 | -0.112826827 | 0.025840226 | grey | 0 |
| S1PR3 | -0.304956346 | 0.025879148 | grey | 0 |
| FAM190A | -0.05252651 | 0.025881961 | grey | 0 |
| LIPG | 0.168614415 | 0.02590774 | grey | 0 |
| LOC100129447 | -0.301463085 | 0.025933057 | grey | 0 |
| HIST1H3G | 0.128325173 | 0.025970363 | grey | 0 |
| FLJ10661 | -0.154589346 | 0.025972542 | grey | 0 |
| LOC80054 | -0.355019011 | 0.025980011 | grey | 0 |
| OR2L13 | -0.109761059 | 0.026027818 | grey | 0 |
| PRSS57 | 0.340680276 | 0.026099417 | grey | 0 |
| C5orf54 | -0.2012955 | 0.026219143 | grey | 0 |
| HRSP12 | -0.107879752 | 0.026260429 | grey | 0 |
| ZNF767 | -0.139149172 | 0.026270647 | grey | 0 |
| GYPE | -0.285811642 | 0.026346174 | grey | 0 |
| CAMK2B | 0.20589483 | 0.026372255 | grey | 0 |
| ING5 | -0.111572527 | 0.026403683 | grey | 0 |
| MTSS1L | 0.113291292 | 0.026403705 | grey | 0 |
| KRT19P2 | 0.216396732 | 0.026425932 | grey | 0 |
| PCLO | 0.109306986 | 0.02647462 | grey | 0 |
| C19orf20 | -0.348430929 | 0.026478033 | grey | 0 |
| KLRG2 | 0.190594272 | 0.026494783 | grey | 0 |
| KLHDC3 | -0.101245132 | 0.026541767 | grey | 0 |
| FAM164A | -0.238144727 | 0.026600515 | grey | 0 |
| TGIF2 | -0.191011054 | 0.026606221 | grey | 0 |
| DSC1 | -0.560190307 | 0.026612651 | grey | 0 |
| OSBPL10 | -0.314232199 | 0.026620405 | grey | 0 |
| CAMTA1 | -0.171673854 | 0.026683188 | grey | 0 |
| UTY | 0.635926052 | 0.026726457 | grey | 0 |
| FAM161B | -0.319016311 | 0.026732276 | grey | 0 |
| RPS9 | -0.095745424 | 0.026734561 | grey | 0 |
| OR4C15 | 0.342469704 | 0.026788499 | grey | 0 |
| ACSL6 | -0.125241836 | 0.026806869 | grey | 0 |
| ANKRD26 | -0.16786969 | 0.026856675 | grey | 0 |
| C12orf44 | -0.093000595 | 0.026904752 | grey | 0 |
| GSPT1 | -0.171208146 | 0.026939469 | grey | 0 |
| C11orf36 | -0.190993081 | 0.027187375 | grey | 0 |
| C18orf1 | -0.096521378 | 0.027206711 | grey | 0 |
| PTGES3 | 0.105942613 | 0.027224187 | grey | 0 |
| OGT | 0.108897778 | 0.027227942 | grey | 0 |
| CENPT | -0.167108692 | 0.027301595 | grey | 0 |
| SALL1 | 0.205386756 | 0.027302701 | grey | 0 |
| LOC100128252 | -0.260281631 | 0.027394576 | grey | 0 |
| MAPRE3 | -0.235177792 | 0.027436652 | grey | 0 |
| TMEM39B | -0.072101237 | 0.027478492 | grey | 0 |
| CD72 | -0.413878372 | 0.027486182 | grey | 0 |
| DPY19L2P4 | -0.261165476 | 0.027490588 | grey | 0 |
| SALL2 | -0.110513294 | 0.027593326 | grey | 0 |
| CNKSR3 | 0.211823454 | 0.027699843 | grey | 0 |
| ASF1A | -0.207996159 | 0.027762831 | grey | 0 |
| DENND1B | 0.092814155 | 0.027785527 | grey | 0 |
| GPR142 | 0.296876509 | 0.027817891 | grey | 0 |
| LOC100289079 | -0.231088743 | 0.027843114 | grey | 0 |
| DERL1 | 0.077092009 | 0.027867803 | grey | 0 |
| LMO2 | 0.135370876 | 0.027872276 | grey | 0 |
| SOX14 | 0.073022046 | 0.027879619 | grey | 0 |
| LOC100132014 | -0.066216834 | 0.027926579 | grey | 0 |
| PDK2 | -0.089867126 | 0.028028519 | grey | 0 |
| TM7SF2 | -0.127600014 | 0.028163359 | grey | 0 |
| SERPINA4 | 0.170619729 | 0.028242643 | grey | 0 |
| USP49 | -0.267277447 | 0.02827138 | grey | 0 |
| ZC3HAV1L | -0.150334454 | 0.028369083 | grey | 0 |
| ZNF91 | -0.13487554 | 0.02838679 | grey | 0 |
| ISOC1 | -0.140564114 | 0.028414647 | grey | 0 |
| ITGBL1 | 0.193389785 | 0.02851308 | grey | 0 |
| TCL6 | -0.100324002 | 0.02852135 | grey | 0 |
| RTN4RL1 | -0.194725283 | 0.028528466 | grey | 0 |
| ADAM7 | 0.209873786 | 0.02854638 | grey | 0 |
| PIN1 | -0.078481395 | 0.028553701 | grey | 0 |
| CHMP6 | -0.083165949 | 0.028562529 | grey | 0 |
| ANKRD49 | 0.059005747 | 0.028577579 | grey | 0 |
| FAM167A | -0.225836154 | 0.028615354 | grey | 0 |
| RIC8B | -0.22758398 | 0.028679383 | grey | 0 |
| PNPLA8 | -0.136495567 | 0.028693135 | grey | 0 |
| NT5E | -0.147722305 | 0.028750728 | grey | 0 |
| DNAH5 | 0.08248462 | 0.028815362 | grey | 0 |
| TMEM53 | -0.101604085 | 0.028883593 | grey | 0 |
| FAM159A | -0.306636124 | 0.028923449 | grey | 0 |
| PCBD2 | -0.160778314 | 0.028928043 | grey | 0 |
| ZEB2 | 0.122980553 | 0.028936888 | grey | 0 |
| XPNPEP3 | -0.135132043 | 0.029065166 | grey | 0 |
| TCEAL7 | 0.075958216 | 0.029098354 | grey | 0 |
| NCAPD3 | -0.05454871 | 0.029134175 | grey | 0 |
| ADCY3 | -0.137227603 | 0.029151358 | grey | 0 |
| GRIP1 | -0.247552469 | 0.029217277 | grey | 0 |
| PSG3 | -0.049592797 | 0.029241204 | grey | 0 |
| HKR1 | -0.192352947 | 0.029251082 | grey | 0 |
| DBIL5P | -0.292151628 | 0.029374507 | grey | 0 |
| THEM4 | -0.178650111 | 0.029439042 | grey | 0 |
| TDRD7 | 0.160758707 | 0.029450572 | grey | 0 |
| HSFY2 | -0.044605659 | 0.029460228 | grey | 0 |
| AP1S2 | -0.180717257 | 0.029475056 | grey | 0 |
| LOC389906 | -0.131648386 | 0.02953139 | grey | 0 |
| LOC100507377 | 0.147241436 | 0.029558786 | grey | 0 |
| CXorf59 | -0.068350523 | 0.029707281 | grey | 0 |
| DFNA5 | -0.311037841 | 0.029724553 | grey | 0 |
| PCDHA9 | -0.105579795 | 0.029757507 | grey | 0 |
| TRMT61B | -0.284521929 | 0.029858703 | grey | 0 |
| LOC730755 | 0.186913435 | 0.029970385 | grey | 0 |
| C19orf25 | -0.078404682 | 0.029992761 | grey | 0 |
| SMARCA4 | -0.097531953 | 0.030069113 | grey | 0 |
| WBSCR22 | -0.136589573 | 0.030076841 | grey | 0 |
| SRGAP2P1 | 0.167045911 | 0.030077244 | grey | 0 |
| DRD3 | 0.261415435 | 0.03009229 | grey | 0 |
| SNAP25 | 0.203560181 | 0.030261495 | grey | 0 |
| BIVM | -0.210754871 | 0.030288139 | grey | 0 |
| ENO1-AS1 | -0.134897754 | 0.030303081 | grey | 0 |
| G2E3 | -0.149638946 | 0.030306816 | grey | 0 |
| GDPD2 | 0.194837601 | 0.030308923 | grey | 0 |
| MST1R | -0.256521946 | 0.030334628 | grey | 0 |
| MINK1 | 0.113689253 | 0.030343381 | grey | 0 |
| AUH | 0.084824588 | 0.030377578 | grey | 0 |
| PFKL | -0.099386239 | 0.030379068 | grey | 0 |
| ARHGAP25 | 0.167801986 | 0.030429754 | grey | 0 |
| DOK6 | -0.176944732 | 0.030448267 | grey | 0 |
| SFTA1P | -0.142364946 | 0.030544347 | grey | 0 |
| PDGFRB | 0.306819063 | 0.030568184 | grey | 0 |
| ENPEP | 0.111670486 | 0.030570036 | grey | 0 |
| LOC401098 | 0.054395309 | 0.030577757 | grey | 0 |
| WDR87 | -0.066818702 | 0.030635239 | grey | 0 |
| LOC100505894 | 0.257630875 | 0.030667845 | grey | 0 |
| PPFIBP2 | -0.133434999 | 0.030675828 | grey | 0 |
| SPP1 | -0.30451029 | 0.030689951 | grey | 0 |
| HENMT1 | 0.122699852 | 0.030714822 | grey | 0 |
| PRICKLE1 | -0.238627834 | 0.030717247 | grey | 0 |
| FBXW10 | -0.200966735 | 0.030787309 | grey | 0 |
| LOC100506528 | 0.171582209 | 0.030971974 | grey | 0 |
| UGT2A3 | 0.050510902 | 0.030978855 | grey | 0 |
| RIPK1 | 0.105078195 | 0.031001416 | grey | 0 |
| WDR43 | -0.133749385 | 0.031084402 | grey | 0 |
| CNBD1 | -0.08979338 | 0.031086506 | grey | 0 |
| NDNF | 0.193110417 | 0.031124722 | grey | 0 |
| C1QTNF3 | -0.18025053 | 0.031151631 | grey | 0 |
| MDFI | 0.108734596 | 0.031163003 | grey | 0 |
| RTTN | -0.152218835 | 0.031216295 | grey | 0 |
| GOLGA2P2Y | -0.253437701 | 0.031236038 | grey | 0 |
| NOL12 | 0.071885655 | 0.031251578 | grey | 0 |
| KHNYN | -0.070705847 | 0.031259261 | grey | 0 |
| BCL2L1 | -0.190457922 | 0.03133206 | grey | 0 |
| ADRA1D | 0.15729599 | 0.031351403 | grey | 0 |
| BCL11A | -0.204013634 | 0.031420804 | grey | 0 |
| SNRNP27 | 0.093661532 | 0.03144416 | grey | 0 |
| DEFB129 | 0.182983406 | 0.031465729 | grey | 0 |
| NAA25 | -0.235265185 | 0.031503595 | grey | 0 |
| POMT2 | -0.125826893 | 0.031562169 | grey | 0 |
| RFX6 | -0.056405748 | 0.031599142 | grey | 0 |
| TMEM114 | 0.18243013 | 0.031610854 | grey | 0 |
| GOLIM4 | 0.180122327 | 0.031629609 | grey | 0 |
| HAUS5 | -0.26847021 | 0.031674462 | grey | 0 |
| MRC1 | -0.448741655 | 0.031723216 | grey | 0 |
| ANKRD30BP2 | 0.148768203 | 0.031757962 | grey | 0 |
| OR4S1 | -0.156908858 | 0.03178464 | grey | 0 |
| HTR2A | 0.163323143 | 0.031796385 | grey | 0 |
| CCDC142 | -0.101959471 | 0.031928285 | grey | 0 |
| PON3 | 0.257909774 | 0.031995171 | grey | 0 |
| CACNB3 | 0.097416521 | 0.032049001 | grey | 0 |
| KBTBD6 | -0.37848729 | 0.032086284 | grey | 0 |
| MT1M | -0.189837246 | 0.032328966 | grey | 0 |
| PTCD2 | -0.154261889 | 0.032345401 | grey | 0 |
| DPH5 | -0.094706161 | 0.032437448 | grey | 0 |
| BTBD2 | -0.103977351 | 0.032457178 | grey | 0 |
| LOC100505915 | -0.188164602 | 0.032584761 | grey | 0 |
| PRRT2 | -0.170612486 | 0.032647234 | grey | 0 |
| PHF21B | 0.177562882 | 0.032654828 | grey | 0 |
| ST6GAL1 | -0.216012873 | 0.032670015 | grey | 0 |
| PLEKHG1 | -0.414720581 | 0.032676335 | grey | 0 |
| AQP2 | 0.102749727 | 0.032755362 | grey | 0 |
| C11orf95 | -0.213608214 | 0.03278277 | grey | 0 |
| SLC2A9 | -0.179469054 | 0.032815104 | grey | 0 |
| TBCK | -0.137190271 | 0.03292465 | grey | 0 |
| ABCA3 | -0.125348168 | 0.032966652 | grey | 0 |
| SMPX | 0.209077412 | 0.032978466 | grey | 0 |
| NFKBIL1 | -0.190091217 | 0.033008614 | grey | 0 |
| PFN1P2 | 0.096785098 | 0.033042459 | grey | 0 |
| ZNF835 | -0.235081637 | 0.033042801 | grey | 0 |
| IDH1 | 0.119150271 | 0.033056907 | grey | 0 |
| OR5AK2 | -0.064019817 | 0.033072706 | grey | 0 |
| CEP135 | -0.149797128 | 0.033092453 | grey | 0 |
| CELF5 | -0.188223408 | 0.03309432 | grey | 0 |
| KIF26B | 0.133323163 | 0.033127781 | grey | 0 |
| C21orf104 | 0.106499076 | 0.033155741 | grey | 0 |
| GPC6 | 0.139096713 | 0.033168244 | grey | 0 |
| SCTR | 0.120267245 | 0.033273424 | grey | 0 |
| ULBP1 | 0.08819232 | 0.033410895 | grey | 0 |
| EIF2C3 | 0.094720293 | 0.033416828 | grey | 0 |
| C1orf51 | 0.154927513 | 0.033477275 | grey | 0 |
| DLX1 | 0.145162632 | 0.033495823 | grey | 0 |
| OMP | -0.287402829 | 0.033505401 | grey | 0 |
| C2orf81 | -0.257373432 | 0.0335166 | grey | 0 |
| FASN | -0.205658138 | 0.033525357 | grey | 0 |
| ZFP28 | -0.115880915 | 0.033536669 | grey | 0 |
| MGA | -0.134625314 | 0.033668526 | grey | 0 |
| POLR2E | -0.090494824 | 0.033750707 | grey | 0 |
| MBTD1 | -0.137917231 | 0.033764743 | grey | 0 |
| MEOX1 | -0.314381305 | 0.03378515 | grey | 0 |
| SPINT2 | -0.101187924 | 0.033820312 | grey | 0 |
| EI24 | -0.093516455 | 0.033901457 | grey | 0 |
| CYB5D2 | -0.070249515 | 0.033910819 | grey | 0 |
| SERTAD2 | -0.152219897 | 0.034086687 | grey | 0 |
| ZNF503-AS1 | 0.18859771 | 0.034240319 | grey | 0 |
| TRIM31 | -0.075232087 | 0.034262405 | grey | 0 |
| XRCC1 | -0.066487706 | 0.03429619 | grey | 0 |
| EFNA3 | 0.195202969 | 0.034448925 | grey | 0 |
| FAM166B | 0.203775365 | 0.034768948 | grey | 0 |
| KLC4 | -0.184457885 | 0.034863509 | grey | 0 |
| MX1 | 0.296961014 | 0.035052123 | grey | 0 |
| GPD1L | -0.149030144 | 0.035133839 | grey | 0 |
| HIF3A | 0.113638689 | 0.035135536 | grey | 0 |
| DTWD2 | -0.198533897 | 0.035179014 | grey | 0 |
| ZNF513 | -0.099097328 | 0.035229357 | grey | 0 |
| EXPH5 | -0.214824736 | 0.035259428 | grey | 0 |
| PHLDA1 | -0.114136422 | 0.035263362 | grey | 0 |
| TRPT1 | -0.05980658 | 0.035263821 | grey | 0 |
| SAV1 | -0.151454669 | 0.035289774 | grey | 0 |
| FRMD5 | 0.17393655 | 0.035306049 | grey | 0 |
| PRPF19 | -0.145972029 | 0.035307634 | grey | 0 |
| ERAP2 | -0.326941487 | 0.035308888 | grey | 0 |
| EPHA8 | 0.100819674 | 0.035311856 | grey | 0 |
| ABCD2 | -0.389548611 | 0.035373979 | grey | 0 |
| GIMAP2 | 0.104078579 | 0.035429434 | grey | 0 |
| FAM76A | -0.110571702 | 0.035537833 | grey | 0 |
| ABCF3 | -0.069188586 | 0.035545306 | grey | 0 |
| C4orf26 | -0.07528885 | 0.03556921 | grey | 0 |
| RPP30 | -0.106019377 | 0.035579343 | grey | 0 |
| TFAP4 | 0.227869632 | 0.03558101 | grey | 0 |
| BCAR1 | 0.091182025 | 0.035671967 | grey | 0 |
| PP12719 | 0.251846906 | 0.035740862 | grey | 0 |
| DNM1P46 | 0.171307277 | 0.035760604 | grey | 0 |
| C11orf65 | -0.215179626 | 0.03594015 | grey | 0 |
| SPEF2 | -0.160381393 | 0.035946801 | grey | 0 |
| MASP1 | 0.127261266 | 0.036018002 | grey | 0 |
| TMEM191A | -0.116054735 | 0.036132978 | grey | 0 |
| ANO6 | -0.186553915 | 0.036171583 | grey | 0 |
| NCAPD2 | -0.100111256 | 0.036196908 | grey | 0 |
| E4F1 | -0.128009476 | 0.036210592 | grey | 0 |
| FAM111A | 0.133613362 | 0.036249009 | grey | 0 |
| RASGRP3 | -0.366792831 | 0.036250927 | grey | 0 |
| RING1 | -0.077258962 | 0.036266414 | grey | 0 |
| C3orf24 | -0.072692092 | 0.03641902 | grey | 0 |
| POU6F1 | -0.187223429 | 0.036463892 | grey | 0 |
| LMNA | -0.143851505 | 0.036501135 | grey | 0 |
| PNMA3 | -0.202634981 | 0.036512828 | grey | 0 |
| ACAD8 | 0.100870366 | 0.036533349 | grey | 0 |
| ZNF799 | -0.247271285 | 0.036538942 | grey | 0 |
| DOM3Z | -0.105270392 | 0.036670977 | grey | 0 |
| RALGPS2 | -0.252066276 | 0.036672711 | grey | 0 |
| VPREB3 | -0.307569013 | 0.03671026 | grey | 0 |
| C12orf76 | -0.120599285 | 0.036711387 | grey | 0 |
| SSBP2 | -0.185063173 | 0.036774205 | grey | 0 |
| NME4 | -0.122466285 | 0.036833604 | grey | 0 |
| DTX1 | -0.179268458 | 0.03686802 | grey | 0 |
| ZNF576 | -0.11122267 | 0.036869571 | grey | 0 |
| ZFP90 | -0.145769179 | 0.0369107 | grey | 0 |
| PITX2 | 0.172862409 | 0.036917107 | grey | 0 |
| HOXA13 | 0.151794773 | 0.037030581 | grey | 0 |
| NSL1 | 0.128804834 | 0.03706673 | grey | 0 |
| PLD6 | -0.185752769 | 0.037069375 | grey | 0 |
| OIT3 | -0.080930859 | 0.037148111 | grey | 0 |
| LOC100505663 | -0.071347334 | 0.037164502 | grey | 0 |
| NPR2 | -0.292235026 | 0.037271733 | grey | 0 |
| OGDH | -0.099181174 | 0.037279848 | grey | 0 |
| ILK | -0.093055282 | 0.037290066 | grey | 0 |
| USP39 | 0.065705572 | 0.037291132 | grey | 0 |
| PIEZO2 | 0.105927334 | 0.037329606 | grey | 0 |
| CCDC80 | -0.142884556 | 0.037386985 | grey | 0 |
| PKIG | -0.162663065 | 0.037428755 | grey | 0 |
| ARTN | -0.189058945 | 0.037441178 | grey | 0 |
| KIF12 | 0.093506706 | 0.037469156 | grey | 0 |
| LOC100652917 | -0.334981075 | 0.037540381 | grey | 0 |
| TRAPPC9 | -0.089872945 | 0.037551197 | grey | 0 |
| MVK | 0.141892161 | 0.037560929 | grey | 0 |
| CHI3L2 | -0.235260702 | 0.03756386 | grey | 0 |
| DMD | -0.072144244 | 0.037608711 | grey | 0 |
| SARNP | 0.0890836 | 0.037656429 | grey | 0 |
| DIRC1 | 0.286396149 | 0.037885091 | grey | 0 |
| GGT1 | -0.112072359 | 0.037899002 | grey | 0 |
| LRAT | 0.110636058 | 0.038021082 | grey | 0 |
| XAB2 | -0.202133692 | 0.038132675 | grey | 0 |
| LGR4 | -0.157550142 | 0.038169904 | grey | 0 |
| C12orf57 | -0.126404002 | 0.038273261 | grey | 0 |
| ARMC3 | -0.172704927 | 0.038276391 | grey | 0 |
| BCR | -0.078424342 | 0.038376854 | grey | 0 |
| ZSCAN18 | -0.171762962 | 0.03840068 | grey | 0 |
| PRDM13 | -0.24713638 | 0.038428046 | grey | 0 |
| RECQL5 | -0.12532975 | 0.038487588 | grey | 0 |
| SNTB1 | 0.167042064 | 0.038593981 | grey | 0 |
| LOC653075 | -0.111808821 | 0.038619741 | grey | 0 |
| GPR22 | -0.048682866 | 0.038621141 | grey | 0 |
| SRSF3 | 0.09050822 | 0.038649577 | grey | 0 |
| LOC541471 | 0.102740166 | 0.038744151 | grey | 0 |
| PDE8B | -0.190940777 | 0.038760137 | grey | 0 |
| PRSS1 | 0.119734464 | 0.038847519 | grey | 0 |
| DDX4 | -0.036753363 | 0.038871325 | grey | 0 |
| IL6 | -0.167468243 | 0.03887424 | grey | 0 |
| SPOCK1 | -0.27132327 | 0.038905484 | grey | 0 |
| SMARCA2 | 0.086611699 | 0.038938476 | grey | 0 |
| C20orf27 | -0.167674097 | 0.0389863 | grey | 0 |
| AADAT | -0.19343684 | 0.039012567 | grey | 0 |
| C11orf51 | -0.112245173 | 0.039064248 | grey | 0 |
| NEK8 | 0.179629093 | 0.039075717 | grey | 0 |
| POM121L1P | 0.26141958 | 0.03913254 | grey | 0 |
| FAM89B | 0.066365441 | 0.039173917 | grey | 0 |
| STAT1 | 0.190171373 | 0.039183359 | grey | 0 |
| RBFOX1 | 0.120979142 | 0.039232999 | grey | 0 |
| RGAG4 | -0.300656673 | 0.039337837 | grey | 0 |
| PSME1 | 0.104789101 | 0.039407822 | grey | 0 |
| BLZF1 | 0.178652684 | 0.039479058 | grey | 0 |
| ZNF536 | -0.146615298 | 0.039616971 | grey | 0 |
| CNGA4 | -0.186536221 | 0.03963075 | grey | 0 |
| TATDN1 | -0.105564255 | 0.039644884 | grey | 0 |
| RASGRF2 | -0.336438402 | 0.039654625 | grey | 0 |
| PSMA6 | 0.079656987 | 0.039658604 | grey | 0 |
| AGSK1 | -0.102352641 | 0.039659116 | grey | 0 |
| FADS3 | -0.103326561 | 0.039667632 | grey | 0 |
| PSTK | -0.177226984 | 0.039682698 | grey | 0 |
| SCAPER | -0.167237668 | 0.039721598 | grey | 0 |
| IFIT2 | 0.284255497 | 0.039763095 | grey | 0 |
| NLRP10 | 0.088946891 | 0.039905601 | grey | 0 |
| HIC1 | -0.177861525 | 0.039952963 | grey | 0 |
| TTL | -0.095288539 | 0.039967924 | grey | 0 |
| FAM195A | -0.1458197 | 0.040008282 | grey | 0 |
| PLXDC1 | -0.357387785 | 0.040165632 | grey | 0 |
| SETD1A | -0.260008343 | 0.040191219 | grey | 0 |
| C2orf44 | -0.169475834 | 0.04022805 | grey | 0 |
| DNAJC14 | 0.099796308 | 0.040255763 | grey | 0 |
| GALK2 | 0.059812764 | 0.040300407 | grey | 0 |
| LOC100128843 | 0.352790841 | 0.040386728 | grey | 0 |
| LGI1 | 0.181160056 | 0.040473637 | grey | 0 |
| CTNND1 | -0.136389995 | 0.040474651 | grey | 0 |
| PEBP4 | -0.128161469 | 0.040487884 | grey | 0 |
| CDC14B | -0.131862803 | 0.040495382 | grey | 0 |
| ACADS | -0.247699785 | 0.040525101 | grey | 0 |
| NR1D2 | -0.168559844 | 0.040572491 | grey | 0 |
| LOC200726 | -0.178497522 | 0.04059721 | grey | 0 |
| SREK1 | 0.06741647 | 0.040687676 | grey | 0 |
| BATF2 | 0.272839964 | 0.04069089 | grey | 0 |
| CES4A | -0.205684908 | 0.040693288 | grey | 0 |
| ACD | -0.087423509 | 0.040717111 | grey | 0 |
| IL17RD | 0.125133549 | 0.040722511 | grey | 0 |
| MYEF2 | -0.320657021 | 0.040765617 | grey | 0 |
| 4-Mar | 0.136821343 | 0.040791287 | grey | 0 |
| GALNT13 | 0.067729824 | 0.040794349 | grey | 0 |
| EPHA1 | -0.262396602 | 0.040795633 | grey | 0 |
| ZNF264 | -0.138129976 | 0.040840729 | grey | 0 |
| OR52K3P | 0.30588176 | 0.040951467 | grey | 0 |
| AGBL1 | 0.154067035 | 0.041067888 | grey | 0 |
| HDAC11 | -0.245022639 | 0.041070815 | grey | 0 |
| NUDT3 | 0.090188839 | 0.04107333 | grey | 0 |
| SLC6A15 | 0.133880775 | 0.041120085 | grey | 0 |
| HSP90AA1 | 0.101751084 | 0.041164642 | grey | 0 |
| TMEM145 | 0.112915502 | 0.041344628 | grey | 0 |
| ANXA7 | 0.084513887 | 0.041355085 | grey | 0 |
| C20orf165 | 0.265681247 | 0.041476051 | grey | 0 |
| RPS2 | -0.061945945 | 0.041485852 | grey | 0 |
| CNTROB | -0.076807863 | 0.041525168 | grey | 0 |
| BMP15 | -0.045306418 | 0.041566458 | grey | 0 |
| NKAIN1 | 0.252802752 | 0.041697173 | grey | 0 |
| ZKSCAN5 | -0.13026477 | 0.041712017 | grey | 0 |
| PGK2 | 0.076081645 | 0.041739289 | grey | 0 |
| PDE1C | 0.181560559 | 0.041782128 | grey | 0 |
| RASL11A | -0.340212247 | 0.041790253 | grey | 0 |
| LOC100506930 | -0.124975382 | 0.041869595 | grey | 0 |
| KIAA0195 | -0.115254761 | 0.041901384 | grey | 0 |
| ECT2 | 0.138203208 | 0.041910929 | grey | 0 |
| ZNF287 | -0.287500116 | 0.042111476 | grey | 0 |
| PART1 | 0.101832205 | 0.042175894 | grey | 0 |
| NUP155 | -0.099637439 | 0.042197205 | grey | 0 |
| NEK2 | 0.207567963 | 0.042208949 | grey | 0 |
| SYCE2 | -0.270620129 | 0.042245069 | grey | 0 |
| ANKRD58 | 0.112385737 | 0.042304209 | grey | 0 |
| SLC39A3 | -0.111457267 | 0.04232208 | grey | 0 |
| TSHZ2 | -0.306091773 | 0.042337732 | grey | 0 |
| GAD1 | 0.115501882 | 0.042343532 | grey | 0 |
| CRLF2 | -0.188237272 | 0.042363286 | grey | 0 |
| PCDP1 | -0.078253345 | 0.042411711 | grey | 0 |
| APOL1 | 0.173274308 | 0.042429597 | grey | 0 |
| GABBR2 | 0.161366896 | 0.042443639 | grey | 0 |
| FMO4 | -0.268698184 | 0.042466503 | grey | 0 |
| VHLL | -0.27420505 | 0.042483304 | grey | 0 |
| HDHD3 | -0.264024785 | 0.042549391 | grey | 0 |
| ACP2 | -0.172471255 | 0.042578871 | grey | 0 |
| TNFAIP3 | 0.200398617 | 0.042654701 | grey | 0 |
| KRTAP13-1 | -0.187966933 | 0.042814543 | grey | 0 |
| GRIA3 | 0.079351849 | 0.042886 | grey | 0 |
| C3orf80 | 0.211573392 | 0.042914728 | grey | 0 |
| KPNA1 | 0.215546671 | 0.042918247 | grey | 0 |
| VRK2 | 0.118849344 | 0.042948832 | grey | 0 |
| REXO1L1 | 0.281958471 | 0.042976205 | grey | 0 |
| IL22RA2 | -0.055388062 | 0.043044402 | grey | 0 |
| MTBP | 0.171898596 | 0.043057075 | grey | 0 |
| BAD | -0.054242553 | 0.043176832 | grey | 0 |
| CXADRP2 | 0.208194847 | 0.043183602 | grey | 0 |
| DACT1 | -0.292359927 | 0.043186508 | grey | 0 |
| DEFA4 | 0.44360751 | 0.043214778 | grey | 0 |
| KCTD12 | 0.144615208 | 0.043331376 | grey | 0 |
| ELF4 | 0.13059265 | 0.043375619 | grey | 0 |
| G6PC | 0.102639314 | 0.043404396 | grey | 0 |
| LOC96610 | 0.178446633 | 0.043407646 | grey | 0 |
| SLC25A35 | -0.173486527 | 0.043475854 | grey | 0 |
| HMX1 | -0.252890238 | 0.043479814 | grey | 0 |
| TERF2IP | 0.061238794 | 0.043482729 | grey | 0 |
| PCDH9 | -0.234306458 | 0.043485007 | grey | 0 |
| ANKRD11 | 0.099061632 | 0.04351378 | grey | 0 |
| GLTPD2 | 0.227081557 | 0.043547691 | grey | 0 |
| MRRF | -0.147507098 | 0.043568655 | grey | 0 |
| KISS1 | -0.286384657 | 0.043672384 | grey | 0 |
| PNMA6A | -0.24406122 | 0.043756104 | grey | 0 |
| SVIP | -0.144328725 | 0.0438256 | grey | 0 |
| PTGES | 0.216425012 | 0.043863454 | grey | 0 |
| DENND4C | -0.181383695 | 0.043885845 | grey | 0 |
| LOC100506687 | 0.166301938 | 0.043890238 | grey | 0 |
| LOC100289255 | 0.180063596 | 0.043946942 | grey | 0 |
| C4orf48 | -0.104461117 | 0.043970194 | grey | 0 |
| PLD4 | -0.296966376 | 0.044039542 | grey | 0 |
| PSMF1 | -0.053429948 | 0.044161981 | grey | 0 |
| OR51B4 | 0.087642797 | 0.044185849 | grey | 0 |
| RNF208 | -0.23824237 | 0.044188483 | grey | 0 |
| DDX58 | 0.222852431 | 0.044233321 | grey | 0 |
| PHKG2 | -0.110187321 | 0.044268537 | grey | 0 |
| ARHGAP21 | -0.176836851 | 0.044318622 | grey | 0 |
| PSG4 | -0.182230084 | 0.044336474 | grey | 0 |
| TSPAN4 | -0.072092767 | 0.044369305 | grey | 0 |
| APBA2 | -0.225666354 | 0.044446677 | grey | 0 |
| SEPSECS | 0.183452599 | 0.044482301 | grey | 0 |
| ADH1C | 0.205528328 | 0.044502108 | grey | 0 |
| HDHD1 | -0.114933163 | 0.044609887 | grey | 0 |
| PCSK4 | -0.137303696 | 0.044633581 | grey | 0 |
| PITPNM1 | -0.119962828 | 0.044710259 | grey | 0 |
| PSMD12 | 0.059454745 | 0.044758767 | grey | 0 |
| FAM19A1 | -0.416363354 | 0.044906632 | grey | 0 |
| LOC100507431 | -0.063646993 | 0.044954251 | grey | 0 |
| SNX22 | -0.176497144 | 0.045025526 | grey | 0 |
| KREMEN2 | -0.197894911 | 0.045082244 | grey | 0 |
| IGFBPL1 | 0.138179891 | 0.045116752 | grey | 0 |
| INPP4A | 0.0981227 | 0.045307931 | grey | 0 |
| DDX19B | -0.067948089 | 0.045444433 | grey | 0 |
| LOC100129973 | -0.220499336 | 0.045457376 | grey | 0 |
| DAPP1 | 0.130821167 | 0.045474659 | grey | 0 |
| LOC441461 | -0.257389934 | 0.045482118 | grey | 0 |
| HTR3C | -0.104862809 | 0.045486493 | grey | 0 |
| LOC100144602 | 0.159384042 | 0.0454879 | grey | 0 |
| FLJ13224 | -0.194221165 | 0.04549333 | grey | 0 |
| LOC100127891 | 0.129157163 | 0.045516217 | grey | 0 |
| ANXA8L2 | 0.181295328 | 0.045516909 | grey | 0 |
| POLD4 | -0.057223698 | 0.045519534 | grey | 0 |
| H1FOO | -0.106553496 | 0.045578929 | grey | 0 |
| TSTA3 | -0.076560193 | 0.045692062 | grey | 0 |
| LOC100652972 | -0.259108717 | 0.045712784 | grey | 0 |
| PCDHB7 | 0.258753582 | 0.045718964 | grey | 0 |
| RNF152 | 0.097601731 | 0.045751517 | grey | 0 |
| C1orf172 | -0.293609667 | 0.045763408 | grey | 0 |
| MAB21L2 | 0.422476865 | 0.045767682 | grey | 0 |
| C12orf66 | -0.164809575 | 0.045805104 | grey | 0 |
| GRHL1 | 0.260636383 | 0.045810006 | grey | 0 |
| NAT6 | -0.116086994 | 0.045878593 | grey | 0 |
| LOC255177 | -0.049044478 | 0.046020727 | grey | 0 |
| TMEM132C | -0.14185244 | 0.046087596 | grey | 0 |
| SLC6A12 | -0.418673797 | 0.046108761 | grey | 0 |
| ZNF304 | -0.158761656 | 0.046139467 | grey | 0 |
| KIAA0319 | 0.286765682 | 0.04621807 | grey | 0 |
| KRT76 | 0.231826375 | 0.046222613 | grey | 0 |
| SLC35F1 | 0.18228499 | 0.046313736 | grey | 0 |
| PCDH1 | 0.150030761 | 0.046353181 | grey | 0 |
| THRSP | -0.098988995 | 0.046374908 | grey | 0 |
| SWAP70 | -0.159192474 | 0.046468817 | grey | 0 |
| POM121L10P | 0.076792642 | 0.0465233 | grey | 0 |
| FLAD1 | -0.062923893 | 0.046532243 | grey | 0 |
| FAM92B | 0.120402578 | 0.046620549 | grey | 0 |
| EXOSC5 | -0.111056802 | 0.046727354 | grey | 0 |
| HEATR6 | -0.114096493 | 0.046791787 | grey | 0 |
| ABCC11 | 0.197367246 | 0.046908218 | grey | 0 |
| SBK2 | 0.203540062 | 0.046915381 | grey | 0 |
| C6orf124 | 0.395979053 | 0.046962851 | grey | 0 |
| CDK2AP1 | -0.14743236 | 0.047037372 | grey | 0 |
| C21orf56 | -0.238612941 | 0.047105691 | grey | 0 |
| IL12RB1 | 0.207476874 | 0.047124506 | grey | 0 |
| PSMB4 | 0.065102909 | 0.047147258 | grey | 0 |
| EDEM1 | 0.115342209 | 0.047165859 | grey | 0 |
| KRT35 | 0.13519516 | 0.047295137 | grey | 0 |
| P2RX5 | -0.209763453 | 0.047344782 | grey | 0 |
| C2orf73 | 0.083789293 | 0.047413067 | grey | 0 |
| NIT2 | -0.153504017 | 0.047602973 | grey | 0 |
| SPCS3 | 0.115511649 | 0.047691593 | grey | 0 |
| GGPS1 | 0.07152842 | 0.047719438 | grey | 0 |
| HMP19 | 0.214673262 | 0.047823946 | grey | 0 |
| GPR112 | 0.094493407 | 0.047898112 | grey | 0 |
| NFX1 | -0.078317926 | 0.048020295 | grey | 0 |
| RAPGEF1 | -0.151523855 | 0.048026386 | grey | 0 |
| SLC25A31 | 0.038803455 | 0.048061554 | grey | 0 |
| TDRG1 | 0.052298348 | 0.048111959 | grey | 0 |
| LRRTM2 | -0.141296119 | 0.048140902 | grey | 0 |
| ANAPC7 | -0.155256792 | 0.048162554 | grey | 0 |
| C10orf25 | -0.225322411 | 0.048195819 | grey | 0 |
| C12orf50 | 0.100599751 | 0.048210643 | grey | 0 |
| TRAPPC10 | 0.078139274 | 0.048218637 | grey | 0 |
| LOC100505870 | 0.095677548 | 0.048361761 | grey | 0 |
| POLN | 0.081435884 | 0.048501409 | grey | 0 |
| C6orf145 | 0.124544224 | 0.048504621 | grey | 0 |
| FXYD4 | 0.239861337 | 0.048587665 | grey | 0 |
| MYCBPAP | -0.321322291 | 0.048614878 | grey | 0 |
| TMEM223 | -0.113947762 | 0.048647107 | grey | 0 |
| KCTD4 | 0.040465682 | 0.048693292 | grey | 0 |
| RAB30 | -0.2565025 | 0.048755754 | grey | 0 |
| BTBD9 | -0.137965489 | 0.048818295 | grey | 0 |
| CALCA | 0.078917489 | 0.048826884 | grey | 0 |
| LOC100130950 | -0.260273806 | 0.048845982 | grey | 0 |
| ATP8A2 | -0.246316878 | 0.048884066 | grey | 0 |
| CRYAB | -0.218967289 | 0.048899949 | grey | 0 |
| C17orf98 | -0.103918438 | 0.048974661 | grey | 0 |
| CERS6 | -0.155925687 | 0.048988879 | grey | 0 |
| KBTBD8 | -0.233918912 | 0.049009391 | grey | 0 |
| MLLT3 | -0.217747229 | 0.049056507 | grey | 0 |
| STX1B | 0.189293395 | 0.049066344 | grey | 0 |
| PGA3 | -0.387443586 | 0.049101061 | grey | 0 |
| GALNT9 | 0.205565364 | 0.049125475 | grey | 0 |
| CNTN6 | -0.176895552 | 0.049146364 | grey | 0 |
| LOC100131662 | -0.261758164 | 0.049197348 | grey | 0 |
| KIAA1274 | -0.26399126 | 0.049243269 | grey | 0 |
| IRF4 | -0.209985588 | 0.049346699 | grey | 0 |
| PKD1P1 | -0.088102 | 0.049347749 | grey | 0 |
| LOC100131176 | -0.470021241 | 0.049412061 | grey | 0 |
| IL17B | 0.179172665 | 0.049449545 | grey | 0 |
| TREML3 | -0.341159733 | 0.049470573 | grey | 0 |
| RFTN2 | 0.065790121 | 0.049554532 | grey | 0 |
| TMX1 | 0.108917209 | 0.049627936 | grey | 0 |
| PLAGL1 | 0.156873493 | 0.049693062 | grey | 0 |
| PLXNA1 | -0.104192195 | 0.04975537 | grey | 0 |
| THSD4 | 0.102115021 | 0.049802637 | grey | 0 |
| CADPS2 | 0.176553282 | 0.049823989 | grey | 0 |
| HIRIP3 | -0.131045206 | 0.049862988 | grey | 0 |
| AGL | -0.10837452 | 0.049866072 | grey | 0 |
| POU5F2 | -0.175436547 | 0.049950563 | grey | 0 |
| GJA10 | 0.049093275 | 0.014778613 | grey | 1 |
| DAOA-AS1 | 0.037495041 | 0.047922422 | grey | 1 |
| LOC100128185 | 0.037951734 | 0.046934532 | grey | 2 |
| DDX3Y | 1.283437623 | 0.000957015 | grey | 6 |
| XIST | -2.492702362 | 0.000973749 | grey | 6 |
| KDM5D | 1.42621414 | 0.001575567 | grey | 6 |
| EIF1AY | 1.246290778 | 0.001827949 | grey | 6 |
| RPS4Y2 | 1.706044091 | 0.001828556 | grey | 6 |
| TXLNG2P | 1.152545186 | 0.001909191 | grey | 6 |
| RPS4Y1 | 1.856142011 | 0.002640669 | grey | 6 |
| SNURF | -0.339286818 | 0.000128593 | turquoise | 0 |
| PRKX | -0.321630863 | 0.000234963 | turquoise | 0 |
| WIPI1 | 0.296878918 | 0.00030939 | turquoise | 0 |
| ESF1 | -0.29225802 | 0.000427843 | turquoise | 0 |
| SRP14 | 0.162916636 | 0.000546734 | turquoise | 0 |
| ABR | 0.160343503 | 0.000582998 | turquoise | 0 |
| RRAGC | 0.138434122 | 0.000845654 | turquoise | 0 |
| RG9MTD3 | -0.183718409 | 0.000910491 | turquoise | 0 |
| RDH11 | -0.172824234 | 0.00095069 | turquoise | 0 |
| MTG1 | -0.232599255 | 0.001048585 | turquoise | 0 |
| ZNF789 | -0.263437166 | 0.001281762 | turquoise | 0 |
| ZBED3 | -0.219248931 | 0.001297314 | turquoise | 0 |
| SNHG10 | -0.237381861 | 0.001365887 | turquoise | 0 |
| ILVBL | -0.163660926 | 0.001687155 | turquoise | 0 |
| COTL1 | 0.180190472 | 0.001739133 | turquoise | 0 |
| TGFBR2 | 0.174875337 | 0.002028796 | turquoise | 0 |
| EFHD2 | 0.145485847 | 0.002161661 | turquoise | 0 |
| LCP1 | 0.264449765 | 0.002216205 | turquoise | 0 |
| PAN3 | 0.190659584 | 0.002321224 | turquoise | 0 |
| CYP2U1 | -0.189944647 | 0.002346731 | turquoise | 0 |
| ZNF444 | -0.102088712 | 0.002355373 | turquoise | 0 |
| ERBB2IP | 0.147075218 | 0.00251745 | turquoise | 0 |
| GMFG | 0.276196457 | 0.002577364 | turquoise | 0 |
| CRADD | 0.167420871 | 0.002586187 | turquoise | 0 |
| GIT2 | 0.141399667 | 0.002676155 | turquoise | 0 |
| CD40 | -0.313413942 | 0.002697114 | turquoise | 0 |
| USP15 | 0.223480829 | 0.002719785 | turquoise | 0 |
| RBP7 | 0.193540682 | 0.002807569 | turquoise | 0 |
| VCPIP1 | 0.153944309 | 0.002821304 | turquoise | 0 |
| ITPR2 | 0.234885537 | 0.002886921 | turquoise | 0 |
| AKR7A3 | -0.12493414 | 0.002898872 | turquoise | 0 |
| IFITM1 | 0.240370865 | 0.002899668 | turquoise | 0 |
| TOR1AIP1 | 0.176319204 | 0.002938734 | turquoise | 0 |
| NMI | 0.2355538 | 0.002983922 | turquoise | 0 |
| LRRC56 | -0.232826702 | 0.003141219 | turquoise | 0 |
| NIPAL3 | -0.210568063 | 0.00327688 | turquoise | 0 |
| FAM27A | 0.368943726 | 0.0033102 | turquoise | 0 |
| SHISA5 | 0.192829541 | 0.003396161 | turquoise | 0 |
| PSMC4 | -0.100376138 | 0.003439297 | turquoise | 0 |
| DEDD | 0.136059288 | 0.0035065 | turquoise | 0 |
| CAPZA1 | 0.246098959 | 0.003570601 | turquoise | 0 |
| C6orf62 | 0.180335138 | 0.003603952 | turquoise | 0 |
| BRD9 | -0.140894873 | 0.003635425 | turquoise | 0 |
| MMS19 | -0.113286709 | 0.00367414 | turquoise | 0 |
| CEBPZ | -0.123301735 | 0.00376144 | turquoise | 0 |
| JDP2 | 0.195868255 | 0.003776553 | turquoise | 0 |
| RAB8B | 0.160689294 | 0.003824053 | turquoise | 0 |
| LOC100287628 | -0.239760297 | 0.003850237 | turquoise | 0 |
| KRCC1 | 0.129097943 | 0.004152908 | turquoise | 0 |
| WDR89 | -0.230399683 | 0.004154131 | turquoise | 0 |
| NBAS | -0.119097003 | 0.004357686 | turquoise | 0 |
| LOC155060 | -0.227255435 | 0.004466266 | turquoise | 0 |
| PLBD1 | 0.263918207 | 0.00448589 | turquoise | 0 |
| CIR1 | 0.171925948 | 0.004566592 | turquoise | 0 |
| SDR39U1 | -0.182153388 | 0.004601367 | turquoise | 0 |
| MTA3 | -0.13664144 | 0.004683188 | turquoise | 0 |
| LOC157562 | -0.21305085 | 0.00469112 | turquoise | 0 |
| DYNLT1 | 0.216743616 | 0.004786417 | turquoise | 0 |
| 9-Mar | -0.151361309 | 0.004801179 | turquoise | 0 |
| NFKBIA | 0.326256511 | 0.004853104 | turquoise | 0 |
| RMI1 | 0.187806618 | 0.00485345 | turquoise | 0 |
| PPP1R18 | 0.137775408 | 0.004989912 | turquoise | 0 |
| SAMM50 | -0.115522724 | 0.00505983 | turquoise | 0 |
| VTI1A | 0.095494431 | 0.005080407 | turquoise | 0 |
| RP9 | -0.125284386 | 0.005113341 | turquoise | 0 |
| C6orf47 | 0.112256555 | 0.00516382 | turquoise | 0 |
| KAT2A | -0.17408112 | 0.005174292 | turquoise | 0 |
| ATP1A3 | -0.147382714 | 0.005224307 | turquoise | 0 |
| KCNE1L | 0.21919766 | 0.005262575 | turquoise | 0 |
| ADAT2 | -0.200231632 | 0.005308585 | turquoise | 0 |
| DSCR3 | 0.110255604 | 0.005362763 | turquoise | 0 |
| OSTF1 | 0.170778843 | 0.005454258 | turquoise | 0 |
| FAM58A | -0.147608126 | 0.005620464 | turquoise | 0 |
| YWHAB | 0.125629903 | 0.00564334 | turquoise | 0 |
| DMAP1 | -0.121133784 | 0.005661552 | turquoise | 0 |
| ALDH16A1 | -0.163115334 | 0.005731823 | turquoise | 0 |
| GEMIN4 | -0.131550311 | 0.005841718 | turquoise | 0 |
| ABHD14A | -0.166456082 | 0.005885989 | turquoise | 0 |
| SQRDL | 0.203426047 | 0.005898565 | turquoise | 0 |
| RAD23A | -0.132308052 | 0.005916915 | turquoise | 0 |
| TRAPPC6A | -0.12829737 | 0.005976704 | turquoise | 0 |
| CD1C | -0.296193248 | 0.006018943 | turquoise | 0 |
| DDB1 | -0.118016082 | 0.006049817 | turquoise | 0 |
| DNAJA3 | -0.15390493 | 0.006059875 | turquoise | 0 |
| CYP4F2 | 0.278228425 | 0.006098842 | turquoise | 0 |
| GADD45B | 0.186016979 | 0.006147376 | turquoise | 0 |
| PIM1 | 0.147105161 | 0.006164794 | turquoise | 0 |
| CALM2 | 0.239241772 | 0.006213617 | turquoise | 0 |
| GRWD1 | -0.09391006 | 0.00625196 | turquoise | 0 |
| OVCA2 | -0.094995602 | 0.006319651 | turquoise | 0 |
| RIC3 | -0.402827701 | 0.006327434 | turquoise | 0 |
| GSR | 0.177024086 | 0.006405402 | turquoise | 0 |
| WAC | 0.113906366 | 0.006413912 | turquoise | 0 |
| ZNF500 | -0.280545403 | 0.006441002 | turquoise | 0 |
| KPNB1 | 0.247623714 | 0.006538364 | turquoise | 0 |
| PPIE | -0.181977183 | 0.006694871 | turquoise | 0 |
| ZCCHC6 | 0.203227494 | 0.006697799 | turquoise | 0 |
| HNRNPK | 0.131479527 | 0.006768933 | turquoise | 0 |
| PPP3R1 | 0.244224983 | 0.006797206 | turquoise | 0 |
| MCM7 | -0.122094666 | 0.006799526 | turquoise | 0 |
| EXOC1 | 0.096176182 | 0.006843859 | turquoise | 0 |
| PRKCB | 0.106670592 | 0.00692568 | turquoise | 0 |
| ZNF232 | -0.154845245 | 0.006996687 | turquoise | 0 |
| EVI2A | 0.180338803 | 0.007060357 | turquoise | 0 |
| STMN3 | -0.167500561 | 0.007068189 | turquoise | 0 |
| NENF | -0.155711048 | 0.00709897 | turquoise | 0 |
| PPT1 | 0.276279784 | 0.007158552 | turquoise | 0 |
| RSF1 | 0.095177424 | 0.007175121 | turquoise | 0 |
| PYGO2 | -0.073462814 | 0.007189916 | turquoise | 0 |
| ADAR | 0.174928722 | 0.007242441 | turquoise | 0 |
| PRR13 | 0.176476835 | 0.007282757 | turquoise | 0 |
| PPP2R3C | 0.173606951 | 0.007324123 | turquoise | 0 |
| GPR27 | 0.431224468 | 0.007426498 | turquoise | 0 |
| KDM5A | 0.110639367 | 0.007477935 | turquoise | 0 |
| OGFOD1 | -0.146155297 | 0.007530782 | turquoise | 0 |
| RAP2C | 0.181873981 | 0.007980769 | turquoise | 0 |
| ATP6V0E2 | -0.345296536 | 0.007993619 | turquoise | 0 |
| DGCR6L | -0.095103483 | 0.008017091 | turquoise | 0 |
| LOC338799 | -0.183934199 | 0.008057509 | turquoise | 0 |
| ELP2 | -0.215923439 | 0.008060389 | turquoise | 0 |
| TFRC | -0.193316612 | 0.008062811 | turquoise | 0 |
| CHUK | 0.248159135 | 0.008077396 | turquoise | 0 |
| CELF2 | 0.145831798 | 0.008253072 | turquoise | 0 |
| GABBR1 | -0.273684461 | 0.008367958 | turquoise | 0 |
| MLL5 | 0.092036082 | 0.008537562 | turquoise | 0 |
| HARS | -0.099125985 | 0.008602858 | turquoise | 0 |
| DOCK11 | 0.115181357 | 0.008665091 | turquoise | 0 |
| HINT3 | 0.174915277 | 0.008668526 | turquoise | 0 |
| FLI1 | 0.127644325 | 0.008685448 | turquoise | 0 |
| LOC100131170 | 0.288178333 | 0.008708392 | turquoise | 0 |
| AP2A2 | -0.089875189 | 0.008727431 | turquoise | 0 |
| UQCRB | -0.171409649 | 0.008915315 | turquoise | 0 |
| TOR1A | 0.170141262 | 0.008972797 | turquoise | 0 |
| RBM12 | -0.098353051 | 0.009108509 | turquoise | 0 |
| C19orf53 | -0.112659961 | 0.009208874 | turquoise | 0 |
| GTF3C5 | -0.080213628 | 0.009213782 | turquoise | 0 |
| HEBP2 | 0.233592182 | 0.009242447 | turquoise | 0 |
| ACAD9 | -0.15148345 | 0.009261728 | turquoise | 0 |
| CXCR4 | 0.220266797 | 0.009357834 | turquoise | 0 |
| RBM19 | -0.110614994 | 0.009373934 | turquoise | 0 |
| SEC22B | 0.191332449 | 0.00946312 | turquoise | 0 |
| WDR92 | -0.111081629 | 0.009485249 | turquoise | 0 |
| SAT1 | 0.268372596 | 0.009558704 | turquoise | 0 |
| RNF122 | 0.170872394 | 0.009609838 | turquoise | 0 |
| TELO2 | -0.115377687 | 0.009641698 | turquoise | 0 |
| RRP1 | -0.107534589 | 0.00975961 | turquoise | 0 |
| DDX60L | 0.270025217 | 0.009760092 | turquoise | 0 |
| SDHB | 0.096230326 | 0.009761574 | turquoise | 0 |
| CBX3 | 0.129264991 | 0.009789743 | turquoise | 0 |
| ALKBH7 | -0.10250628 | 0.009821172 | turquoise | 0 |
| KIDINS220 | 0.13050455 | 0.009870221 | turquoise | 0 |
| CHIC2 | 0.224388482 | 0.009956711 | turquoise | 0 |
| DCTPP1 | -0.115402442 | 0.00997 | turquoise | 0 |
| C11orf2 | -0.111574016 | 0.010242216 | turquoise | 0 |
| GLRX | 0.179827302 | 0.010271707 | turquoise | 0 |
| PRKCA | -0.151693071 | 0.010307447 | turquoise | 0 |
| C15orf44 | -0.136214146 | 0.010318256 | turquoise | 0 |
| C1GALT1C1 | 0.115097186 | 0.010344417 | turquoise | 0 |
| LOC100507507 | 0.138046202 | 0.010382196 | turquoise | 0 |
| KIAA0240 | 0.107922879 | 0.010431477 | turquoise | 0 |
| GAS5 | -0.25418706 | 0.010482636 | turquoise | 0 |
| RNF144B | 0.24678784 | 0.010691591 | turquoise | 0 |
| DMRTC1 | -0.646864201 | 0.010827822 | turquoise | 0 |
| FAM100B | 0.221415805 | 0.010916795 | turquoise | 0 |
| GATAD2A | 0.100157307 | 0.010922218 | turquoise | 0 |
| PSMD4 | 0.106999076 | 0.011067052 | turquoise | 0 |
| BCS1L | -0.141317403 | 0.011203648 | turquoise | 0 |
| ASXL1 | -0.092268662 | 0.01122886 | turquoise | 0 |
| LZTS2 | -0.111872131 | 0.01124652 | turquoise | 0 |
| LYRM1 | 0.201056357 | 0.011254634 | turquoise | 0 |
| CDS2 | 0.186006493 | 0.011307145 | turquoise | 0 |
| IK | 0.11421703 | 0.011348757 | turquoise | 0 |
| UBA3 | 0.171008293 | 0.011370958 | turquoise | 0 |
| UPF1 | 0.160951091 | 0.011412789 | turquoise | 0 |
| TMBIM6 | 0.145517713 | 0.011439136 | turquoise | 0 |
| C20orf24 | 0.196451083 | 0.01151346 | turquoise | 0 |
| TPPP3 | -0.470254451 | 0.011572964 | turquoise | 0 |
| DHRSX | 0.153805041 | 0.011581516 | turquoise | 0 |
| STEAP4 | 0.312915957 | 0.011771567 | turquoise | 0 |
| MPI | -0.112509095 | 0.011822215 | turquoise | 0 |
| PIGL | -0.201979227 | 0.011920337 | turquoise | 0 |
| ND5 | -0.139720416 | 0.011936471 | turquoise | 0 |
| DDT | -0.116783405 | 0.012003851 | turquoise | 0 |
| PRMT1 | -0.097512939 | 0.012116037 | turquoise | 0 |
| KIAA1191 | -0.118351925 | 0.012122707 | turquoise | 0 |
| NFE2 | 0.270170482 | 0.012186713 | turquoise | 0 |
| NAT9 | -0.12199422 | 0.012233758 | turquoise | 0 |
| C20orf106 | 0.192864807 | 0.012238503 | turquoise | 0 |
| ABI1 | 0.13121804 | 0.012316544 | turquoise | 0 |
| POP5 | -0.135499326 | 0.012341948 | turquoise | 0 |
| PCMTD1 | 0.179626777 | 0.012360728 | turquoise | 0 |
| DGCR2 | 0.128884332 | 0.012448691 | turquoise | 0 |
| P4HTM | -0.155639875 | 0.01245043 | turquoise | 0 |
| DENND5A | 0.162051502 | 0.012513997 | turquoise | 0 |
| KIAA0430 | 0.107181609 | 0.012591075 | turquoise | 0 |
| RPA3 | -0.13642003 | 0.012607137 | turquoise | 0 |
| LARS2 | -0.083768308 | 0.012640043 | turquoise | 0 |
| HTATIP2 | 0.120416699 | 0.012741775 | turquoise | 0 |
| FAM160B1 | 0.17862527 | 0.012754393 | turquoise | 0 |
| FTL | 0.214743128 | 0.01294311 | turquoise | 0 |
| XPNPEP1 | -0.115172272 | 0.012987552 | turquoise | 0 |
| AP3S1 | 0.130341867 | 0.013004899 | turquoise | 0 |
| MLKL | 0.12965618 | 0.013006287 | turquoise | 0 |
| HECA | 0.09278936 | 0.013029809 | turquoise | 0 |
| VARS2 | -0.0984782 | 0.013122593 | turquoise | 0 |
| GSK3A | 0.0786931 | 0.013203381 | turquoise | 0 |
| ZC3HAV1 | 0.186691269 | 0.013325532 | turquoise | 0 |
| DEXI | -0.121544681 | 0.013420722 | turquoise | 0 |
| CSTF3 | -0.140404786 | 0.013438537 | turquoise | 0 |
| PHLDB3 | -0.192088551 | 0.013450163 | turquoise | 0 |
| LYN | 0.187531092 | 0.013525084 | turquoise | 0 |
| TMEM102 | -0.148311161 | 0.013707569 | turquoise | 0 |
| OBFC2A | 0.186252369 | 0.013742903 | turquoise | 0 |
| EPHX2 | -0.295539147 | 0.01375685 | turquoise | 0 |
| PLSCR1 | 0.331920654 | 0.01382704 | turquoise | 0 |
| SUSD3 | -0.209663008 | 0.013869133 | turquoise | 0 |
| CLIC1 | 0.181486043 | 0.013869767 | turquoise | 0 |
| RGS19 | 0.166399174 | 0.013872559 | turquoise | 0 |
| OST4 | -0.169330787 | 0.013930964 | turquoise | 0 |
| SERGEF | -0.120648981 | 0.014342335 | turquoise | 0 |
| C14orf80 | -0.127788997 | 0.014361659 | turquoise | 0 |
| PALM2-AKAP2 | 0.121188579 | 0.014372851 | turquoise | 0 |
| ZNF84 | -0.16603656 | 0.014399088 | turquoise | 0 |
| SF3B14 | 0.110330589 | 0.014538033 | turquoise | 0 |
| PDDC1 | -0.137010531 | 0.014599403 | turquoise | 0 |
| CCNB1IP1 | -0.135879491 | 0.014681212 | turquoise | 0 |
| PI4KA | -0.117100328 | 0.01468697 | turquoise | 0 |
| AKR7L | -0.117108202 | 0.014739722 | turquoise | 0 |
| IER2 | 0.212595355 | 0.014818172 | turquoise | 0 |
| ZC3H4 | 0.096691124 | 0.01483133 | turquoise | 0 |
| FMNL1 | 0.124627732 | 0.014834404 | turquoise | 0 |
| TTC37 | -0.191867316 | 0.014847607 | turquoise | 0 |
| LSM5 | -0.157123758 | 0.015108664 | turquoise | 0 |
| NKAP | 0.088650686 | 0.015325319 | turquoise | 0 |
| NBPF3 | 0.130886337 | 0.015445299 | turquoise | 0 |
| TBC1D4 | -0.260006291 | 0.015450039 | turquoise | 0 |
| BCKDHB | -0.195664589 | 0.015466154 | turquoise | 0 |
| QARS | -0.135556368 | 0.01547823 | turquoise | 0 |
| DDX59 | 0.174741515 | 0.015525562 | turquoise | 0 |
| CCNDBP1 | 0.168045652 | 0.015533322 | turquoise | 0 |
| ARHGAP10 | -0.159342264 | 0.015652841 | turquoise | 0 |
| SH3KBP1 | 0.089013455 | 0.015677931 | turquoise | 0 |
| ZMPSTE24 | 0.142023444 | 0.015776456 | turquoise | 0 |
| TSPAN3 | -0.170281672 | 0.015787742 | turquoise | 0 |
| HERPUD1 | 0.117983359 | 0.015896688 | turquoise | 0 |
| ERV3-2 | 0.283943304 | 0.015904906 | turquoise | 0 |
| COQ4 | -0.126031963 | 0.016054171 | turquoise | 0 |
| UPF2 | 0.157505866 | 0.016177343 | turquoise | 0 |
| FCGRT | 0.141388322 | 0.016198909 | turquoise | 0 |
| LBR | 0.245871542 | 0.016289747 | turquoise | 0 |
| SNPH | -0.227800922 | 0.016398284 | turquoise | 0 |
| THOP1 | -0.094779485 | 0.01640829 | turquoise | 0 |
| GLTSCR2 | -0.137551352 | 0.016477416 | turquoise | 0 |
| APOO | -0.116079748 | 0.016491276 | turquoise | 0 |
| SUDS3 | 0.114677191 | 0.016611595 | turquoise | 0 |
| DPH2 | -0.116002996 | 0.01665415 | turquoise | 0 |
| CXorf23 | 0.155571697 | 0.01668724 | turquoise | 0 |
| ELMO1 | 0.208397837 | 0.016689169 | turquoise | 0 |
| ING1 | 0.129374384 | 0.016770544 | turquoise | 0 |
| DUS1L | -0.100607563 | 0.01698639 | turquoise | 0 |
| PIAS1 | 0.128830162 | 0.017019664 | turquoise | 0 |
| PRPSAP1 | -0.105597468 | 0.017153989 | turquoise | 0 |
| PPIL1 | -0.131520325 | 0.017202726 | turquoise | 0 |
| KLF8 | -0.368275102 | 0.017293089 | turquoise | 0 |
| TCEAL5 | -0.143359939 | 0.017651549 | turquoise | 0 |
| AKIRIN2-AS1 | 0.200509127 | 0.017661062 | turquoise | 0 |
| NACAP1 | -0.132552301 | 0.017829769 | turquoise | 0 |
| CHD1L | -0.119825726 | 0.018079423 | turquoise | 0 |
| METTL8 | -0.195831498 | 0.018121733 | turquoise | 0 |
| ARMC5 | -0.089166082 | 0.018187191 | turquoise | 0 |
| FAHD2A | -0.087511981 | 0.018347483 | turquoise | 0 |
| FAM113B | -0.213753688 | 0.018376409 | turquoise | 0 |
| GOLGA7 | 0.135046045 | 0.018419536 | turquoise | 0 |
| DNAJA1 | 0.114282553 | 0.018457592 | turquoise | 0 |
| C6orf26 | -0.132718783 | 0.018458338 | turquoise | 0 |
| ACTR2 | 0.147031997 | 0.018550559 | turquoise | 0 |
| GLIPR1 | 0.095267182 | 0.018736436 | turquoise | 0 |
| RFESD | -0.214704417 | 0.018909779 | turquoise | 0 |
| WDR74 | -0.080433993 | 0.018957703 | turquoise | 0 |
| PYCARD | 0.103522256 | 0.0189639 | turquoise | 0 |
| ZDHHC23 | -0.243224191 | 0.018982451 | turquoise | 0 |
| KRBA1 | -0.119834799 | 0.019038103 | turquoise | 0 |
| SNX11 | 0.168272482 | 0.019053108 | turquoise | 0 |
| SH3GL1 | 0.108512835 | 0.019155937 | turquoise | 0 |
| ZBTB5 | -0.121277767 | 0.019213729 | turquoise | 0 |
| PSKH1 | -0.072989794 | 0.019227641 | turquoise | 0 |
| RAB24 | 0.14519377 | 0.019600964 | turquoise | 0 |
| MIER1 | 0.116579494 | 0.01961349 | turquoise | 0 |
| H2AFY | 0.172725977 | 0.019701402 | turquoise | 0 |
| RAC2 | 0.107482597 | 0.019734955 | turquoise | 0 |
| TNKS2 | 0.171535457 | 0.019772101 | turquoise | 0 |
| C18orf25 | 0.158526843 | 0.019972738 | turquoise | 0 |
| ND3 | -0.15614152 | 0.020039403 | turquoise | 0 |
| RFFL | 0.131178056 | 0.02011442 | turquoise | 0 |
| RRM1 | -0.216340751 | 0.020242566 | turquoise | 0 |
| BIN1 | -0.162642846 | 0.020558576 | turquoise | 0 |
| KIAA0355 | -0.12548051 | 0.020641527 | turquoise | 0 |
| NBPF14 | 0.184877195 | 0.020727681 | turquoise | 0 |
| TMEM165 | 0.171505095 | 0.020920004 | turquoise | 0 |
| COBRA1 | -0.074374473 | 0.021157487 | turquoise | 0 |
| ARL8A | 0.161684041 | 0.021161225 | turquoise | 0 |
| TYSND1 | -0.133475864 | 0.021266211 | turquoise | 0 |
| TRUB2 | -0.081345412 | 0.021309475 | turquoise | 0 |
| FLT3LG | -0.318447467 | 0.021324832 | turquoise | 0 |
| NSUN5 | -0.081146626 | 0.021411021 | turquoise | 0 |
| NPIPL2 | -0.130327841 | 0.021492699 | turquoise | 0 |
| ARPC2 | 0.16421009 | 0.021566912 | turquoise | 0 |
| RPS28 | -0.164249467 | 0.021627845 | turquoise | 0 |
| VEGFB | -0.101027021 | 0.021863709 | turquoise | 0 |
| FAM50A | -0.082937561 | 0.021889045 | turquoise | 0 |
| MIS18A | -0.091022701 | 0.021934458 | turquoise | 0 |
| NHP2 | -0.100495623 | 0.022151661 | turquoise | 0 |
| TOP1P2 | 0.122921237 | 0.022159706 | turquoise | 0 |
| TCF20 | 0.163151742 | 0.022242473 | turquoise | 0 |
| OTUD3 | -0.216231831 | 0.022264233 | turquoise | 0 |
| PRPF8 | -0.104659625 | 0.022347369 | turquoise | 0 |
| USP11 | -0.104429005 | 0.022364313 | turquoise | 0 |
| EIF2AK2 | 0.282409063 | 0.022457974 | turquoise | 0 |
| PPWD1 | -0.081165968 | 0.022530066 | turquoise | 0 |
| ITCH | 0.092107097 | 0.022631737 | turquoise | 0 |
| GNB4 | 0.171255802 | 0.022634407 | turquoise | 0 |
| MAML1 | 0.114099964 | 0.022643777 | turquoise | 0 |
| TDP2 | 0.219386789 | 0.022664588 | turquoise | 0 |
| PAPOLA | 0.094714148 | 0.022796335 | turquoise | 0 |
| NDUFAF4 | -0.192983141 | 0.022820784 | turquoise | 0 |
| SRSF8 | -0.176454441 | 0.022836733 | turquoise | 0 |
| MAP3K5 | 0.142491918 | 0.02283736 | turquoise | 0 |
| ARRDC2 | -0.116397438 | 0.022953586 | turquoise | 0 |
| RRP1B | -0.113065773 | 0.022957803 | turquoise | 0 |
| MRPS21 | -0.140692028 | 0.023053282 | turquoise | 0 |
| CD63 | 0.199272208 | 0.023113282 | turquoise | 0 |
| ZSWIM6 | 0.11686537 | 0.023115957 | turquoise | 0 |
| HPS4 | -0.112792031 | 0.023154914 | turquoise | 0 |
| SPOPL | 0.158010427 | 0.023368322 | turquoise | 0 |
| CFLAR | 0.1432025 | 0.023398985 | turquoise | 0 |
| ZNF783 | -0.166126638 | 0.023437443 | turquoise | 0 |
| FXYD5 | -0.106067732 | 0.023464511 | turquoise | 0 |
| FUK | -0.121807828 | 0.023495373 | turquoise | 0 |
| PYCR2 | -0.098316657 | 0.023516065 | turquoise | 0 |
| CCDC47 | 0.072465159 | 0.023542899 | turquoise | 0 |
| URGCP | -0.132424682 | 0.023774368 | turquoise | 0 |
| TIMP2 | 0.179735437 | 0.023791655 | turquoise | 0 |
| CDKL5 | 0.157824801 | 0.023837878 | turquoise | 0 |
| TMEM105 | 0.215373323 | 0.024277456 | turquoise | 0 |
| IFI16 | 0.236129185 | 0.024540755 | turquoise | 0 |
| RP2 | 0.186513992 | 0.02457789 | turquoise | 0 |
| CAMSAP1 | -0.208084213 | 0.024723079 | turquoise | 0 |
| GADD45GIP1 | -0.08890347 | 0.02499329 | turquoise | 0 |
| TEX10 | -0.143250175 | 0.025176156 | turquoise | 0 |
| RILPL2 | 0.135389495 | 0.025235933 | turquoise | 0 |
| TBK1 | 0.122959109 | 0.025303512 | turquoise | 0 |
| FAM120A | 0.097738982 | 0.025309199 | turquoise | 0 |
| MAGEF1 | -0.138402968 | 0.025326146 | turquoise | 0 |
| LOC441455 | 0.144195781 | 0.025350543 | turquoise | 0 |
| PREX1 | 0.218520408 | 0.025437437 | turquoise | 0 |
| MYBBP1A | -0.106024053 | 0.025504612 | turquoise | 0 |
| AZI1 | -0.090787724 | 0.025572032 | turquoise | 0 |
| TMEM8B | -0.153885521 | 0.025815692 | turquoise | 0 |
| HINT2 | -0.089257375 | 0.025855888 | turquoise | 0 |
| ARHGDIB | 0.176021868 | 0.025887484 | turquoise | 0 |
| SUN1 | -0.17024228 | 0.026012925 | turquoise | 0 |
| ZNF48 | -0.108387912 | 0.026103288 | turquoise | 0 |
| ZMAT2 | 0.089286367 | 0.02633748 | turquoise | 0 |
| RPL34 | -0.139872013 | 0.026459377 | turquoise | 0 |
| BRD2 | 0.092488582 | 0.026534774 | turquoise | 0 |
| C10orf12 | 0.119600409 | 0.026601587 | turquoise | 0 |
| PRPF31 | -0.096505825 | 0.026744377 | turquoise | 0 |
| GTF2B | 0.111817632 | 0.026794167 | turquoise | 0 |
| ITPRIP | 0.14171505 | 0.026840937 | turquoise | 0 |
| NPTN | 0.22017465 | 0.026884588 | turquoise | 0 |
| MOB1A | 0.093269406 | 0.026959175 | turquoise | 0 |
| NFIL3 | 0.26235909 | 0.027007134 | turquoise | 0 |
| ACBD6 | -0.090051731 | 0.027127746 | turquoise | 0 |
| JUNB | 0.199852252 | 0.027281979 | turquoise | 0 |
| DNAJC15 | -0.163228469 | 0.027321936 | turquoise | 0 |
| NFYA | 0.095025735 | 0.027325357 | turquoise | 0 |
| AKIRIN2 | 0.140092769 | 0.027462625 | turquoise | 0 |
| SEMA4C | -0.149262757 | 0.027498839 | turquoise | 0 |
| SGSM3 | -0.109773704 | 0.027635463 | turquoise | 0 |
| CEBPB | 0.201488721 | 0.027669766 | turquoise | 0 |
| IKBKAP | -0.130463448 | 0.027811305 | turquoise | 0 |
| ANO9 | -0.246931559 | 0.027861688 | turquoise | 0 |
| ZNF10 | -0.174455957 | 0.027909926 | turquoise | 0 |
| TPM3 | 0.100968307 | 0.027937977 | turquoise | 0 |
| TXN | 0.23357366 | 0.028041565 | turquoise | 0 |
| LOC439949 | -0.224214855 | 0.028052338 | turquoise | 0 |
| PPAP2B | 0.230634303 | 0.028233636 | turquoise | 0 |
| LOC392288 | 0.180037673 | 0.028515256 | turquoise | 0 |
| TMSB4X | 0.118110209 | 0.028767877 | turquoise | 0 |
| TROVE2 | 0.102582476 | 0.02887846 | turquoise | 0 |
| N4BP2L2 | 0.083816726 | 0.028935248 | turquoise | 0 |
| OSBPL2 | 0.141152814 | 0.028947631 | turquoise | 0 |
| ATF7IP2 | -0.205937617 | 0.028987989 | turquoise | 0 |
| TP53INP1 | 0.146675556 | 0.029031708 | turquoise | 0 |
| PRO2852 | 0.241742621 | 0.029120329 | turquoise | 0 |
| C3orf26 | -0.168481086 | 0.029161457 | turquoise | 0 |
| MLH1 | -0.131885067 | 0.029282631 | turquoise | 0 |
| CARD8 | 0.096212117 | 0.029514077 | turquoise | 0 |
| CDC42SE1 | 0.143081963 | 0.029514453 | turquoise | 0 |
| PELI1 | 0.125155828 | 0.029562621 | turquoise | 0 |
| PHLPP2 | -0.194136696 | 0.029834219 | turquoise | 0 |
| WDR54 | -0.102020975 | 0.029941863 | turquoise | 0 |
| RARA | 0.131062691 | 0.029954988 | turquoise | 0 |
| UBE2G2 | -0.081321336 | 0.030033922 | turquoise | 0 |
| IL13RA1 | 0.239763976 | 0.030070297 | turquoise | 0 |
| UBXN4 | 0.122547675 | 0.030095662 | turquoise | 0 |
| ZNF746 | 0.148791685 | 0.03018824 | turquoise | 0 |
| TAB2 | 0.109313442 | 0.030201166 | turquoise | 0 |
| FBXO38 | 0.135552036 | 0.030254181 | turquoise | 0 |
| CNIH4 | 0.165680615 | 0.030278133 | turquoise | 0 |
| IL11RA | -0.164695246 | 0.030372548 | turquoise | 0 |
| DNAJA2 | 0.130946585 | 0.030391111 | turquoise | 0 |
| NICN1 | -0.113607002 | 0.030548577 | turquoise | 0 |
| RPL22 | -0.141379027 | 0.030579455 | turquoise | 0 |
| TRIM8 | 0.099396675 | 0.030607699 | turquoise | 0 |
| IGIP | -0.156768288 | 0.030650894 | turquoise | 0 |
| SUMO1 | 0.169079052 | 0.030921437 | turquoise | 0 |
| HLA-A | 0.144117037 | 0.030983067 | turquoise | 0 |
| ZNF689 | -0.134136129 | 0.031097523 | turquoise | 0 |
| S100A6 | 0.233147677 | 0.031181596 | turquoise | 0 |
| RNF130 | 0.138723768 | 0.031259095 | turquoise | 0 |
| NAB1 | 0.106737349 | 0.031285987 | turquoise | 0 |
| ZNF777 | -0.122161075 | 0.031357705 | turquoise | 0 |
| LOC100652733 | -0.178257764 | 0.031407312 | turquoise | 0 |
| PPP1R3D | 0.192688006 | 0.031500015 | turquoise | 0 |
| XRCC4 | 0.135178643 | 0.031674999 | turquoise | 0 |
| TRA2A | 0.11688193 | 0.03187289 | turquoise | 0 |
| APEX1 | -0.101267946 | 0.031882102 | turquoise | 0 |
| CYB5R4 | 0.173603971 | 0.032081822 | turquoise | 0 |
| MADD | -0.110037281 | 0.032107089 | turquoise | 0 |
| BPTF | 0.061715136 | 0.032123217 | turquoise | 0 |
| PARP6 | -0.078798657 | 0.032169264 | turquoise | 0 |
| MCTP2 | 0.229463888 | 0.032202726 | turquoise | 0 |
| C1orf63 | 0.103777022 | 0.032240005 | turquoise | 0 |
| PLCXD1 | -0.140205717 | 0.032392578 | turquoise | 0 |
| COG2 | -0.121346461 | 0.032503103 | turquoise | 0 |
| CATSPER2 | -0.175984322 | 0.032510447 | turquoise | 0 |
| LOC100505869 | 0.412947999 | 0.032516787 | turquoise | 0 |
| NUDCD1 | -0.168855911 | 0.032597984 | turquoise | 0 |
| FAM160B2 | -0.122739414 | 0.032611948 | turquoise | 0 |
| CCDC28A | 0.083728703 | 0.032645783 | turquoise | 0 |
| CHMP5 | 0.173009481 | 0.032664606 | turquoise | 0 |
| BRI3 | 0.182998548 | 0.03279305 | turquoise | 0 |
| MTR | -0.138310532 | 0.032817694 | turquoise | 0 |
| R3HDM2 | 0.06894744 | 0.032971068 | turquoise | 0 |
| ZFAND5 | 0.085816477 | 0.033185594 | turquoise | 0 |
| NUP85 | -0.082127258 | 0.033215525 | turquoise | 0 |
| CRYGS | -0.146481934 | 0.03336205 | turquoise | 0 |
| KDM2A | 0.118599576 | 0.033367864 | turquoise | 0 |
| RNF19B | 0.199211083 | 0.033539245 | turquoise | 0 |
| SHPRH | -0.096314962 | 0.033612065 | turquoise | 0 |
| ZC3HC1 | -0.089496304 | 0.033721528 | turquoise | 0 |
| DOK1 | 0.096339849 | 0.033930514 | turquoise | 0 |
| SERF1B | -0.098601324 | 0.03397661 | turquoise | 0 |
| RAP1A | 0.162215927 | 0.03408076 | turquoise | 0 |
| SSH2 | 0.19417325 | 0.03427872 | turquoise | 0 |
| ECSIT | -0.081301453 | 0.034353548 | turquoise | 0 |
| YWHAZ | 0.105909108 | 0.034443259 | turquoise | 0 |
| EIF1 | 0.145693673 | 0.03460135 | turquoise | 0 |
| HMGB2 | 0.303741928 | 0.034659338 | turquoise | 0 |
| AKR7A2 | -0.114743775 | 0.034754473 | turquoise | 0 |
| CTNNB1 | 0.106420903 | 0.034779448 | turquoise | 0 |
| CKLF | 0.150464251 | 0.034786806 | turquoise | 0 |
| CRISPLD2 | 0.231523255 | 0.034850214 | turquoise | 0 |
| MTERFD2 | -0.150583354 | 0.034887065 | turquoise | 0 |
| GRB10 | 0.416032701 | 0.034959594 | turquoise | 0 |
| ZNF7 | -0.075664537 | 0.035085195 | turquoise | 0 |
| MSL2 | 0.119538419 | 0.035105126 | turquoise | 0 |
| SCLT1 | 0.127821074 | 0.03513292 | turquoise | 0 |
| PKIA | -0.221187239 | 0.035142901 | turquoise | 0 |
| GCA | 0.316267816 | 0.035209476 | turquoise | 0 |
| TANK | 0.149344503 | 0.03530521 | turquoise | 0 |
| SNRK | 0.111121762 | 0.035329405 | turquoise | 0 |
| TUBGCP6 | -0.114344487 | 0.03535126 | turquoise | 0 |
| CDC5L | 0.042365994 | 0.035498567 | turquoise | 0 |
| CDC42EP3 | 0.151079412 | 0.035506783 | turquoise | 0 |
| RXRA | 0.112533415 | 0.035520448 | turquoise | 0 |
| 7-Mar | 0.158849842 | 0.035581655 | turquoise | 0 |
| CHD1 | 0.096694062 | 0.035647447 | turquoise | 0 |
| POLE3 | -0.094874881 | 0.035740618 | turquoise | 0 |
| DHX37 | -0.090342362 | 0.035755571 | turquoise | 0 |
| TSG101 | 0.09228538 | 0.035813456 | turquoise | 0 |
| CTNNBL1 | -0.086112063 | 0.035866128 | turquoise | 0 |
| PCBP1 | 0.122677699 | 0.035908833 | turquoise | 0 |
| RPL35A | -0.092117631 | 0.035969653 | turquoise | 0 |
| HSPA1A | 0.227797604 | 0.036014662 | turquoise | 0 |
| IDI1 | 0.244973554 | 0.036087918 | turquoise | 0 |
| RPS12 | -0.163938011 | 0.036106025 | turquoise | 0 |
| RABL2A | -0.137853856 | 0.036317636 | turquoise | 0 |
| THRAP3 | 0.082210309 | 0.036346283 | turquoise | 0 |
| ANKRD13A | 0.130264562 | 0.036441116 | turquoise | 0 |
| NHP2L1 | -0.107995585 | 0.036443083 | turquoise | 0 |
| MGC4473 | 0.189445233 | 0.036706279 | turquoise | 0 |
| ZFAND3 | 0.133506132 | 0.036826809 | turquoise | 0 |
| PXN | 0.142777751 | 0.036911162 | turquoise | 0 |
| PLXNC1 | 0.213609021 | 0.036943851 | turquoise | 0 |
| UBE2W | 0.13351279 | 0.036981375 | turquoise | 0 |
| ACTR3 | 0.123670548 | 0.037061131 | turquoise | 0 |
| GPBP1L1 | 0.108891388 | 0.037084896 | turquoise | 0 |
| CASC3 | 0.147534727 | 0.037111131 | turquoise | 0 |
| ZNF438 | 0.160746898 | 0.037159003 | turquoise | 0 |
| H2AFZ | 0.120354249 | 0.037248255 | turquoise | 0 |
| ZNF230 | 0.22750335 | 0.037410746 | turquoise | 0 |
| NFE4 | 0.368102665 | 0.037471049 | turquoise | 0 |
| LASP1 | 0.11933289 | 0.037544731 | turquoise | 0 |
| TMEM140 | 0.13517468 | 0.037673944 | turquoise | 0 |
| CLPX | 0.066988662 | 0.037727466 | turquoise | 0 |
| NSMAF | 0.155750758 | 0.037804785 | turquoise | 0 |
| RBM8A | 0.085140914 | 0.037823666 | turquoise | 0 |
| ADIPOR1 | 0.161284658 | 0.038540935 | turquoise | 0 |
| C1orf35 | -0.133750231 | 0.038613585 | turquoise | 0 |
| VPS11 | -0.084264848 | 0.038625305 | turquoise | 0 |
| DUSP1 | 0.25969598 | 0.038724861 | turquoise | 0 |
| FOXN3 | 0.098492891 | 0.038809545 | turquoise | 0 |
| WDR59 | -0.090246553 | 0.039318804 | turquoise | 0 |
| GUSBP4 | -0.213760748 | 0.039583609 | turquoise | 0 |
| GMCL1 | 0.129228739 | 0.039598402 | turquoise | 0 |
| ABCF1 | -0.090281896 | 0.039730739 | turquoise | 0 |
| LATS2 | 0.12096194 | 0.039746655 | turquoise | 0 |
| XRCC5 | 0.074491514 | 0.039768838 | turquoise | 0 |
| LINC00246A | -0.278867497 | 0.039922441 | turquoise | 0 |
| PLEKHJ1 | -0.058550218 | 0.039978709 | turquoise | 0 |
| POLG2 | -0.098184907 | 0.040135931 | turquoise | 0 |
| MRPL44 | 0.094408338 | 0.04042331 | turquoise | 0 |
| MLYCD | -0.115874436 | 0.040494812 | turquoise | 0 |
| UBE2J1 | 0.212765861 | 0.040804302 | turquoise | 0 |
| NDUFS8 | -0.105834768 | 0.040807093 | turquoise | 0 |
| CTAGE7P | 0.10113556 | 0.040864372 | turquoise | 0 |
| ACVR1C | -0.313775119 | 0.040996346 | turquoise | 0 |
| DHX35 | -0.101089547 | 0.041023829 | turquoise | 0 |
| BCL11B | -0.113476509 | 0.041024894 | turquoise | 0 |
| METTL13 | -0.077586196 | 0.041072758 | turquoise | 0 |
| MSN | 0.08631396 | 0.041145167 | turquoise | 0 |
| LOC100652828 | -0.1557166 | 0.041191269 | turquoise | 0 |
| CDKN2D | 0.190007552 | 0.041335318 | turquoise | 0 |
| MAP3K8 | 0.124540377 | 0.041504868 | turquoise | 0 |
| DIABLO | -0.055763478 | 0.041641328 | turquoise | 0 |
| ZNF217 | 0.147940829 | 0.041709696 | turquoise | 0 |
| SHMT2 | -0.107274379 | 0.041777763 | turquoise | 0 |
| ARNT | 0.104910661 | 0.041837192 | turquoise | 0 |
| HSD17B11 | 0.162495435 | 0.04186686 | turquoise | 0 |
| MAGOHB | -0.11857843 | 0.041882845 | turquoise | 0 |
| ZADH2 | -0.11108557 | 0.041916713 | turquoise | 0 |
| ITFG2 | -0.11567876 | 0.042157837 | turquoise | 0 |
| MAP2K6 | 0.269916388 | 0.042170084 | turquoise | 0 |
| FIBP | -0.080721797 | 0.042175213 | turquoise | 0 |
| MAL | -0.260843186 | 0.042178242 | turquoise | 0 |
| SELT | 0.108712797 | 0.042251161 | turquoise | 0 |
| NPL | 0.157457844 | 0.042552484 | turquoise | 0 |
| AKAP1 | -0.113698872 | 0.042683943 | turquoise | 0 |
| MGRN1 | 0.140743735 | 0.042698354 | turquoise | 0 |
| STX7 | 0.144351926 | 0.042722083 | turquoise | 0 |
| OFD1 | -0.114873729 | 0.042769425 | turquoise | 0 |
| DIEXF | -0.146530972 | 0.042786553 | turquoise | 0 |
| MAT2B | 0.108671833 | 0.042824272 | turquoise | 0 |
| HIST2H2AB | 0.18316727 | 0.042923767 | turquoise | 0 |
| PHF19 | -0.075491302 | 0.042928471 | turquoise | 0 |
| MAP2K3 | 0.143256213 | 0.042983288 | turquoise | 0 |
| ALG9 | -0.115574498 | 0.043162245 | turquoise | 0 |
| RHOA | 0.133636315 | 0.043271861 | turquoise | 0 |
| SSH3 | 0.126290505 | 0.043323187 | turquoise | 0 |
| STAT3 | 0.18361198 | 0.043645921 | turquoise | 0 |
| RAB21 | 0.145024391 | 0.043665117 | turquoise | 0 |
| ADCK5 | -0.117282056 | 0.043682872 | turquoise | 0 |
| UBC | 0.194670153 | 0.043784337 | turquoise | 0 |
| CTAGE4 | 0.077101004 | 0.043809179 | turquoise | 0 |
| OXNAD1 | -0.171451088 | 0.043855953 | turquoise | 0 |
| LOC284889 | -0.105334077 | 0.043859775 | turquoise | 0 |
| FRAT2 | 0.15429582 | 0.043875385 | turquoise | 0 |
| GPRASP1 | -0.275730405 | 0.043880012 | turquoise | 0 |
| AFG3L1P | -0.10742875 | 0.044028661 | turquoise | 0 |
| DDX5 | 0.115573502 | 0.044089084 | turquoise | 0 |
| ROCK1 | 0.131245495 | 0.044107775 | turquoise | 0 |
| SRPK1 | 0.277419812 | 0.044330623 | turquoise | 0 |
| RANGRF | -0.115341472 | 0.044571023 | turquoise | 0 |
| IFNGR2 | 0.147778055 | 0.044766022 | turquoise | 0 |
| GHITM | 0.063026345 | 0.044838244 | turquoise | 0 |
| KPNA5 | -0.23686487 | 0.044909586 | turquoise | 0 |
| TMEM50A | 0.107353198 | 0.044964113 | turquoise | 0 |
| DR1 | 0.082818905 | 0.045161763 | turquoise | 0 |
| TCTN3 | -0.130645903 | 0.045171764 | turquoise | 0 |
| FAM8A1 | 0.196253733 | 0.045248727 | turquoise | 0 |
| ABLIM2 | -0.270126869 | 0.045258094 | turquoise | 0 |
| SARM1 | -0.177995699 | 0.045395162 | turquoise | 0 |
| DDX51 | -0.072482678 | 0.04547123 | turquoise | 0 |
| ARL6IP1 | 0.103105833 | 0.045480695 | turquoise | 0 |
| SLU7 | 0.068101308 | 0.045485524 | turquoise | 0 |
| IDH3G | -0.079508775 | 0.045506613 | turquoise | 0 |
| UROD | -0.078015142 | 0.045517765 | turquoise | 0 |
| NEURL4 | -0.066482385 | 0.045615672 | turquoise | 0 |
| ATP6V1G1 | 0.118366924 | 0.045712589 | turquoise | 0 |
| SLC8A1 | 0.226298495 | 0.04576395 | turquoise | 0 |
| PRDM2 | 0.084568671 | 0.04579597 | turquoise | 0 |
| AGPAT5 | -0.136191535 | 0.045940125 | turquoise | 0 |
| EDEM2 | 0.123345598 | 0.046940323 | turquoise | 0 |
| AKTIP | 0.150813047 | 0.046997222 | turquoise | 0 |
| NARG2 | -0.120631365 | 0.047464241 | turquoise | 0 |
| TPCN1 | -0.155988561 | 0.047491438 | turquoise | 0 |
| ADRBK1 | 0.082357509 | 0.047647507 | turquoise | 0 |
| ALG8 | -0.120123728 | 0.047657797 | turquoise | 0 |
| TMEM25 | -0.175828943 | 0.047871941 | turquoise | 0 |
| ZIK1 | -0.203621463 | 0.047917388 | turquoise | 0 |
| ASPH | 0.308578743 | 0.048079764 | turquoise | 0 |
| PLEKHM3 | 0.084269504 | 0.048080559 | turquoise | 0 |
| ANK3 | -0.209396404 | 0.048130961 | turquoise | 0 |
| PPP1CB | 0.144769987 | 0.048135405 | turquoise | 0 |
| MTO1 | -0.090303034 | 0.048248075 | turquoise | 0 |
| PHACTR4 | -0.0923566 | 0.048252591 | turquoise | 0 |
| EPB41L4A-AS1 | -0.148785239 | 0.048280778 | turquoise | 0 |
| HADH | -0.086244547 | 0.048286161 | turquoise | 0 |
| HMBS | -0.114210354 | 0.048333952 | turquoise | 0 |
| ATP6V0E1 | 0.12233643 | 0.048343878 | turquoise | 0 |
| RSAD1 | -0.111686805 | 0.048390439 | turquoise | 0 |
| POLD1 | -0.064728742 | 0.048426078 | turquoise | 0 |
| FAS | 0.178416114 | 0.048633901 | turquoise | 0 |
| TRIP12 | 0.092129793 | 0.048644827 | turquoise | 0 |
| FAM27E3 | 0.284742529 | 0.048655739 | turquoise | 0 |
| MCL1 | 0.151944262 | 0.048681451 | turquoise | 0 |
| NELL2 | -0.293381349 | 0.048704713 | turquoise | 0 |
| C17orf70 | -0.104213304 | 0.048720517 | turquoise | 0 |
| IGLL5 | 0.177927924 | 0.048726007 | turquoise | 0 |
| MYCBP2 | -0.104726275 | 0.048744671 | turquoise | 0 |
| BOLA1 | -0.111267572 | 0.048952055 | turquoise | 0 |
| SPPL2A | 0.084387778 | 0.048959564 | turquoise | 0 |
| ALG1L | -0.099334787 | 0.049098813 | turquoise | 0 |
| CHMP1B | 0.132720936 | 0.049531601 | turquoise | 0 |
| GRB2 | 0.103113239 | 0.049866644 | turquoise | 0 |
| FAM91A1 | 0.100262354 | 0.049876277 | turquoise | 0 |
| HLA-B | 0.25186331 | 0.001018149 | turquoise | 1 |
| HLA-F | 0.215188386 | 0.00126609 | turquoise | 1 |
| CASP4 | 0.257463169 | 0.003321053 | turquoise | 1 |
| HMGN2P46 | 0.176526412 | 0.007519181 | turquoise | 1 |
| SNRPE | -0.1875284 | 0.008103803 | turquoise | 1 |
| TALDO1 | 0.221069975 | 0.012775393 | turquoise | 1 |
| HLA-H | 0.18681533 | 0.014743944 | turquoise | 1 |
| CASP5 | 0.166927342 | 0.017576322 | turquoise | 1 |
| CMTM6 | 0.193811383 | 0.021166006 | turquoise | 1 |
| TRMT5 | 0.116939261 | 0.021496578 | turquoise | 1 |
| IFITM4P | 0.200056564 | 0.02176319 | turquoise | 1 |
| ARPC3 | 0.172974779 | 0.029663205 | turquoise | 1 |
| MYL12A | 0.169356028 | 0.034458384 | turquoise | 1 |
| IFITM3 | 0.204919642 | 0.035141956 | turquoise | 1 |
| MYL12B | 0.133902795 | 0.035798237 | turquoise | 1 |
| COX6C | -0.162084727 | 0.042782522 | turquoise | 1 |
| HLA-C | 0.280827476 | 0.000740045 | turquoise | 2 |
| HLA-J | 0.29451586 | 0.001203708 | turquoise | 2 |
| ACTB | 0.239038859 | 0.01073995 | turquoise | 2 |
| HIST1H2AD | 0.26496569 | 0.015494 | turquoise | 2 |
| HMGN2 | 0.131704572 | 0.017835449 | turquoise | 2 |
| HIST3H2A | 0.211240844 | 0.022088796 | turquoise | 2 |
| POTEM | 0.17026212 | 0.033779827 | turquoise | 2 |
| HIST1H2AJ | 0.175496705 | 0.043217567 | turquoise | 2 |
| POTEKP | 0.167616788 | 0.049110458 | turquoise | 2 |
| HLA-E | 0.225256458 | 0.002469105 | turquoise | 3 |
| HIST1H3C | 0.225908187 | 0.01601822 | turquoise | 3 |
| HIST2H3D | 0.223223812 | 0.0156723 | turquoise | 4 |
| H3F3B | 0.236581948 | 0.029414012 | turquoise | 4 |
| H3F3C | 0.242560151 | 0.045275322 | turquoise | 4 |
| HIST3H3 | 0.199060752 | 0.040708125 | turquoise | 6 |

|  |
| --- |
