# Supplementary material for: NCF2, MYO1F, S1PR4, and FCN1 as potential noninvasive diagnostic biomarkers in patients with obstructive coronary artery: A weighted gene co‐expression network analysis
Source: J Cell Biochem. 2019 Jun 27;120(10):18219–35. doi: 10.1002/jcb.29128 (PMC6771964; doi:10.1002/jcb.29128)
Supplement: Supplementary file 4 — Supporting information [file JCB-120--s003.doc]

| **Supplemental Table 3** Top 3 GO BP terms enriched by 13 hub-genes | | | |
| --- | --- | --- | --- |
| Term | Count | P value | Gene in the GO term |
| immune system process (GO.0002376) | 11 | 0.00000222 | AMICA1,CLEC4E,CSF1R,FCGR3B,FCN1,MNDA,MYO1F,NCF2,S100A9,S1PR4,TLR2 |
| immune response (GO.0006955) | 9 | 0.0000325 | CLEC4E,CSF1R,FCGR3B,FCN1,MYO1F,NCF2,S100A9,S1PR4,TLR2 |
| regulation of immune system process (GO.0002682) | 8 | 0.000598 | AMICA1,CLEC4E,CSF1R,FCGR3B,FCN1,MNDA,MYO1F,TLR2 |
